# Enterotoxigenicity and Antimicrobial Resistance of *Staphylococcus aureus* Isolated from Retail Food in China

**DOI:** 10.3389/fmicb.2017.02256

**Published:** 2017-11-21

**Authors:** Wei Wang, Zulqarnain Baloch, Tao Jiang, Cunshan Zhang, Zixin Peng, Fengqin Li, Séamus Fanning, Aiguo Ma, Jin Xu

**Affiliations:** ^1^School of Public Health, Qingdao University, Qingdao, China; ^2^Key Laboratory of Food Safety Risk Assessment, Ministry of Health, China National Center for Food Safety Risk Assessment, Beijing, China; ^3^College of Veterinary Medicine, South China Agricultural University, Guangzhou, China; ^4^Kuiwen District Center for Disease Control and Prevention, Weifang, China; ^5^UCD-Centre for Food Safety, School of Public Health, Physiotherapy and Sports Science, University College Dublin, Dublin, Ireland; ^6^Institute for Global Food Security, School of Biological Sciences, Queen's University Belfast, Belfast, United Kingdom

**Keywords:** retail foods, *Staphylococcus aureus*, enterotoxingenicity, methicillin-resistant *Staphylococcus aureus*, antimicrobial resistance

## Abstract

*Staphylococcus aureus* is one of the most common causes of zoonotic agent in the world, which are attributable to the contamination of food with enterotoxins. In this study, a total of 1,150 *S. aureus* isolates were cultured from 27,000 retail foods items from 203 cities of 24 provinces in China in 2015 and were test for antimicrobial susceptibility. Additionally, the role of the genes responsible for the staphylococcal enterotoxins (SEA to SEE), methicillin resistance (*mecA*) and the toxigenic capabilities were also assessed. The results showed that 4.3% retail foods were contaminated with *S. aureus*, and 7.9% retail foods isolates were *mecA* positive. Some 97.6% of *S. aureus* isolates were resistant to at least one antimicrobial compound, and 57.5% of these were multi drug resistant (MDR). Resistance to penicillin (83.7%, 963/1,150), was common, followed by linezolid (67.7%, 778/1,150) and erythromycin (52.1%, 599/1,150). The isolates cultured from raw meats showed high levels of resistant to tetracycline (42.8%), ciprofloxacin (17.4%), and chloramphenicol (12.0%) and expressed a MDR phenotype (62.4%). A total of 29.7% *S. aureus* isolates harbored the classical SEs genes (*sea, seb, sec*, and *sed*). The *sea* and *seb* genes were the most frequent SEs genes detected. Of note, 22% of the SEs genes positive *S. aureus* harbored two or three SEs genes, and 16 isolates were confirmed with the capacity to simultaneously produce two or three enterotoxin types. Moreover, nearly 50% of the MRSA isolates were positive for at least one SE gene in this study. Therefore, it is important to monitor the antimicrobial susceptibility and enterotoxigenicity of MDR *S. aureus* and MRSA in the food chain and to use these data to develop food safety measures, designed to reduce the contamination and transmission of this bacterium.

## Introduction

*Staphylococcus aureus* (*S. aureus*) is well a known opportunistic pathogen widely present in a broad host range, including human beings and food producing animals, such as pigs, cows, goats, chickens and ducks (Lowder et al., 2009; Hasman et al., 2010; Gao et al., 2012; Wang et al., 2017). *S. aureus* can cause various infections, ranging from superficial skin and soft tissue infections to life threatening diseases, such as septicemia, necrotizing fasciitis, endocarditis, and necrotizing pneumonia (Krishna and Millerm, 2012; Chen and Huang, 2014; Rodríguez-Lázaro et al., 2015). This bacterium has the potential to contaminate animal products and may enter the food chain, during processing, preparation, wrapping, mincing, and storage. The wide use of antibiotics has led to the emergence of multi drug resistant strains (MDR), particularly methicillin-resistant *Staphylococcus aureus* (MRSA) (World Health Organization, 2012; Fox et al., 2017). These drug-resistant bacteria can readily be transferred to humans *via* food, resulting in potential infectious the subsequent treatment of which may be compromised through the narrowing of chemotherapeutic options for clinicians (Hammad et al., 2012). It was reported that the prevalence of *S. aureus* in retail foods in China was 12.5% (69/550), and in some reports, this value was much higher in certain ready-to-eat (RTE) foods (Wang et al., 2014; Yang et al., 2016; Rong et al., 2017). Additionally, not only MRSA, methicillin-susceptible *Staphylococcus aureus* (MSSA) identified among food for sale at retail also showed high levels of resistant to several antimicrobial agents, resulting in a major public health concern in China.

The pathogenicity of *S. aureus* is related to various virulence factors. Heat stable staphylococcal enterotoxins (SEs) produced by enterotoxigenic strains of *S. aureus* is considered as one major global cause of food poisoning (Le Loir et al., 2003). Of note, several food materials, especially pork, beef, mutton, poultry, and eggs, and their products could be contaminated with *S. aureus* during farming or slaughtering process. Additionally, food handlers carrying *S. aureus* on their bodies or gloves can also contaminate food (Crago et al., 2012). Once these species possess toxigenic capabilities, they may cause outbreaks of food borne illness. Currently, 23 enterotoxins have been described. The five most well studied are considered classical enterotoxins (SEA, SEB, SEC, SED, and SEE) (da Silva Sdos et al., 2015), encoded by specific enterotoxin genes denoted as *sea* to *see*. The disease caused by SEs is characterized by a short incubation period (an average of 4.4 h), nausea, violent vomiting, abdominal cramps, headache, and diarrhea. Although this disease is usually a self-limiting illness, death occasionally occurs among more susceptible individuals, such as children and the elderly population (Tarekgne et al., 2016). In 2012, SEs was responsible for 346 food borne outbreaks (FBOs) in the European Union, representing 6.4% of all outbreaks reported there (Macori et al., 2016). In the United States, staphylococcal food poisoning is estimated to account for 241,000 illnesses requiring hospitalization annually (Scallan et al., 2011; Byrd-Bredbenner et al., 2013). It is estimated that 20–25% of food borne bacterial outbreaks are caused by *S. aureus* in China (Wang et al., 2014). The increasing incidence of staphylococcal food poisoning has raised serious food safety concerns worldwide (Pu et al., 2011).

Molecular biological based and immunological techniques are considered important tools for investigating *S. aureus* contaminations (Argudín et al., 2012; Gholamzad et al., 2015). Therefore, the identification of staphylococcal enterotoxins (SEs) and drug-resistance genes has been extensively reported for *S. aureus*. Recently, the presence of SEs and drug-resistance genes have also been reported in retail food samples in some regions in China (Zhao et al., 2012; Wang et al., 2014; Yang et al., 2016). However, no national level surveillance on the prevalence, enterotoxigenicity and antimicrobial resistance of *S. aureus* in retail foods has been reported to date. Therefore, the present study was designed to evaluate the prevalence of *S. aureus* derived from various retail food samples in China. This study was designed to characterize the isolated strains based on their production of SEs and antimicrobial-resistance pattern by evaluating the distribution of the *mecA, sea* to *see* genes and the toxigenic capabilities of *S. aureus* isolates in retail food.

## Materials and methods

### Bacterial isolates

**Figure 1:**
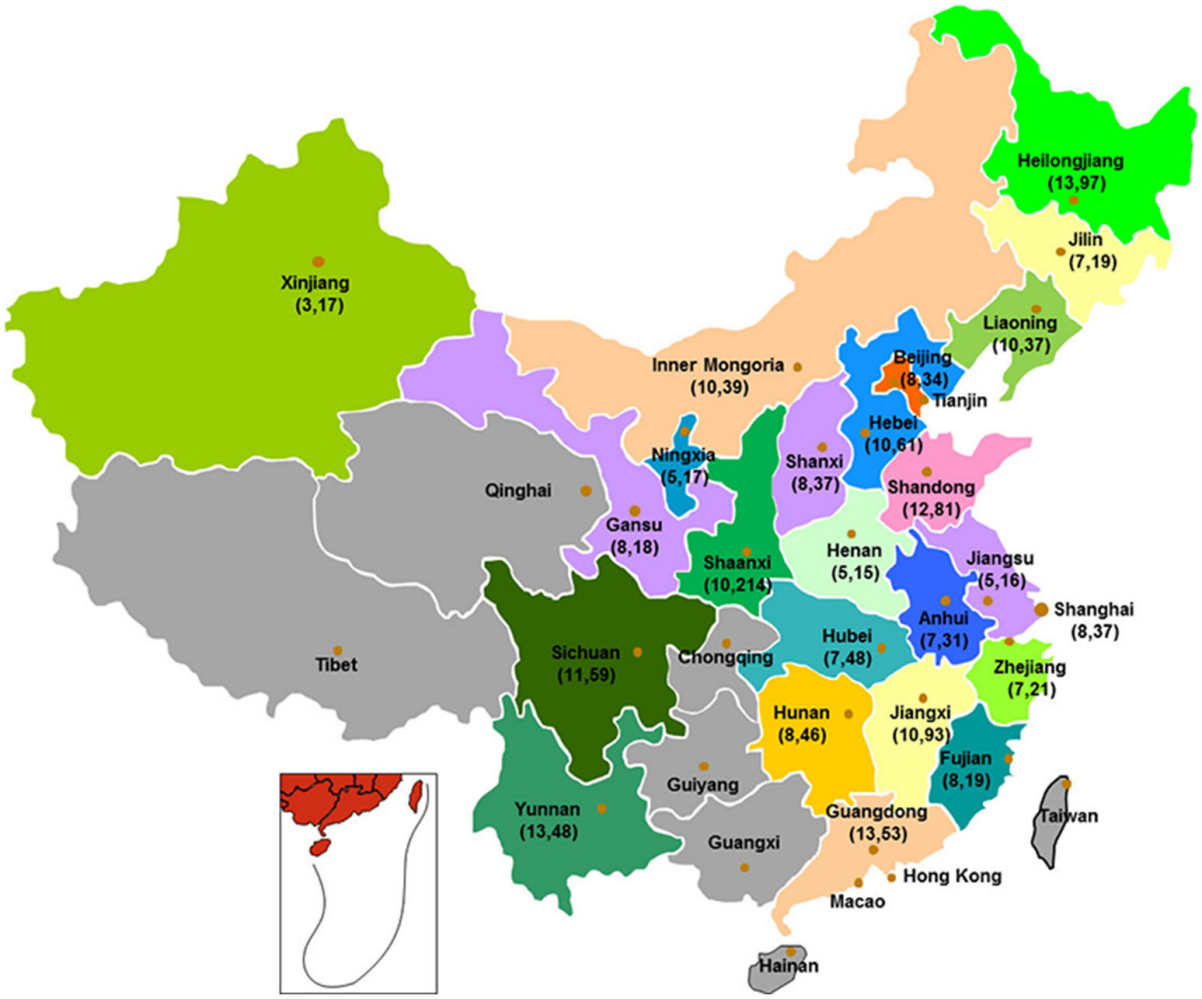
Map of China showing the location of the 24 provinces where the retail food samples were collected. The provinces marked in gray did not included in this study, while the other 24 provinces, where the retail food samples were collected, were marked colorfully. The numbers enclosed in parenthesis is the number of sampling cities (before the comma) and isolates (after the comma), respectively.

A total of 1,150 isolates were cultured from 27,000 retail food sampled in 2015 from retail markets from 203 cities of 24 provinces in China in 2015 in this study (Supplementary Table 1 and Figure 1). Samples included unpacked raw meat (5,000 livestock meat and 4,500 poultry meat), rice- and flour-products (3,000), vegetable salads (3,000), sandwich (3,000), meat and meat-products (2,500), eggs and egg-products (2,500), milk-products (2,000), condiments (500), bean-products (500), and fruit desserts (500). All samples were collected from supermarket outlets, including big departmental stores and local agriculture markets, street vendors. All samples collected were stored −80°C. The prevalence of *S. aureus* was determined using the qualitative detection method according to National Food Safety Standards of China document GB 4789.10-2010. Briefly, a 25 g sample was randomly collected from each sample and placed into a sterile glass flask (Xuzhou Yanjia Glass Products, Xuzhou, China) containing 225 mL of 10% saline solution (Land Bridge, Beijing, China). Following homogenization, the solutions were incubated at 37°C for 24 h. Loopfuls of the resulting cultures were streaked onto Baird-Parker Agar and Blood Agar (Land Bridge, Beijing, China), respectively, then incubated at 37°C for 24–48 h. Putative *S. aureus* isolates were tested for coagulase activity, and were further confirmed using API STAPH test strips (bioMerieux, Marcyl′Etoile, France).

For the coagulase test, presumptive *S. aureus* colonies were transferred into small tubes containing 0.2–0.3 mL BHI broth (Land Bridge, Beijing, China) and emulsify thoroughly, then incubated at 37°C for 18–24 h. A volume of 0.5 mL reconstituted coagulase plasma was mixed thoroughly with EDTA (Land Bridge, Beijing, China) and added into the BHI culture, then incubated at 35–37°C and examined periodically over 6 h period for clot formation. Only firm and complete clots that remained in place when the tube was tilted or inverted were considered positive for *S. aureus*. Coagulase positive *S. aureus* ATCC^TM^ 29213 and negative *S. epidermidis* ATCC^TM^ 12228 were tested simultaneously.

Finally, all isolates were subjected to PCR for detection of *16Sr RNA* and *nuc* (Louie et al., 2002). All confirmed *S. aureus* isolates were stored in brain heart infusion broth with 40% glycerol (Land Bridge, Beijing, China) at −80°C. Each sample retained one isolate at last.

### Antimicrobial susceptibility testing

Antimicrobial susceptibility of all *S. aureus* isolates against 13 antimicrobial agents was determined by the broth dilution method using the Biofosun® Gram-positive panel (Fosun Diagnostics, Shanghai, China) and interpreted according to the Clinical and Laboratory Standards Institute guidelines (CLSI) (CLSI, 2015). The panel of antimicrobial compounds tested included Penicillin (PEN) (0.06–8 μg/mL), Oxacillin (OXA) (0.25–16 μg/mL), Cefoxitin (CFX) (0.25–16 μg/mL), Vancomycin (VAN) (0.5–32 μg/mL), Daptomycin (DAP) (0.125–8 μg/mL), Erythromycin (ERY) (0.125–8 μg/mL), Gentamicin (GEN) (0.5–64 μg/mL), Tetracycline (TET) (0.5–32 μg/mL), Ciprofloxacin (CIP) (0.125–8 μg/mL), Clindamycin (CLI) (0.125–8 μg/mL), Trimethoprim-sulfamethoxazole (SXT) (0.125/2.3–8/152 μg/mL), Chloramphenicol (CHL) (1–64 μg/mL), and Linezolid (LNZ) (0.25–16 μg/mL). *S. aureus* ATCCTM 29213 was used as the control for the antimicrobial susceptibility testing.

### DNA purification

Frozen isolated were cultured overnight at 37°C in brain heart infusion broth. A TIANamp Bacterial DNA extraction kit (TianGen DNA Kit DP302, Beijing, China) was used to extract genomic DNA from the samples according to the manufacturer's instructions, which were adapted for Gram-positive bacteria through pretreatment with lysostaphin (0.1 g/L). A NanoDrop-2000 spectrophotometer (Thermo Fisher Scientific, NH, USA) was used to evaluate the quality of DNA. Samples diluted in sterile deionized water at a concentration of 50 mg/L were used as DNA templates for real-time PCR assay.

### Detection of *mecA* and enterotoxin genes

TaqMan PCR assays were run to test the presence of *mecA* gene and SEs genes. The primers and TaqMan probes of *mecA* and *sea* to *see* were synthesized by ThermoFisher Scientific (Waltham, MA, USA). Primers and TaqMan probes were designed for this study and are included in Table 1. Reaction mixtures (25 μL final volume) contained 1 × TaqMan Universal Master Mix II (no UNG) (Thermo Fisher Scientific, NH, USA); 10 pmol each primer; 6 μM TaqMan probe; and 1 μL template DNA. Amplification was carried out using a CFX96^TM^ Real-Time System (Bio-RAD, CA, USA) under the following parameters: 37°C for 4 min; 95°C for 10 min; 45 cycles of 95°C for 20 s and 60°C for 30 min. The positive control template DNA of MRSA ATCC^TM^ 33591 and *sea* to *see* (Longrunbio, Beijing, China) were applied in each PCR amplification reaction.

**Table 1:** Primers and probe used in this study.

**Primer/probe name**	**Sequence**	**PCR product size (bp)**	**References**
*sea*_FW	ATCAATTTATGGCTAGACGGTAAACA	94	Klotz et al., 2003
*sea*_RV	GAAGATCCAACTCCTGAACAGTTACA		
*sea*_Rrobe	ACAGTACCTTTGGAAACGGTTAAAACGAATAAGAAAA		
*seb*_FW	CGCATCAAACTGACAAACGAA	110	
*seb*_RV	ACCATCTTCAAATACCCGAACA		
*seb*_Probe	GGTGGTGTAACTGAGCATAATGGAAACCA		
*sec1*_FW	TTACACCCAACGTATTAGCAGAG	76	
*sec1*_RV	CCAGTGAATTTACTCGCTTTGTG		
*sec1*_Probe	CCAGACCCTACGCCAGATGAGTTG		
*sec2*_FW	AGACCCTACGCCAGATGA	106	
*sec2*_RV	CTACAGACATAACTTTAGTTGCTGATAC		
*sec2*_Probe	TCAAGTGAGTTTACTGGTACGATGGGT		
*sed*_FW	TTGATTCTTCTGATGGGTCTAAAGTCT	117	
*sed*_RV	GAAGGTGCTCTGTGGATAATGTTTT		
*sed*_Probe	TTATGATTTATTTGATGTTAAGGGTGATTTTCCCGA		
*see*_FW	AGATCTTCAGGCAAGGCATTAT	125	This study
*see*_RV	CATAACTTACCGTGGACCCTTC		
*see*_Probe	CTCAGACAGCTTTGGCGGTAAGGT		
*mecA*_FW	AAAGAACCTCTGCTCAACAAG	310	Zheng et al., 2015
*mecA*_RV	TGTTATTTAACCCAATCATTGCTGTT		
*mecA*_Probe	CCAGATTACAACTTCACCAGGTTCAACT		

### Determination of enterotoxin production

Enterotoxin production was determined using immuno-colloidal gold chromatographic test strips (Longrunbio, Beijing, China) for SEA to SEE specifically. Briefly, the supernatant of 24 h cultures of *S. aureus* (1 × 10^9^ CFU/mL) positive with enterotoxin genes grown at 37°C in a shake-tube (Xuzhou Yanjia Glass Products, Xuzhou, China) containing 5 mL Brain Hearth Infusion Broth (Land Bridge, Beijing, China)was separated from cells by centrifugation at 8,000 × g for 20 min. The supernatant was heated at 100°C for 10 min. Then 200 μL of the heated supernatant were tested for the presence of the SEs toxins using the strip test assay. A sample 100 ng/mL of SEA to SEE were used as a positive control and phosphate buffer was used as negative control. Additionally, in order to estimate the sensitivity and specificity of the strip tests, serial dilutions of the SEA to SEE toxins were also prepared.

### Statistical analysis

The Chi-square test was performed to compare the differences in proportion of isolates resistant to antimicrobial agents and positive with tested genes between sample types. Data analysis was performed using SPSS 20.0 (SPSS, Chicago, USA). All statistical tests were two-sided; *p* < 0.05 were considered statistically significant.

## Results

### Prevalence of *S. aureus* in retail food

Some 1,150 *S. aureus* isolates were recovered from 27,000 retail food (1,150/27,000, 4.3%) samples from various regions of China (Figure 1). Of these 1,150 isolates, 803 (9.4%, 803/9,500) were isolated from raw meat including 445 (9.9%, 445/4,500) poultry meat (302 chicken meat and 143 duck meat) and 358 (7.2%, 358/5,000) livestock meat (195 pork, 83 mutton and 80 beef); 90 (3.0%, 90/3,000) were isolated from rice- and flour-products; 85 (2.8%, 85/3,000) were isolated from vegetable salads; 69 (2.3%, 69/3,000) were isolated from sandwich; 46 (1.8%, 46/2,500) were isolated from meat and meat-products, mostly ready to eat food; 43 (1.7%, 43/2,500) were isolated from eggs and egg-products; and 14 were isolated from 6 (0.3%, 6/2,000) milk-products, 3 (0.6%, 3/500) condiments, 3 (0.6%, 3/500) fruit desserts, and 2 (0.4%, 2/500) bean-products (Table 2). Meanwhile, 69.4% (557/803) of the strains were isolated from farmer's market/street vendors, while 30.6% (245/803) of the strains were isolated from supermarket/departmental stores. Additionally, the prevalence rate of *S. aureus* among raw meat was higher than those among the other retail food (*p* < 0.01), and the prevalence rates of *S. aureus* among raw chicken meat and pork were higher than those among the other raw meat (*p* < 0.05). Raw chicken meat samples were the most contaminated with *S. aureus*.

**Table 2:** Prevalence of *S. aureus* in retail foods in China.

**Type of products**	**Samples tested no**.	**No. (%) Samples positive for *S.aureus***
Raw meat	9,500	803 (9.4)
Poultry meat	4,500	445 (9.9)
Chicken meat	2,500	302 (12.8)
Duck meat	2,000	143 (7.2)
Livestock meat	5,000	358 (7.2)
Pork	2,000	195 (9.8)
Mutton	1,500	83 (5.5)
Beef	1,500	80 (5.3)
Rice- and flour-products	3,000	90 (3.0)
Vegetable salads	3,000	85 (2.8)
Sandwich	3,000	69 (2.3)
Meat and meat-products	2,500	46 (1.8)
Eggs and egg-products	2,500	43 (1.7)
Milk-products	2,000	6 (0.3)
Condiments	500	3 (0.6)
Fruit desserts	500	3 (0.6)
Bean-products	500	2 (0.4)
Total	27,000	1150 (4.3)

### Susceptibility of *S. aureus* to antimicrobial compounds

Overall, 97.6% (1,122/1,150) of the *S. aureus* isolates exhibited resistance phenotypes to at least one antimicrobial agent (Table 3). The highest levels of resistance were observed for penicillin (83.7%, 963/1,150), followed by linezolid (67.7%, 778/1,150) and erythromycin (52.1%, 599/1,150), tetracycline (38.2%, 439/1,150), and clindamycin (31.0%, 356/1,150). Some isolates expressed resistance to trimethoprim-sulfamethoxazole, oxacillin, cefoxitin, chloramphenicol, gentamicin, and ciprofloxacin. All tested isolates were susceptible to vancomycin, and 93.8% (1,079/1,150) were susceptible to daptomycin (data not shown). Additionally, the isolates cultured from raw meats showed significantly higher resistance to tetracycline (42.8%), ciprofloxacin (17.4%), and chloramphenicol (12.0%) than those from eggs and egg-products (ciprofloxacin only), rice- and flour-products, vegetable salads, and sandwich (*p* < 0.05).

**Table 3:** Antimicrobial resistance of *S. aureus* isolated from retail food.

**Antimicrobial agents**	**No. of isolates resistant to the tested antimicrobial agents (%)**							
	**Total *N* = 1,150**	**Raw meat *N* = 803**	**Rice and flour products *N* = 90**	**Vegetable salads *N* = 85**	**Sandwich *N* = 69**	**Meat and its by products *N* = 46**	**Eggs and by products *N* = 43**	**Others *N* = 14**
Penicillin	83.7 (963)	84.1 (675)	83.3 (75)	80.0 (68)	82.6 (57)	84.8 (39)	90.7 (39)	71.4 (10)
Oxacillin	9.0 (103)	8.7 (70)	15.6 (14)	1.2 (1)	10.1 (7)	10.9 (5)	9.3 (4)	14.3 (2)
Cefoxitin	9.7 (111)	9.6 (77)	15.6 (14)	4.7 (4)	8.7 (6)	6.5 (3)	14.0 (6)	7.1 (1)
Vancomycin	0.0 (0)	0.0 (0)	0.0 (0)	0.0 (0)	0.0 (0)	0.0 (0)	0.0 (0)	0.0 (0)
Daptomycin	0.0 (0)	0.0 (0)	0.0 (0)	0.0 (0)	0.0 (0)	0.0 (0)	0.0 (0)	0.0 (0)
Erythromycin	52.1 (599)	55.0 (442)	44.4 (40)	37.7 (32)	43.5 (30)	52.2 (24)	48.8 (21)	71.4 (10)
Gentamicin	13.3 (153)	15.1 (121)	10.0 (9)	9.4 (8)	4.4 (3)	13.0 (6)	11.6 (5)	7.1 (1)
Tetracycline	38.2 (439)	42.8 (344)	27.8 (25)	25.9 (22)	23.2 (16)	32.6 (15)	32.6 (14)	21.4 (3)
Ciprofloxacin	13.6 (156)	17.4 (140)	5.6 (5)	4.7 (4)	2.9 (2)	6.5 (3)	2.3 (1)	0.0 (0)
Clindamycin	31.0 (356)	33.4 (268)	26.7 (24)	20.0 (17)	24.6 (17)	26.1 (12)	25.6 (11)	57.1 (8)
Trimethoprim-sulfamethoxazole	6.1 (70)	6.4 (51)	5.6 (5)	4.7 (4)	1.5 (1)	8.7 (4)	4.7 (2)	21.4 (3)
Chloramphenicol	9.9 (114)	12.0 (96)	4.4 (4)	4.7 (4)	4.4 (3)	6.5 (3)	7.0 (3)	0.0 (0)
Linezolid	67.7 (778)	68.6 (551)	71.1 (64)	64.7 (55)	55.1 (38)	76.1 (35)	55.8 (24)	78.6 (11)
Pansusceptible	2.4 (28)	1.5 (12)	4.4 (4)	7.1 (6)	0.0 (0)	10.9 (5)	2.3 (1)	0.0 (0)
≥ 1 antimicrobial	97.6 (1,122)	98.5 (791)	95.6 (86)	92.9 (79)	100.0 (69)	89.1 (41)	97.7 (42)	100.0 (14)
≥ 3 class of antimicrobials	57.5 (661)	62.4 (501)	46.7 (42)	48.2 (41)	42.0 (29)	39.1 (18)	48.8 (21)	64.3 (9)
≥ 8 class of antimicrobials	2.4 (28)	3.0 (26)	1.1 (1)	0.0 (0)	1.4 (1)	0.0 (0)	0.0 (0)	0.0 (0)

*N, total number of S. aureus isolates tested for susceptibility in different retail food*.

**Figure 2:**
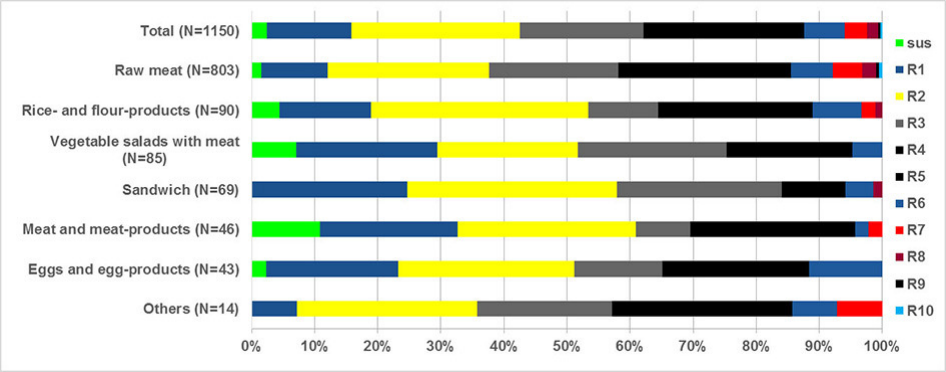
Frequency distribution of *S. aureus* isolated from retail food completely susceptible or resistant to 1 to 10 antimicrobial classes. *n*, total number of *S. aureus* isolates tested for susceptibility in different retail food; sus, susceptible to all antimicrobial classes; R1–R10, resistance to 1 up to 10 antimicrobial classes.

Among the 1,150 *S. aureus* isolates, 661 isolates (57.5%) were resistant to three or more classes of antimicrobials (MDR). Twenty-eight isolates were resistant to eight or more classes of antimicrobials, among them, most were isolated from raw meats (26/28, 92.8%). Four isolates from raw meats were found to be resistant to 10 classes of antimicrobial agents being susceptible only to daptomycin and vancomycin. Notably, the isolates cultured from raw meats exhibited substantially higher MDR (62.4%) than those from rice- and flour-products (46.7%), vegetable salads (48.2%), sandwich (42.0%), and meat and meat-products (39.1%) (*p* < 0.05). The detail antimicrobial resistance profiles have been shown in Supplementary Table 2 and Figure 2.

### Presence of *mecA* and enterotoxin genes among *S. aureus* isolates

Of the 1,150 *S. aureus* isolates, 91 isolates (7.9%, 91/1,150) were identified as MRSA by PCR (Table 4). In detail, 7.2% (58/803) of raw meats, 10% (9/90) of rice- and flour-products, 1.2% (1/85) of vegetable salads, 7.2% (5/69) of sandwiches, 6.5% (3/46) of meat and meat-products, 32.6% (14/43) of eggs and egg-products, were positive with *mecA*, respectively (Table 4). Meanwhile, the enterotoxin- encoding genes were also amplified. Results showed that *sea, seb, sec*, and *sed* genes were detected in 11.5% (132), 15.1% (174), 10.1% (116), 7.5% (86) of 1,150 *S. aureus* including all types of food samples in this study (Table 4). In all 29.7% (341/1,150) of *S. aureus* harbored the targeted classical SEs genes (*sea, seb, sec*, and *sed*). While, no *see* genes were found in this study. Sixteen different patterns were observed (Table 5). The most prevalent gene pattern was the *seb* (10.4%, 120/1,150) followed by *sea* (9%, 103/1,150) genotypes. In addition, 11 gene patterns contained two or three genes, including *sea*-*seb* (1.1%, 13/1,150), *sea*-*sec* (0.8%, 9/1,150), *sea*-*mecA* (0.2%, 2/1,150), *seb*-*sec* (0.2%, 2/1,150), *seb*-*mecA* (3%, 34/1,150), *sec*-*sed* (3.7%, 42/1,150), *sec*-*mecA* (0.1%, 1/1,150), *sed*-*mecA* (0.3%, 4/1,150) *sea*-*seb*-*sec* (0.3%, 3/1,150), *sea*-*seb*-*mecA* (0.2%, 2/1,150), and *sec*-*sed*-*mecA* (0.1%, 1/1,150). A total of 44 out of 91 MRSA isolates (48.4%, 44/91) were harbored one or two SEs genes.

**Table 4:** Prevalence of *mecA* and enterotoxin genes and the presence of the produced enterotoxins in *S. aureus* isolated from retail food.

**Study genes**	**Percentage of** ***S. aureus*** **isolates positive for the detected genes %(*****n*****)**							
	**Total *N* = 1,150**	**Raw meat *N* = 803**	**Rice and flour product *N* = 90**	**Vegetable salads *N* = 85**	**Sandwich *N* = 69**	**Meat and meat product *N* = 46**	**Eggs and egg product *N* = 43**	**Others *N* = 14**
*Sea*	11.5 (132)	11.3 (91)	13.3 (12)	12.9 (11)	10.1 (7)	13 (6)	11.6 (5)	–
*Seb*	15.1 (174)	14.4 (116)	18.9 (17)	10.6 (9)	18.8 (13)	10.9 (5)	30.2 (13)	7.1 (1)
*Sec*	10.1 (116)	9.2 (74)	6.7 (6)	11.8 (10)	15.9 (11)	23.9 (11)	4.7 (2)	14.3 (2)
*Sed*	7.5 (86)	6.7 (54)	6.7 (6)	8.2 (7)	8.7 (6)	19.6 (9)	9.3 (4)	–
*mecA*	7.9 (91)	7.2 (58)	10 (9)	1.2 (1)	7.2 (5)	6.5 (3)	32.6 (14)	7.1 (1)
Toxins production	**c/n%, (c)**	**c/n%, (c)**	**c/n%, (c)**	**c/n%, (c)**	**c/n%, (c)**	**c/n%, (c)**	**c/n%, (c)**	**c/n%, (c)**
SEA	90.9 (120)	91.2 (83)	91.7 (11)	90.9 (10)	6	5	4	1
SEB	94.3 (164)	93.1 (108)	94.1 (16)	9/	92.3(12)	5	100 (13)	1
SEC	5.2 (6)	4.1 (3)	0	10 (1)	1	9.1 (1)	0	0
SED	80.2 (69)	79.6 (43)	5	6	4	9	2	0

*N, total number of S. aureus isolates in this study; n, number of S. aureus isolates positive for this gene; c, number of S. aureus isolates showed toxigenic capabilities by strips; c/n%, percentage of S. aureus isolates showed toxigenic capabilities of S. aureus isolates positive for this toxin gene; –, no isolates positive for this study gene*.

**Table 5:** Gene patterns in *S. aureus* isolated from retail food.

**Gene patterns**	**Percentage of** ***S. aureus*** **isolates positive for the gene profiles % (*****n*****)**							
	**Total *N* = 1,150**	**Raw meat *N* = 803**	**Rice and flour products *N* = 90**	**Vegetable salads *N* = 85**	**Sandwich *N* = 69**	**Meat and meat-product *N* = 46**	**Eggs and egg-products *N* = 43**	**Others *N* = 14**
*Sea*	9 (103)	9.1 (73)	11.1 (10)	9.4 (8)	2.9 (2)	10.9 (5)	11.6 (5)	
*Seb*	10.4 (120)	10.5 (84)	10 (9)	8.2 (7)	10.1 (7)	6.5 (3)	23.3 (10)	–
*Sec*	5 (58)	4.5 (36)	3.3 (3)	8.2 (7)	7.2 (5)	8.7 (4)	4.7 (2)	7.1 (1)
*Sed*	3.4 (39)	2.6 (21)	4.4 (4)	5.9 (5)	7.2 (5)	4.3 (2)	4.7 (2)	–
*mecA*	4.1 (47)	3.9 (31)	4.4 (4)	–	–	4.3 (2)	20.9 (9)	7.1 (1)
*sea-seb*	1.1 (13)	1 (8)	2.2 (2)	1.2 (1)	1.4 (1)	2.2 (1)	–	–
*sea-sec*	0.8 (9)	0.6 (5)	–	1.2 (1)	4.3 (3)	–	–	–
*sea-mecA*	0.2 (2)	0.2 (2)	–	–	–	–	–	–
*seb-sec*	0.2 (2)	–	1.1 (1)	–	–	–	–	7.1 (1)
*seb-mecA*	3 (34)	2.6 (21)	5.6 (5)	–	5.8 (4)	2.2 (1)	7 (3)	–
*sec-sed*	3.7 (42)	3.7 (30)	2.2 (2)	2.4 (2)	1.4 (1)	15.2 (7)	–	–
*sec-mecA*	0.1 (1)	–	–	–	1.4 (1)	–	–	–
*sed-mecA*	0.3 (4)	0.2 (2)	–	–	–	–	4.7 (2)	–
*sea-seb-sec*	0.3 (3)	0.2 (2)	–	–	1.4 (1)	–	–	–
*sea-seb-mecA*	0.2 (2)	0.1 (1)	–	1.2 (1)	–	–	–	–
*sec-sed-mecA*	0.1 (1)	0.1 (1)	–	–	–	–	–	–

*N, total number of S. aureus isolates in this study; n, the number of S. aureus isolates positive for this gene profile; –, no isolates positive for this gene profile*.

### Determination of enterotoxin production

**Figure 3:**
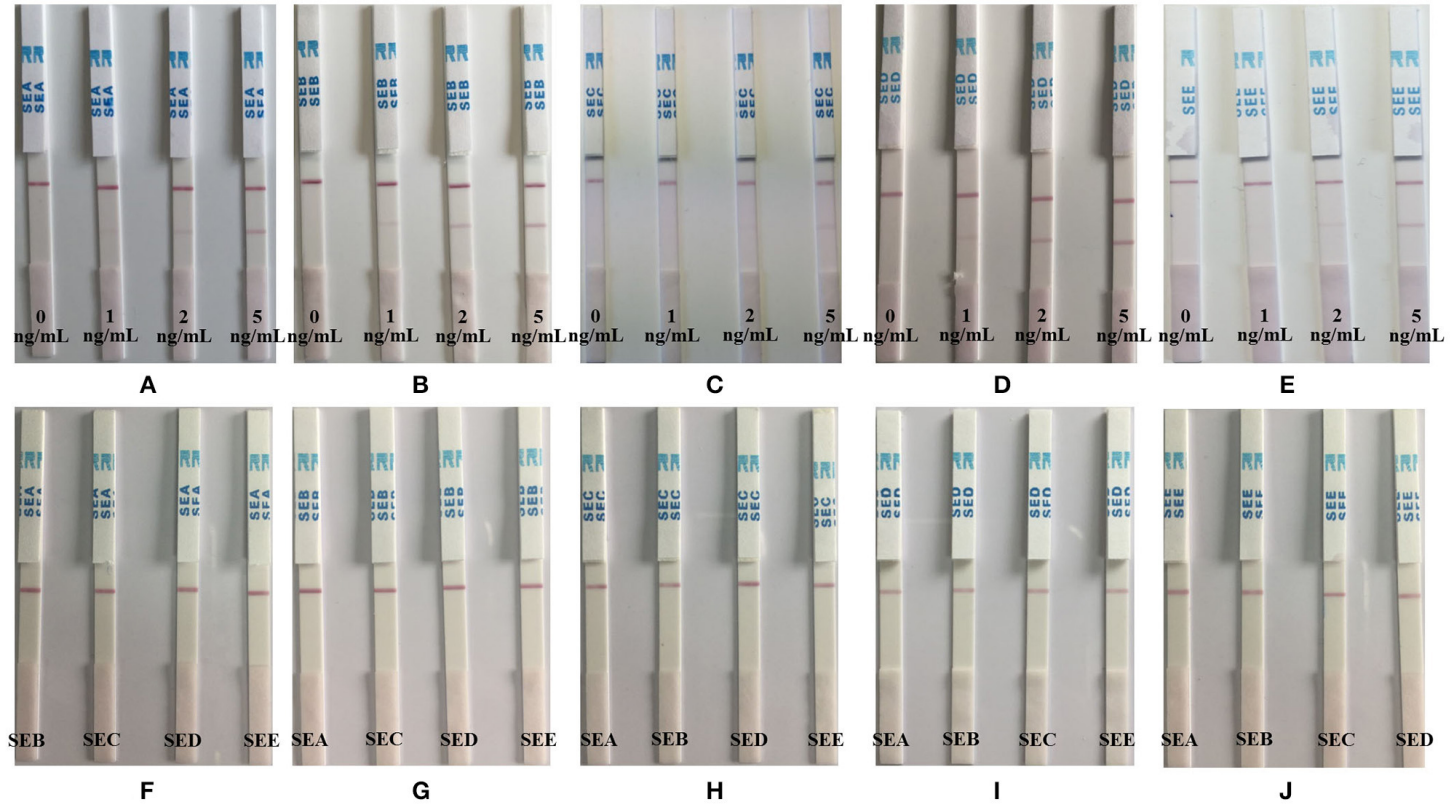
Sensitivity and specificity of the immuno-colloidal gold chromatographic test strips assay for the detection of SEA to SEE. Series of dilutions (0–5 ng/mL) of SEA to SEE **(A–E)** were prepared in phosphate buffer that detected by the test strips for SEA to SEE, respectively. 100 ng/mL of SEA to SEE **(F–J)** were prepared in phosphate buffer for the specificity detection of the test strips.

In this study, the sensitivity and specificity of the immuno-colloidal gold chromatographic test strips, was tested. Series of dilutions (0–5 ng/mL) of SEA to SEE (Figures 3A–E) were prepared in phosphate buffer designed to detect the test strips for SEA to SEE, respectively. Some 100 ng/mL of SEA to SEE (Figures 3F–J) were prepared in phosphate buffer for the specificity detection element of the test strips (Figure 3). The intensity of the red color on the test line was proportional to the SE concentration and the control line should be visualized at all times. The detection limits of SEA, SEB, and SED were 1 ng/mL and the detection limits of SEC and SEE were 2 ng/mL, while the specificities of all SEs were 100%.

A total of 341 isolates were positive by PCR for toxin genes and when tested for toxigenic capabilities, 120, 164, 6, and 69 isolates were positive for SEA, SEB, SEC, and SED, respectively. The results showed that more than 80% of the enterotoxigenic *S. aureus* isolates produced enterotoxins SEA, SEB, and SED, with the exception that only 6 out of 116 *sec* gene positive with *S. aureus* isolates produced enterotoxin SEC, as detected by immuno-colloidal gold chromatographic test strips (Table 4). Additionally, 16 isolates were simultaneously produced two or three types of enterotoxins. Moreover, 38 MRSA isolates also produced enterotoxin SEB, while 1 MRSA isolate produced both SEA and SEB Enterotoxins.

## Discussion

Earlier studies reported on the prevalence of *S. aureus* and MDR *S. aureus* among retail food samples in P. R. China. However, no national level comprehensive epidemiological data are available describing the prevalence of *S. aureus* and MDR *S. aureus* among different retail food samples in P. R. China. To the best of our knowledge, the current study is the first to report the epidemiological prevalence and distribution of *S. aureus* and MDR *S. aureus* among retail food samples in P. R. China. Samples screened in this study were obtained from different retail food samples from different provinces of China. Therefore, the results of this study provide estimations of the prevalence of *S. aureus* and MDR *S. aureus* in the different retail food samples in China. Moreover, the MRSA (*mecA*), enterotoxin genes and enterotoxin production were also characterized among *S. aureus* isolates.

In this study, the prevalence of *S. aureus* in food samples was 4.3% (1,150/27,000), which is similar to that observed (3.2%, 163/5,142) in retail foods (cooked food and vegetables) in China (Tang et al., 2016), but lower than the documented rates reported in Shanghai (19.3%, 117/607) or Shaanxi (22.1%, 438/1,979) province in China (Wang et al., 2014; Song et al., 2015). As mentioned above, isolates screened in this study were obtained from 203 cities cross different provinces of China. Therefore, these data is a more comprehensive, systematic, and representative of China as a whole.

It has already been reported that the raw meats contaminated with *S. aureus* is a major cause of food poisoning in the world, particularly China (Waters et al., 2011; Wang et al., 2014). The risk of infectious will increase when food contaminated with *S. aureus* is not cooked sufficiently or some ready-to-eat food is contaminated with this bacterium by cross contamination. Moreover, SEs will be produced and accumulate when this raw meat is stored at inappropriate temperature. In this study raw meat especially raw chicken meat and pork were the most contaminated food types. The prevalence rates of raw chicken meat and pork were 12.8% and 9.8%, respectively, which were similar to other Chinese reports (Wang et al., 2014; Song et al., 2015; Tang et al., 2016). Chicken and pigs could be contaminated by *S. aureus* during breeding, slaughtered, and consequently their meat would be contaminated by *S. aureus* during sale, transportation, and family store and cooking (You et al., 2016; Kraushaar et al., 2017; Wijesurendra et al., 2017). In this study all raw meat samples were unpacked and collected from marketing places. Although these meats were permitted sale by the Food Hygiene Bureau at the beginning, unpacked treatment may later increase the probability of the contamination of *S. aureus* during their shelf-life especially that nearly 70% of the isolates were cultured from farmer's market/street vendors, where there may not good management oversight of food hygiene as in the case of supermarket/department stores. It is notably that chicken and pork are the main source of animal protein in China. Therefore, controls in breeding and at the slaughter stage to prevent and control the contamination of *S. aureus*, are required.

Over the last few decades, *S. aureus* strains with antimicrobial resistance have been frequently reported in foods, leading to substantial financial and economic losses (Richter et al., 2012). In this study, 97.6% (1,122/1,150) *S. aureus* isolates were resistant to at least one antimicrobial. The substantially higher resistance was found against penicillin, erythromycin and tetracycline. These observations are in agreement with the data reported in China and other countries (Lv et al., 2014; Wang et al., 2014; Kroning et al., 2016; Ge et al., 2017; Kraushaar et al., 2017). A possible reason for this high level of resistance to antimicrobial agents could be due to the extensive use of such compound for the treatment of animal infections (Cui et al., 2009; Liu et al., 2009; Chu et al., 2013). Additionally, a total of 2.4% (661 of 27,000) retail foods isolates were found positive with MDR *S. aureus* in this study. These observations are similar to those reported in study in USA, in which 2.9% (103/3,520) were identified with MDR *S. aureus* (Ge et al., 2017).

In this study, the resistance rates of *S. aureus* isolates cultured from raw meats were significantly higher for tetracycline, ciprofloxacin and chloramphenicol than those isolated from other retail foods items such eggs and egg-products, rice- and flour-products, vegetable salads, and sandwich. The prevalence rates of MDR *S. aureus* were also significantly higher among isolates cultured from raw meats than those isolated from other foods (rice- and flour-products, vegetable salads, sandwich, meat and meat-products) in this study. The same high contamination rate of *S. aureus* among retail meat has also been reported in China and other countries including Denmark, England, USA, Switzerland, Saudi Arabia (Wang et al., 2014; Raji et al., 2016; Zogg et al., 2016; Ge et al., 2017; Tang et al., 2017). Thus, it is very important to develop strategies to eliminate or decrease the prevalence of *S. aureus* in foods especially in raw meat.

MRSA is a potential cause of hospital-acquired infection. However, community-acquired infections are also increasing persistently (Cho and Chung, 2017; Gopal and Divya, 2017). It has already been suggested that foods contaminated pathogens might be a potential cause of community-acquired MRSA (Jones et al., 2002). In recent years, MRSA strains have been identified in various foods including milk, RTE foods, and meat products (Hong et al., 2015; Chang et al., 2016; Jans et al., 2017; Osman et al., 2017; Vojkovská et al., 2017). In this study, the prevalence rate of *mecA* gene (MRSA) was 0.3% (91/27,000). Current study MRSA prevalence rate is lower compared with previously reported data in China or other countries (1.7–9.6%) (Weese et al., 2010; Wang et al., 2014; Song et al., 2015). Although, MRSA prevalence in retail foods is relatively low in this study, the risk of its transmission through the food chain, especially by uncooked food, along with the spread of MDR strains, cannot be ignored. The emergence of food-borne out break caused by MRSA has already been reported (Jones et al., 2002; Lv et al., 2014). On the basis of these observations, we suggest that attention should be paid by governments and individuals to prevent the further spread of MRSA.

Current, new types of SEs (SEG-SElV and some SE-like super antigens) have been reported (Kroning et al., 2016; Puah et al., 2016). However, the role of these new types of SEs is still unclear in food poisoning (Sasaki et al., 2012). It is reported that about 95% of staphylococcal food poisoning (SFP) are associated with the classical SEs (SEA to SEE) (Kokan and Bergdoll, 1987). Thus, we targeted five classical SEs to screen SE-producing *S. aureus* in this study and found that 341 *S. aureus* isolates (29.7%, 341/1,150) harbored at least one of the SEs genes in this study, which is similar to those reported in Malaysia (30.8%) and the USA (25.8%) (Puah et al., 2016; Ge et al., 2017). The *sea* and *seb* genes were the major two frequent SEs genes among the tested isolates in this study with the percentage of 11.5 and 15.1%, respectively, followed by *sec* (10.1%) and *sed* (7.5%), with no *see* gene found in any of the isolates tested. These finding are consistent with other reports in China and several other countries (Wang et al., 2014; Song et al., 2015; Puah et al., 2016; Ge et al., 2017). But a study report by Zhang et al found that *sea* and *sed* genes were the most prevalent SEs genes among *S. aureus* isolates among retail foods in China. Of note, the *sea* gene is the most commonly reported in contaminated foods and also in staphylococcus-related food poisoning cases worldwide while *seb* gene could cause more severe poisoning than other enterotoxins (Argudín et al., 2010; Gholamzad et al., 2015).

In this study, more than 90% of the *sea* and *seb* genes carrying *S. aureus* isolates and 80% of the *sed* gene carrying *S. aureus* isolates has produced enterotoxins when tested with specific immuno-colloidal gold chromatographic test strips, respectively. Only 5.2% of the *sec* gene carrying *S. aureus* isolates produced SEC when tested with the test strips for SEC. One possible interpretation of this pattern is that the anti-SEC antibody was produced based on SEC1, while the primers and probe used in this study were designed to detect SEC1 and SEC2 encoding genes. Therefore, we suggest that the test strips should to be improved to meet the need of all subtypes of SEC detection in the future. Additionally, 22% (72/341) of the SEs genes carrying *S. aureus* isolates harbored two or three SEs genes, and 16 isolates has produced two or three types of enterotoxins. Additionally, almost 50% of the MRSA isolates were in combination with at least one SEs gene in this study. All SEs genes were detected among food-derived MRSA isolates in this has already been reported in previous studies (Rhee and Woo, 2010; Fessler et al., 2011; Vestergaard et al., 2012; Wang et al., 2014). In this study, 97.6% isolates were resistant to at least one and 57.5% were resistant to at least three antimicrobials. The consumption of food contaminated with these enterotoxigenic particularly MRSA isolates could pose a serious public health risk. Thus, there is a need to monitor the antimicrobial susceptibility and enterotoxigenicity of MDR *S. aureus* and MRSA isolated from retail foods and develop strategies to prevent and control the contamination of this bacterium in food.

Considering the potential that these MDR *S. aureus* and MRSA occurring in retail food especially RTE food maybe transferred to the consumer, and these strains may enter the food chain (van Loo et al., 2007), it is important to monitor the antimicrobial susceptibility and mechanisms of resistance of this bacterium along the whole food chain. Moreover, it is imperative that National governments develop strong and effective legislation to regulate the use of antimicrobial compounds in food-producing animals, along with standards to limit residues in the food chain. Food industries should comply with these standards as a means of controlling these antimicrobial resistant pathogens.

## Conclusions

This study provides detail epidemiological estimations of prevalence of *S. aureus* and MRSA in retail foods in China. Our study found a relatively low prevalence of *S. aureus* but the high prevalence rates of MDR *S. aureus* and enterotoxigenic *S. aureus* could cause severe outbreaks. Additionally, our study also demonstrated that most *S. aureus* were found to be resistant to commonly used antimicrobial agents which raised concerns regarding transmission risk following the consumption of food contaminated with these bacteria. Our study highlights the importance of monitoring the antimicrobial susceptibility and enterotoxigenicity of MDR *S. aureus* and MRSA in the food chains including retail foods, food producing animals, and even human beings, and these data could be used proactively to assist government and industries in China to develop improved food safety measures, designed to reduce the contamination and transmission of this bacterium. Additionally, a future large-scale, multi-population-based study must be conducted to obtain more comprehensive data on the prevalence and distribution of *S. aureus* in various Chinese ethnic populations.

## Availability of data and materials

The aggregate data supporting findings contained within this manuscript will be shared upon request submitted to the corresponding author.

## Author contributions

WW, ZB, FL, AM, and JX designed experiments; TJ, CZ, and ZP carried out experiments; ZB, WW, JX, and SF analyzed experimental results. ZB, WW, AM, and JX wrote the manuscript.

### Conflict of interest statement

The authors declare that the research was conducted in the absence of any commercial or financial relationships that could be construed as a potential conflict of interest.
