# Supplementary material for: Enterotoxigenicity and Antimicrobial Resistance of Staphylococcus aureus Isolated from Retail Food in China
Source: Front Microbiol. 2017 Nov 21;8:2256. doi: 10.3389/fmicb.2017.02256 (PMC5702451; doi:10.3389/fmicb.2017.02256)
Supplement: Supplementary file 1 [file Table1.docx]

**Supplemental Table 1Sampling information of *S. aureus* isolates in this study**

| **no.** | **Food classes** | **Food names** | **Packing type** | **sampling site** | **Amount** | **Date of Sampling** | **Sampling provinces** | **Sampling town** | **Sampling locations** | **Manufacturers** | **Date of manufacture** |
| --- | --- | --- | --- | --- | --- | --- | --- | --- | --- | --- | --- |
| SA001 | Raw meat | Mutton | unpacked | Supermarket/department stores | >500g | 2015-04-27 | Anhui | Chizhou city | Changjiang road, RT-Mart supermarket in chizhou city 2 Floor food area | Shanghai Phoenix foods limited | 2015.01.05 |
| SA002 | Raw meat | Mutton | unpacked | Supermarket/department stores | >500g | 2015-04-27 | Anhui | Chizhou city | Changjiang Lu Yonghui supermarket food in chizhou city | Shanghai da-Mu Khan Foods Ltd | 2014.10.22 |
| SA003 | Raw meat | Pork | unpacked | Supermarket/department stores | >500g | 2015-05-18 | Anhui | Chizhou city | Changjiang road, RT-Mart supermarket in chizhou city 2 Floor food area | Suzhou food co | 2015.05.18 |
| SA004 | Raw meat | Pork | unpacked | Supermarket/department stores | >500g | 2015-05-18 | Anhui | Chizhou city | Changjiang Lu Yonghui supermarket food in chizhou city | Suzhou food co | 2015.05.18 |
| SA005 | Raw meat | Pork | unpacked | Supermarket/department stores | >500g | 2015-04-27 | Anhui | Chizhou city | Changjiang road, RT-Mart supermarket in chizhou city 2 Floor food area | Suzhou food co | 2015.04.27 |
| SA006 | Raw meat | Duck | unpacked | Supermarket/department stores | >500g | 2015-07-13 | Anhui | Huainan city | National road 6 RT-Mart supermarket | Unknown | 20151012 |
| SA007 | Raw meat | Chicken | unpacked | farmer’s market/street vendors | >500g | 2015-05-11 | Anhui | Huainan city | Liu Jia in the mountain base to the market 20 M | Liu Yang 18055443274 | 20150511 |
| SA008 | Raw meat | Duck | unpacked | farmer’s market/street vendors | >500g | 2015-05-11 | Anhui | Huainan city | Shungeng da Hua West Road farmers ' market 15 M | Pang Shiwu 13695543126 | 20150511 |
| SA009 | Raw meat | Chicken | unpacked | farmer’s market/street vendors | >500g | 2015-10-12 | Anhui | Huainan city | Turntable Street farmers ' market | Unknown | 20151012 |
| SA010 | Raw meat | Chicken | unpacked | farmer’s market/street vendors | >500g | 2015-10-12 | Anhui | Huainan city | Cave Hill Road brothers restaurant | Unknown | 20151012 |
| SA011 | Raw meat | Duck | unpacked | farmer’s market/street vendors | >500g | 2015-05-11 | Anhui | Huainan city | Liu Jia in the mountain base to the market 20 M | Liu Yang 18055443274 | 20150511 |
| SA012 | Raw meat | Mutton | unpacked | farmer’s market/street vendors | >500g | 2015-09-14 | Anhui | Ma On Shan city | Ma Ma Xiaoyun village farmers ' markets stalls | No | 2015.09.14 |
| SA013 | Raw meat | Pork | unpacked | farmer’s market/street vendors | >500g | 2015-08-17 | Anhui | Wuhu city | Wan Zhi Zhen, Wuhu City South of market 2-106 Booth | Ominous | 20151012 |
| SA014 | Raw meat | Duck | unpacked | Supermarket/department stores | >500g | 2015-09-07 | Anhui | Xuancheng city | Peach State Rd Ke long supermarket in Guangde County 0563-6038883 | Shandong linyi Lu Cheng food co | 20150823 |
| SA015 | Raw meat | Pork | unpacked | Supermarket/department stores | >500g | 2015-05-15 | Shanxi | Changzhi city | 588 Supermarket | Xin sheng, Gaoping meat co | 2015.5.11 |
| SA016 | Raw meat | Mutton | unpacked | Supermarket/department stores | >500g | 2015-05-15 | Shanxi | Changzhi city | Supermarket | Shanxi Changzhi West Street butcher's shop | 2015.5.12 |
| SA017 | Raw meat | Pork | unpacked | farmer’s market/street vendors | >500g | 2015-05-15 | Shanxi | Changzhi city | Gulou Street butcher's shop | Gulou Street, licheng into a butcher's shop | 2015.5.13 |
| SA018 | Raw meat | Beef | unpacked | Supermarket/department stores | >500g | 2015-10-09 | Shanxi | In Jincheng city | Gaoping city, Shan XI Nan lu, red square | Unknown | 20151009 |
| SA019 | Raw meat | Beef | unpacked | farmer’s market/street vendors | >500g | 2015-10-10 | Shanxi | In Jincheng city | Yangcheng County Nong market halal meat shop | Unknown | 20151010 |
| SA020 | Raw meat | Beef | unpacked | farmer’s market/street vendors | >500g | 2015-10-14 | Shanxi | In Jincheng city | Qinshui Wavelet apricot market butcher shop | Unknown | 20151014 |
| SA021 | Raw meat | Pork | unpacked | farmer’s market/street vendors | >500g | 2015-10-15 | Shanxi | In Jincheng city | Chengqu district of Jincheng city amphibious homes market for the meat shop | Unknown | 20151015 |
| SA022 | Raw meat | Chicken | unpacked | farmer’s market/street vendors | >500g | 2015-08-19 | Shanxi | In Jincheng city | BA Gong Zhen, zezhou County Nong Mao Shi Chang live chicken stalls | Unknown | 20150819 |
| SA023 | Raw meat | Chicken | unpacked | farmer’s market/street vendors | >500g | 2015-08-19 | Shanxi | In Jincheng city | BA Gong Zhen, zezhou County Nong Mao Shi Chang live chicken stalls | Unknown | 20150819 |
| SA024 | Raw meat | Chicken | unpacked | Supermarket/department stores | >500g | 2015-10-13 | Shanxi | In Jincheng city | Recovery road, lingchuan County 10 On-site shopping mall in Hong Kong | Unknown | 20151013 |
| SA025 | Raw meat | Chicken | unpacked | Supermarket/department stores | >500g | 2015-04-22 | Shanxi | In jinzhong city | Noble, Chaoyang Street Times Square Shopping Center | Noble, Chaoyang Street, shouyang Times Square Shopping Center | 20150422 |
| SA026 | Raw meat | Pork | unpacked | farmer’s market/street vendors | >500g | 2015-05-26 | Shanxi | In jinzhong city | Farmers ' markets old close the pork shop | Farmers ' markets old close the pork shop | 15-5-26 |
| SA027 | Raw meat | Chicken | unpacked | Supermarket/department stores | >500g | 2015-06-23 | Shanxi | In jinzhong city | Noble times square every good supermarket | Noble times square every good supermarket | 15-6-23 |
| SA028 | Raw meat | Chicken | unpacked | farmer’s market/street vendors | >500g | 2015-06-24 | Shanxi | In jinzhong city | Convenience and a street market | Convenience and a street market | 15-6-24 |
| SA029 | Raw meat | Duck | unpacked | farmer’s market/street vendors | >500g | 2015-06-24 | Shanxi | In jinzhong city | Convenience and a street market | Convenience and a street market | 15-6-24 |
| SA030 | Raw meat | Duck | unpacked | Supermarket/department stores | >500g | 2015-06-25 | Shanxi | In jinzhong city | Xin Hua Street House Lee supermarket Century Plaza store | Shandong liuhe group Xiaoyi elephant farm foods limited | 15-5-9 |
| SA031 | Raw meat | Chicken | unpacked | Supermarket/department stores | >500g | 2015-06-26 | Shanxi | In jinzhong city | Liu Gen Dong Jie 1 Great supermarket, beauty | Liu Gen Dong Jie 1 Great supermarket, beauty | 15-6-26 |
| SA032 | Raw meat | Duck | unpacked | Supermarket/department stores | >500g | 2015-06-26 | Shanxi | In jinzhong city | Liu Gen Dong Jie 1 Great supermarket, beauty | Double Dragon in Changzhi city foods limited | 15-1-20 |
| SA033 | Raw meat | Chicken | unpacked | Supermarket/department stores | >500g | 2015-06-26 | Shanxi | In jinzhong city | Sen supermarket peace store | Sen supermarket peace store | 15-6-26 |
| SA034 | Raw meat | Pork | unpacked | Supermarket/department stores | >500g | 2015-10-27 | Shanxi | Taiyuan | Special good Riverside road shops | Elm City, animal husbandry development co, Ltd | 20151025 |
| SA035 | Raw meat | Chicken | unpacked | farmer’s market/street vendors | >500g | 2015-10-27 | Shanxi | Taiyuan | Camp market soy products shop | Elephant birds industry co | 20151023 |
| SA036 | Raw meat | Pork | unpacked | Supermarket/department stores | >500g | 2015-10-08 | Shanxi | In Xinzhou city | A surname Street supermarket | Xinzhou city, Shanxi province, unknown | 10151008 |
| SA037 | Raw meat | Mutton | unpacked | farmer’s market/street vendors | >500g | 2015-10-07 | Shanxi | In Xinzhou city | Writing town farmers ' market | Xinzhou city, Shanxi province, unknown | 20151007 |
| SA038 | Raw meat | Chicken | unpacked | Supermarket/department stores | >500g | 2015-08-24 | Shanxi | Yangquan city | Yin Ying Zhen Dong da Jie 365 Hualong Shopping Plaza supermarket | Elephant farm group in Shanxi food branch | 20150615 |
| SA039 | Raw meat | Pork | unpacked | farmer’s market/street vendors | >500g | 2015-05-11 | Hebei | Baoding City | New State Road, Zhou Jia Zhuang farmers market 7 Booth | Unknown | 20150511 |
| SA040 | Raw meat | Pork | unpacked | farmer’s market/street vendors | >500g | 2015-06-08 | Hebei | In Chengde city | He Dong Tong Xiang Jie convenience market | Unknown | 20150606 |
| SA041 | Raw meat | Duck | unpacked | Supermarket/department stores | >500g | 2015-06-08 | Hebei | In Chengde city | East Main Street hundreds of supermarkets | Unknown | 20150608 |
| SA042 | Raw meat | Duck | unpacked | farmer’s market/street vendors | >500g | 2015-06-08 | Hebei | In Chengde city | Tai Shan Lu Shi | Unknown | 20150608 |
| SA043 | Raw meat | Mutton | unpacked | farmer’s market/street vendors | >500g | 2015-08-17 | Hebei | In Chengde city | He Dong Tong Xiang Jie convenience market | Unknown | 20150817 |
| SA044 | Raw meat | Duck | unpacked | Supermarket/department stores | >500g | 2015-08-17 | Hebei | In Chengde city | Bliss Anju Street supermarket | Unknown | 20150817 |
| SA045 | Raw meat | Chicken | unpacked | Supermarket/department stores | >500g | 2015-08-17 | Hebei | In Chengde city | Wulie road, Royal Plaza B RT-Mart supermarket | Unknown | 20150817 |
| SA046 | Raw meat | Duck | unpacked | Supermarket/department stores | >500g | 2015-04-13 | Hebei | Cangzhou city | Cocoon UBS city Street Mall | Unknown | 20150422 |
| SA047 | Raw meat | Duck | unpacked | Supermarket/department stores | >500g | 2015-04-13 | Hebei | Cangzhou city | Cocoon, city streets and shopping malls | Big food group | 20150422 |
| SA048 | Raw meat | Pork | unpacked | farmer’s market/street vendors | >500g | 2015-05-11 | Hebei | Baoding City | Kou Zhou, West Zhongshan Road market meat | Unknown | 2015-5-11 |
| SA049 | Raw meat | Chicken | unpacked | farmer’s market/street vendors | >500g | 2015-04-07 | Hebei | Hengshui city | Wang Lin Heping Road Hotel | Zhou Wang Lin hotel | 20150407 |
| SA050 | Raw meat | Chicken | unpacked | farmer’s market/street vendors | >500g | 2015-04-07 | Hebei | Hengshui city | Wang Lin Heping Road Hotel | Zhou Wang Lin hotel | 20150407 |
| SA051 | Raw meat | Mutton | unpacked | farmer’s market/street vendors | >500g | 2015-04-08 | Hebei | Hengshui city | Yucai Street farmer's market fresh halal meat shop | Yucai Street, Anping County farmer's market fresh halal meat shop | 20150408 |
| SA052 | Raw meat | Duck | unpacked | farmer’s market/street vendors | >500g | 2015-07-13 | Hebei | Hengshui city | Castle roast duck restaurant | Shandong Sanhe food co | 20150705 |
| SA053 | Raw meat | Chicken | unpacked | farmer’s market/street vendors | >500g | 2015-07-13 | Hebei | Hengshui city | South of market fish shop | Unknown | 20150713 |
| SA054 | Raw meat | Chicken | unpacked | farmer’s market/street vendors | >500g | 2015-07-13 | Hebei | Hengshui city | Wang Dong South market white | Unknown | 20150713 |
| SA055 | Raw meat | Chicken | unpacked | farmer’s market/street vendors | >500g | 2015-07-13 | Hebei | Hengshui city | Industrial Street farmers market white shop | Unknown | 20150713 |
| SA056 | Raw meat | Beef | unpacked | farmer’s market/street vendors | >500g | 2015-10-13 | Hebei | Langfang city | Middle section of Heping road and sheep boss hotel | Xincheng meat co | 20151013 |
| SA057 | Raw meat | Mutton | unpacked | farmer’s market/street vendors | >500g | 2015-10-13 | Hebei | Langfang city | Chengguan middle section of Wing Cheung Street horses, beef and mutton shops | Dachang County Wei Zi Zhuang 犇犇 meat processing plant | 20151013 |
| SA058 | Raw meat | Pork | unpacked | farmer’s market/street vendors | >500g | 2015-04-28 | Hebei | In Qinhuangdao city | Bridge market 113 | Bridge market | 2015.4.28 |
| SA059 | Raw meat | Chicken | unpacked | farmer’s market/street vendors | >500g | 2015-05-05 | Hebei | Shijiazhuang | Yucai Street 21 Fino restaurant | Unknown | 2015.10.12 |
| SA060 | Raw meat | Mutton | unpacked | farmer’s market/street vendors | >500g | 2015-07-07 | Hebei | Shijiazhuang | East of the middle section of North ring road Bureau of six CIS hotel | Unknown | 2015.10.12 |
| SA061 | Raw meat | Duck | unpacked | Supermarket/department stores | >500g | 2015-07-08 | Hebei | Shijiazhuang | Xinkai road and Hall Street southbound 100 Mi Weiming supermarket | Unknown | 20150705 |
| SA062 | Raw meat | Chicken | unpacked | farmer’s market/street vendors | >500g | 2015-07-06 | Hebei | Tangshan City | Bei Lu, Tang Lin Yangyang hotel | Unknown | 2015.10.12 |
| SA063 | Raw meat | Pork | unpacked | farmer’s market/street vendors | >500g | 2015-08-10 | Hebei | Tangshan City | Moving An 恵chang Street, Tangshan City 666 Jiujiang hotels | Unknown | 2015.8.10 |
| SA064 | Raw meat | Pork | unpacked | farmer’s market/street vendors | >500g | 2015-10-12 | Hebei | Tangshan City | Trade in bei er Huan XI road, Tangshan zunhua city farmers ' market 24 | Unknown | 2015.10.12 |
| SA065 | Raw meat | Duck | unpacked | Supermarket/department stores | >500g | 2015-10-12 | Hebei | Tangshan City | Guye district, Tangshan City, linxixinlindao 59 Linxi, tengda supermarket | Unknown | 2015.10.12 |
| SA066 | Raw meat | Duck | unpacked | farmer’s market/street vendors | >500g | 2015-10-12 | Hebei | Tangshan City | Longjiang, Guye district, Tangshan City, North China the West within the small community North of linxi city | Unknown | 2015.10.12 |
| SA067 | Raw meat | Chicken | unpacked | farmer’s market/street vendors | >500g | 2015-10-12 | Hebei | Tangshan City | Guye district, Tangshan City, Beijing Road, North forest road intersection-British restaurant | Unknown | 2015.10.11 |
| SA068 | Raw meat | Duck | unpacked | farmer’s market/street vendors | >500g | 2015-10-12 | Hebei | Tangshan City | Guye district, Tangshan City, Beijing Road, North forest road intersection-British restaurant | Unknown | 2015.10.12 |
| SA069 | Raw meat | Chicken | unpacked | farmer’s market/street vendors | >500g | 2015-08-10 | Hebei | Tangshan City | Tate moved to An 恵quan Street, Tangshan City farmers ' markets grocery stores | Unknown | 2015.8.10 |
| SA070 | Raw meat | Beef | unpacked | Supermarket/department stores | >500g | 2015-10-12 | Hebei | Tangshan City | Zunhua LU LU Dong Jin Ke long supermarket co gold margin supermarket | Unknown | 2015.10.12 |
| SA071 | Raw meat | Chicken | unpacked | farmer’s market/street vendors | >500g | 2015-10-12 | Hebei | Zhangjiakou City | Wenchang road, shacheng town street market 7 Wen Hsing chicken processing shop | Unknown | 2015-10-12 |
| SA072 | Raw meat | Mutton | unpacked | farmer’s market/street vendors | >500g | 2015-07-21 | Yunnan | Chuxiong Yi Autonomous Prefecture | Gonghe town Duan Ping Chuan farmers ' markets stalls | Hui long village, Lv Hezhen shady village in Chuxiong city 4 | 2015-7-21 |
| SA073 | Raw meat | Beef | unpacked | farmer’s market/street vendors | >500g | 2015-07-22 | Yunnan | Chuxiong Yi Autonomous Prefecture | Shishan town, South Street farmer's market fresh halal beef | Plug plug the town's Bazaar, wuding County sub-Yun Yan | 2015-7-22 |
| SA074 | Raw meat | Duck | unpacked | Supermarket/department stores | >500g | 2015-04-21 | Yunnan | Dehong Dai and Jingpo autonomous prefecture | Unity Street 114 Better homes, supermarkets | Chengdu Feng Feng foodstuff co | 20150326 |
| SA075 | Raw meat | Duck | unpacked | farmer’s market/street vendors | >500g | 2015-10-12 | Yunnan | Dehong Dai and Jingpo autonomous prefecture | The first farmers ' market 40 | Unknown | 20151012 |
| SA076 | Raw meat | Beef | unpacked | farmer’s market/street vendors | >500g | 2015-04-14 | Yunnan | Dali Bai autonomous prefecture | ER he XI Lu, Xia Guan town mark for city farmers ' market 11 Booth | Ma Jianping sold | 2015.04.08 |
| SA077 | Raw meat | Pork | unpacked | farmer’s market/street vendors | >500g | 2015-04-08 | Yunnan | The diqing Tibetan Autonomous Prefecture | Second long Avenue farmers ' market 4-13 | Gu Cheng liang, Jiang Hua livestock slaughter Ltd | 2015.04.08 |
| SA078 | Raw meat | Beef | unpacked | farmer’s market/street vendors | >500g | 2015-04-08 | Yunnan | The diqing Tibetan Autonomous Prefecture | Dong Wang Lu Xiang Yang market 5 | La weisong slaughterhouse | 2015.9.9 |
| SA079 | Raw meat | Chicken | unpacked | farmer’s market/street vendors | >500g | 2015-06-23 | Yunnan | Honghe Hani and Yi Autonomous | Ling Chuan East Road, West side farmers market kansa ecological farming shop | Unknown | 2015.6.23 |
| SA080 | Raw meat | Duck | unpacked | farmer’s market/street vendors | >500g | 2015-06-23 | Yunnan | Honghe Hani and Yi Autonomous | Chuan Zi Ling Chuan East Road, West side farmers ' markets chicken shop | Unknown | 2015.6.23 |
| SA081 | Raw meat | Chicken | unpacked | Supermarket/department stores | >500g | 2015-06-23 | Yunnan | Honghe Hani and Yi Autonomous | Tian Ma, supermarkets | Unknown | 2015.8.25 |
| SA082 | Raw meat | Chicken | unpacked | farmer’s market/street vendors | >500g | 2015-08-25 | Yunnan | Honghe Hani and Yi Autonomous | Jin hua Lu XING long farmers market 29-30 XING long live chicken retail store | Unknown | 2015.6.11 |
| SA083 | Raw meat | Mutton | unpacked | farmer’s market/street vendors | >500g | 2015-06-11 | Yunnan | Honghe Hani and Yi Autonomous | Day Street farmer's market in Kowloon boss Lee booth | Unknown | 2015-4-27 |
| SA084 | Raw meat | Chicken | unpacked | farmer’s market/street vendors | >500g | 2015-04-27 | Yunnan | Kunming city | Kunyang town, harmony road Kunyang South Gate market 1 , kill a chicken shop | Unknown | 2015-7-13 |
| SA085 | Raw meat | Chicken | unpacked | farmer’s market/street vendors | >500g | 2015-07-13 | Yunnan | Kunming city | Ma Nan lu, 212 SsangYong, markets of live poultry trading areas 2 Booth (global license) | Unknown | 2015-7-15 |
| SA086 | Raw meat | Chicken | unpacked | farmer’s market/street vendors | >500g | 2015-07-15 | Yunnan | Kunming city | Yu Lu 10 Rendezhen bei Ying WPI farmers market meat trading area 20 stalls (global license) | Unknown | 2015.10.26 |
| SA087 | Raw meat | Pork | unpacked | farmer’s market/street vendors | >500g | 2015-03-31 | Yunnan | Lincang city | Mengmeng bei Lu, farmer's market fresh meat 2 Booth | Shuangjiang County, Yunnan province, Meng-e Li Zong, mengmengzhen farms | 2015.3.30 |
| SA088 | Raw meat | Beef | unpacked | farmer’s market/street vendors | >500g | 2015-03-30 | Yunnan | Lincang city | Renmin Road pedestrian street 254 Beef restaurant | Composite, lincang city, Yunnan province, village and old family cattle farm | 20150720 |
| SA089 | Raw meat | Chicken | unpacked | farmer’s market/street vendors | >500g | 2015-06-05 | Yunnan | Lijiang city | Dayan offices long Zhong Yi farmers market live poultry, water district | Unknown | 20150803 |
| SA090 | Raw meat | Chicken | unpacked | farmer’s market/street vendors | >500g | 2015-08-03 | Yunnan | Lijiang city | Xiang he Chu Nan lu JI Nong Mao Shi Chang C-001 Yong ping live poultry sales points | Unknown | 20150803 |
| SA091 | Raw meat | Chicken | unpacked | farmer’s market/street vendors | >500g | 2015-08-03 | Yunnan | Lijiang city | Dayan Office longest waterway and upright farmer's market fresh 60 Booth | Unknown | 20150609 |
| SA092 | Raw meat | Mutton | unpacked | farmer’s market/street vendors | >500g | 2015-06-09 | Yunnan | Lijiang city | Dayan offices long waterway loyalty and farmers ' markets for beef and mutton 376 | Unknown | 20150707 |
| SA093 | Raw meat | Mutton | unpacked | farmer’s market/street vendors | >500g | 2015-07-07 | Yunnan | Lijiang city | Dayan offices long waterway loyalty and farmers ' markets for beef and mutton 374 | Gucheng district, Lijiang city, Jin Shan Xiang Dawa goat farming co | 20151013 |
| SA094 | Raw meat | Beef | unpacked | farmer’s market/street vendors | >500g | 2015-10-13 | Yunnan | Lijiang city | Xian Office to Fu Hui Lu Fuhui farmers market meat stalls 001 | Unknown | 2015-7-13 |
| SA095 | Raw meat | Chicken | unpacked | farmer’s market/street vendors | >500g | 2015-06-08 | Yunnan | Pu-Erh tea | Ding Lu, Gao Jia Zhai district farmer's market fresh meat class 24 Sun Qiongzhen, booth | Unknown | 20150608 |
| SA096 | Raw meat | Chicken | unpacked | farmer’s market/street vendors | >500g | 2015-06-08 | Yunnan | Pu-Erh tea | Border town road 10 Number 51 farmers market E7-2 Li Chengji booth | Jia Liyou farmers | 20150608 |
| SA097 | Raw meat | Chicken | unpacked | Supermarket/department stores | >500g | 2015-07-28 | Yunnan | Pu-Erh tea | Zhenxing road, Daxing discount supermarkets | Liu Wencai farmers | 20151012 |
| SA098 | Raw meat | Duck | unpacked | farmer’s market/street vendors | >500g | 2015-10-12 | Yunnan | Pu-Erh tea | Border town road 10 Number 51 farmers market E5-5 Qi Jiahai booth | Simao district in Simao town bridge crane nest farm | 20150720 |
| SA099 | Raw meat | Chicken | unpacked | farmer’s market/street vendors | >500g | 2015-06-30 | Yunnan | Kunming city | News community news road 7-98 Pure chicken shop in Wenshan | Unknown | 20150630 |
| SA100 | Raw meat | Chicken | unpacked | Supermarket/department stores | >500g | 2015-06-30 | Yunnan | Kunming city | Rokko Community farmers ' markets, Guandu district 130 Old chicken franchise store of Sichuan | Unknown | 20150915 |
| SA101 | Raw meat | Duck | unpacked | farmer’s market/street vendors | >500g | 2015-09-15 | Yunnan | Kunming city | Dong Huaxin farmer's market reputation of pure chicken shop | Unknown | 20150915 |
| SA102 | Raw meat | Chicken | unpacked | farmer’s market/street vendors | >500g | 2015-10-27 | Yunnan | Kunming city | News road community seal of new farmers ' markets in the news 7-13 | Unknown | 20151027 |
| SA103 | Raw meat | Chicken | unpacked | farmer’s market/street vendors | >500g | 2015-10-27 | Yunnan | Kunming city | News road community seal of new farmers ' markets in the news 7-26 Sea chicken chicken shop | Unknown | 20151027 |
| SA104 | Raw meat | Duck | unpacked | farmer’s market/street vendors | >500g | 2015-10-27 | Yunnan | Kunming city | Dong SI Jie Fang Xin Cun Xi SI Xiang 40 Authentic Renaissance farmers ' markets, wuding, Wenshan, zhaotong Zhuang chicken restaurant | Unknown | 20151027 |
| SA105 | Raw meat | Chicken | unpacked | Supermarket/department stores | >500g | 2015-10-27 | Yunnan | Kunming city | NaN Ping Street 88 Century Plaza, 2-3 Floor Carrefour | Unknown | 20151027 |
| SA106 | Raw meat | Chicken | unpacked | Supermarket/department stores | >500g | 2015-10-27 | Yunnan | Kunming city | NaN Ping Street 88 Century Plaza, 2-3 Floor Carrefour | Unknown | 20151027 |
| SA107 | Raw meat | Mutton | unpacked | farmer’s market/street vendors | >500g | 2015-06-20 | Yunnan | In Yuxi city | Longquan Street temporary stalls Fergus farmers ' markets Zhufengxian stalls | Yimen Pu bei Xiang culture Zhufengxian | 2015/07/06 |
| SA108 | Raw meat | Mutton | unpacked | farmer’s market/street vendors | >500g | 2015-10-27 | Liaoning | Huludao city | Yangjiazhangzi Mao Qitun Street, development zone the farmers ' market 6 Li Chunkai booth | Yangjiazhangzi Mao Qitun Street, economic development zone Li Chunkai family slaughter | 2015-10-27 |
| SA109 | Raw meat | Chicken | unpacked | Supermarket/department stores | >500g | 2015-06-15 | Liaoning | In Shenyang city | Nanjing South Street 201 RT-Mart supermarket, a long white shop | Agriculture and animal husbandry (ling) limited | 20151027 |
| SA110 | Raw meat | Beef | unpacked | farmer’s market/street vendors | >500g | 2015-10-29 | Liaoning | Huludao city | XING Gong West Street Department store line 100 M grocery wholesale market 3 Li Xin, booth | Huludao lianshan district grocery wholesale market 3 Li Xin, booth family slaughter | 2015-10-29 |
| SA111 | Raw meat | Chicken | unpacked | farmer’s market/street vendors | >500g | 2015-04-13 | Liaoning | Huludao city | Yu Huang, Jin Hu road Mall farmers ' market 647 Beautiful white chicken stalls | Agriculture and animal husbandry ( Yingkou ) Co branch of panjin | 2015-4-12 |
| SA112 | Raw meat | Beef | unpacked | farmer’s market/street vendors | >500g | 2015-10-12 | Liaoning | Yingkou city | Huaihe Lu Haitian beef 1 | Unknown | 2015-4-12 |
| SA113 | Raw meat | Pork | unpacked | farmer’s market/street vendors | >500g | 2015-05-12 | Liaoning | In Shenyang city | Fengyang road, 181 One carat market 1460 Stand | Unknown | 15-5-12 |
| SA114 | Raw meat | Duck | unpacked | farmer’s market/street vendors | >500g | 2015-07-22 | Liaoning | Huludao city | South huangxing road 32 Sea Palace catering services limited liability company, headquarters of the Jade Palace Mall sea seafood dumplings | Beijing ruichang farms | 2015-06-28 |
| SA115 | Raw meat | Chicken | unpacked | farmer’s market/street vendors | >500g | 2015-05-26 | Liaoning | Liaoyang city | Shoushanzhen people's shopping street farmers ' market | Unknown | 20150526 |
| SA116 | Raw meat | Pork | unpacked | Supermarket/department stores | >500g | 2015-10-13 | Liaoning | Liaoyang city | New Street 105 RT-Mart supermarket in Liaoyang store | Liaoyang RT commercial limited company | 20151013 |
| SA117 | Raw meat | Duck | unpacked | Supermarket/department stores | >500g | 2015-07-06 | Liaoning | Jinzhou City | Three Central Avenue 17 Hualian supermarket | Unknown | 20151013 |
| SA118 | Raw meat | Duck | unpacked | Supermarket/department stores | >500g | 2015-07-21 | Liaoning | Huludao city | South side of Chaoyang road, three high schools (turntable North) jianchang Xinglong store shopping centre limited supermarket meat counter | Unknown dealer: Wei-Ying, suizhong trading company | 2015-07-21 |
| SA119 | Raw meat | Chicken | unpacked | Supermarket/department stores | >500g | 2015-05-28 | Liaoning | Liaoyang city | New Street 105 RT-Mart supermarket in Liaoyang store | Liaoyang RT commercial limited company | 20150528 |
| SA120 | Raw meat | Chicken | unpacked | Supermarket/department stores | >500g | 2015-05-28 | Liaoning | Liaoyang city | New Street 105 RT-Mart supermarket in Liaoyang store | Liaoyang RT commercial limited company | 20150528 |
| SA121 | Raw meat | Duck | unpacked | farmer’s market/street vendors | >500g | 2015-11-16 | Liaoning | Dalian City | Changchun Road West 79 Trade building, A007 Booth | Unknown | 20151116 |
| SA122 | Raw meat | Duck | unpacked | farmer’s market/street vendors | >500g | 2015-10-29 | Liaoning | Huludao city | XING Gong West Street Department store line 100 M grocery wholesale market 101 Yandezhen booth | Linqu hengxin foods limited | 2015-10-19 |
| SA123 | Raw meat | Beef | unpacked | farmer’s market/street vendors | >500g | 2015-07-13 | Liaoning | In Dandong city | East Pearl Street road 34 Chime-long farmers market 141 Stand | Unknown | 2015-10-19 |
| SA124 | Raw meat | Pork | unpacked | farmer’s market/street vendors | >500g | 2015-07-22 | Liaoning | Huludao city | South huangxing road 32 Sea Palace catering services limited liability company, headquarters of the Jade Palace Mall sea seafood dumplings | Jinzhou source food Ltd | 2015-07-22 |
| SA125 | Raw meat | Chicken | unpacked | farmer’s market/street vendors | >500g | 2015-07-21 | Liaoning | Yingkou city | Army road state capital market chicken 3 | Unknown | 2015-07-22 |
| SA126 | Raw meat | Pork | unpacked | farmer’s market/street vendors | >500g | 2015-05-26 | Liaoning | Liaoyang city | Shoushanzhen people's shopping street farmers ' market | Unknown | 20150526 |
| SA127 | Raw meat | Chicken | unpacked | farmer’s market/street vendors | >500g | 2015-05-11 | Liaoning | In Shenyang city | Fa Ku Zhen Xiao Dong Jie long Lu 1 Products wholesale market, Wang Xin chicken stalls | Unknown | 20150526 |
| SA128 | Raw meat | Beef | unpacked | farmer’s market/street vendors | >500g | 2015-05-18 | Liaoning | Anshan city | Light Street West city pie shop | Unknown | 20150526 |
| SA129 | Raw meat | Chicken | unpacked | farmer’s market/street vendors | >500g | 2015-05-18 | Liaoning | Dalian City | Tianhe Road 139 Bai-Shun, fresh market | Tianhe Road, ganjingzi district 139 Bai-Shun, pyramids of fresh market meat stalls | 20150518 |
| SA130 | Raw meat | Beef | unpacked | Supermarket/department stores | >500g | 2015-08-03 | Liaoning | Dalian City | East Road 18 Beijing Hualian | Unknown | 2015-08-03 |
| SA131 | Raw meat | Mutton | unpacked | Supermarket/department stores | >500g | 2015-07-09 | Liaoning | Huludao city | Long middle section of road in nanpiao, street, Kowloon City supermarket meat counter | Nanpiao district streets after Huang Jia village, Huludao city, Temple slaughterhouse | 2015-07-09 |
| SA132 | Raw meat | Chicken | unpacked | Supermarket/department stores | >500g | 2015-11-02 | Liaoning | Liaoyang city | Wing Wah Street 9 Tesco, the supermarket | Liao Tesco splendor of shops | 20151101 |
| SA133 | Raw meat | Pork | unpacked | farmer’s market/street vendors | >500g | 2015-10-27 | Liaoning | Huludao city | Yangjiazhangzi yangjiazhangzi Street, economic development zone Bureau ex-Community market pork 1 Gao Honggang booth | Yangjiazhangzi, Huludao economic development zone high monks ditch slaughter | 2015-10-27 |
| SA134 | Raw meat | Mutton | unpacked | Supermarket/department stores | >500g | 2015-10-12 | Liaoning | Yingkou city | Kunlun Street junction with road commercial building for beef and mutton | Unknown | 20150824 |
| SA135 | Raw meat | Chicken | unpacked | farmer’s market/street vendors | >500g | 2015-05-18 | Liaoning | Anshan city | Prosperity Nan Jie Dong Tai an food Hall in broilers 07 | Unknown | 20150824 |
| SA136 | Raw meat | Chicken | unpacked | Supermarket/department stores | >500g | 2015-06-15 | Inner Mongoria | Hohhot | Carrefour names, South central square branch | Shandong Yucheng Maria's Foods Ltd | 20150824 |
| SA137 | Raw meat | Mutton | unpacked | Supermarket/department stores | >500g | 2015-04-13 | Inner Mongoria | Baotou city | Forest Avenue 6 Beijing Hualian supermarket, Wenhua Rd | Inner Mongolia Yi de halal meat industry food limited company | 20150413 |
| SA138 | Raw meat | Beef | unpacked | Supermarket/department stores | >500g | 2015-04-13 | Inner Mongoria | Baotou city | Forest Avenue 6 Beijing Hualian supermarket, Wenhua Rd | IFON beef | 20150413 |
| SA139 | Raw meat | Chicken | unpacked | Supermarket/department stores | >500g | 2015-07-06 | Inner Mongoria | Baotou city | Tai Nan Street 8 Insein, supermarket | Rhonda and six farming co | 20150411 |
| SA140 | Raw meat | Chicken | unpacked | Supermarket/department stores | >500g | 2015-07-06 | Inner Mongoria | Baotou city | Forest Avenue 6 Beijing Hualian supermarket, Wenhua Rd | Three thaw foods limited | 20150605 |
| SA141 | Raw meat | Chicken | unpacked | Supermarket/department stores | >500g | 2015-07-06 | Inner Mongoria | Baotou city | Forest Avenue 6 Beijing Hualian supermarket, Wenhua Rd | M eco-food science and technology company limited | 20150326 |
| SA142 | Raw meat | Pork | unpacked | Supermarket/department stores | >500g | 2015-08-24 | Inner Mongoria | Baotou city | Bayantala Street 48 Victor's supermarket East store | Orchard poultry slaughterhouses | 20150824 |
| SA143 | Raw meat | Pork | unpacked | Supermarket/department stores | >500g | 2015-08-24 | Inner Mongoria | Baotou city | Bayantala Street 48 Victor's supermarket East store | Orchard poultry slaughterhouses | 20150824 |
| SA144 | Raw meat | Pork | unpacked | Supermarket/department stores | >500g | 2015-08-24 | Inner Mongoria | Baotou city | Bayantala Street 48 Victor's supermarket East store | Orchard poultry slaughterhouses | 20150824 |
| SA145 | Raw meat | Chicken | unpacked | Supermarket/department stores | >500g | 2015-09-07 | Inner Mongoria | Baotou city | Lark Street 1 Munda, the supermarket | Inner Mongolia Kangxin foods limited | 20150505 |
| SA146 | Raw meat | Pork | unpacked | Supermarket/department stores | >500g | 2015-09-07 | Inner Mongoria | Baotou city | Allah Tower Avenue 50 Insein, supermarket | Unknown | 20150907 |
| SA147 | Raw meat | Chicken | unpacked | farmer’s market/street vendors | >500g | 2015-10-26 | Inner Mongoria | Baotou city | Fu Qiang Lu 42 Its prosperous market | Swan foods limited | 20151026 |
| SA148 | Raw meat | Chicken | unpacked | Supermarket/department stores | >500g | 2015-10-26 | Inner Mongoria | Baotou city | Culture road 85 Hundreds of supermarkets | Hohhot, fine foods, Ltd | 20151026 |
| SA149 | Raw meat | Chicken | unpacked | Supermarket/department stores | >500g | 2015-07-21 | Inner Mongoria | ALXA League | Tiansheng Bayanhaote Town South Street supermarket | Unknown | 20150620 |
| SA150 | Raw meat | Chicken | unpacked | farmer’s market/street vendors | >500g | 2015-05-18 | Inner Mongoria | Wuhai city | Yellow River Yellow River Road market Chicken fish seafood does a small wholesale | Unknown | 20150907 |
| SA151 | Raw meat | Beef | unpacked | Supermarket/department stores | >500g | 2015-06-09 | Inner Mongoria | Wuhai city | Joy city qianlishan Street first floor Xinhua department store supermarket | Unknown | 20150907 |
| SA152 | Raw meat | Beef | unpacked | Supermarket/department stores | >500g | 2015-06-09 | Inner Mongoria | Wuhai city | Joy city qianlishan Street first floor Xinhua department store supermarket | Unknown | 20150907 |
| SA153 | Raw meat | Chicken | unpacked | farmer’s market/street vendors | >500g | 2015-04-13 | Inner Mongoria | Ordos city | Aolezhaoqi Tao Nan Lu Wei Honge Tauren market meat sales | Etkqq Wei Honge meat sales | 20150413 |
| SA154 | Raw meat | Duck | unpacked | farmer’s market/street vendors | >500g | 2015-04-13 | Inner Mongoria | Ordos city | Aolezhaoqi Tao Nan lu Gao Shuping Tauren market meat sales | No | 20150413 |
| SA155 | Raw meat | Chicken | unpacked | farmer’s market/street vendors | >500g | 2015-07-20 | Inner Mongoria | Wulanchabu city | Road for the benefit of workers and peasants Park farmers ' market ingredients | Dalian zhongxin foods limited | 20150907 |
| SA156 | Raw meat | Chicken | unpacked | Supermarket/department stores | >500g | 2015-04-13 | Inner Mongoria | Xilin GOL League | Xilin Avenue middle section of China resources Vanguard supermarket | Grassland xingfa foods limited | 2014-05-10 |
| SA157 | Raw meat | Chicken | unpacked | Supermarket/department stores | >500g | 2015-04-13 | Inner Mongoria | Xilin GOL League | Xilin Avenue middle part of Victor's supermarket | Dalian shengda foods limited | 2015-01-20 |
| SA158 | Raw meat | Mutton | unpacked | farmer’s market/street vendors | >500g | 2015-09-14 | Inner Mongoria | Xilin GOL League | Direct sale Office in Tuan Jie Jie Yi Xing red meat the meat industry | Unknown | 2015-09-10 |
| SA159 | Raw meat | Mutton | unpacked | Supermarket/department stores | >500g | 2015-09-14 | Inner Mongoria | Xilin GOL League | Tao Lin Road pony grocery supermarket | Unknown | 2015-09-05 |
| SA160 | Raw meat | Chicken | unpacked | farmer’s market/street vendors | >500g | 2015-09-14 | Inner Mongoria | Xilin GOL League | Unity Street, Sau-Ling smoked chicken, smoked rabbit shop | Unknown | 2015-08-11 |
| SA161 | Raw meat | Mutton | unpacked | farmer’s market/street vendors | >500g | 2015-10-13 | Inner Mongoria | Xilin GOL League | Market Street Bridge before the butcher's | Ominous | 2015-10-13 |
| SA162 | Raw meat | Pork | unpacked | farmer’s market/street vendors | >500g | 2015-08-25 | Inner Mongoria | In chifeng city | Da ban Zhen slab Jie Zhao Sanjie large integrated market of fresh pork shop | Unknown | 2015-08-11 |
| SA163 | Raw meat | Beef | unpacked | Supermarket/department stores | >500g | 2015-08-25 | Inner Mongoria | In chifeng city | Sylla Street love Wanda Plaza supermarket | Unknown | 2015-08-11 |
| SA164 | Raw meat | Pork | unpacked | farmer’s market/street vendors | >500g | 2015-10-15 | Inner Mongoria | In chifeng city | Xin Hui Lu, Xin Hui Zhen Xin Hui Fu source meat outlet on the second floor of the market | Fu source meat processing co | 20151015 |
| SA165 | Raw meat | Mutton | unpacked | farmer’s market/street vendors | >500g | 2015-10-15 | Inner Mongoria | In chifeng city | New city Tianyi Lu Ying bin market for halal meat | Unknown | 20151015 |
| SA166 | Raw meat | Chicken | unpacked | Supermarket/department stores | >500g | 2015-10-28 | Inner Mongoria | In chifeng city | Lin XI Zhen linxi Avenue Futura to the supermarket | Shuofeng animal, chifeng city, Inner Mongolia foods limited | 20150703 |
| SA167 | Raw meat | Chicken | unpacked | farmer’s market/street vendors | >500g | 2015-10-13 | Inner Mongoria | , Hinggan League, | Lin Street night market 18 | Unknown | 2015-08-11 |
| SA168 | Raw meat | Pork | unpacked | farmer’s market/street vendors | >500g | 2015-09-14 | Shaanxi | In Ankang city | Jingning Road market 8 Meat | Unknown | 20150914 |
| SA169 | Raw meat | Pork | unpacked | farmer’s market/street vendors | >500g | 2015-09-14 | Shaanxi | In Ankang city | Jingning Road market 14 Meat | Unknown | 20150914 |
| SA170 | Raw meat | Pork | unpacked | farmer’s market/street vendors | >500g | 2015-09-14 | Shaanxi | In Ankang city | Jingning Road market 18 Meat | Unknown | 20150914 |
| SA171 | Raw meat | Pork | unpacked | farmer’s market/street vendors | >500g | 2015-09-14 | Shaanxi | In Ankang city | South Gate market, nanhuan shuanghui chilled meat home store | Unknown | 20150914 |
| SA172 | Raw meat | Mutton | unpacked | farmer’s market/street vendors | >500g | 2015-10-12 | Shaanxi | In Ankang city | Wu Feng road farmers ' market | Unknown | 20151011 |
| SA173 | Raw meat | Chicken | unpacked | farmer’s market/street vendors | >500g | 2015-10-12 | Shaanxi | In Ankang city | Wufeng road farmers ' market of domestic chicken shop | Unknown | 20151012 |
| SA174 | Raw meat | Duck | unpacked | Supermarket/department stores | >500g | 2015-10-12 | Shaanxi | In Ankang city | Run in Xin Zheng Jie billion supermarket | Unknown | 20150914 |
| SA175 | Raw meat | Chicken | unpacked | farmer’s market/street vendors | >500g | 2015-10-19 | Shaanxi | In Ankang city | Jingning Lu Zhou Qiaoxian chicken wholesale and retail stores | Our shop | 20151019 |
| SA176 | Raw meat | Chicken | unpacked | farmer’s market/street vendors | >500g | 2015-10-19 | Shaanxi | In Ankang city | Jinzhou hospital Lu XING an farmers ' markets back door | No | 20150914 |
| SA177 | Raw meat | Duck | unpacked | Supermarket/department stores | >500g | 2015-10-19 | Shaanxi | In Ankang city | Bashan East Road, Minsheng Jiale health shopping mall | Food and Echeng six limited | 20150918 |
| SA178 | Raw meat | Chicken | unpacked | farmer’s market/street vendors | >500g | 2015-11-02 | Shaanxi | In Ankang city | Jingning road farmers ' market before reaching the chicken stall | This booth | 20151102 |
| SA179 | Raw meat | Duck | unpacked | Supermarket/department stores | >500g | 2015-11-09 | Shaanxi | In Ankang city | Fang Lu, China resources Vanguard supermarket | Shandong Taian Jinquan agricultural company | 20150519 |
| SA180 | Raw meat | Duck | unpacked | Supermarket/department stores | >500g | 2015-11-09 | Shaanxi | In Ankang city | Fang Lu, China resources Vanguard supermarket | Unknown | 20150519 |
| SA181 | Raw meat | Pork | unpacked | farmer’s market/street vendors | >500g | 2015-04-14 | Shaanxi | Baoji city | People's street market 68 | Unknown | 20150414 |
| SA182 | Raw meat | Beef | unpacked | farmer’s market/street vendors | >500g | 2015-04-14 | Shaanxi | Baoji city | People's street market 54 | Unknown | 20150414 |
| SA183 | Raw meat | Pork | unpacked | farmer’s market/street vendors | >500g | 2015-05-19 | Shaanxi | Baoji city | Southern red meat in the market of agricultural products wholesale | Unknown | 20150519 |
| SA184 | Raw meat | Mutton | unpacked | farmer’s market/street vendors | >500g | 2015-05-19 | Shaanxi | Baoji city | Southern agricultural market halal meat shop | Unknown | 20150519 |
| SA185 | Raw meat | Chicken | unpacked | farmer’s market/street vendors | >500g | 2015-05-19 | Shaanxi | Baoji city | Nancheng Samsung seafood seasoning cooked meat wholesalers in the market of agricultural products | Jilin light farm products company | 20150419 |
| SA186 | Raw meat | Pork | unpacked | farmer’s market/street vendors | >500g | 2015-06-23 | Shaanxi | Baoji city | Shuangshipu farmers market | Unknown | 20150623 |
| SA187 | Raw meat | Pork | unpacked | farmer’s market/street vendors | >500g | 2015-06-23 | Shaanxi | Baoji city | Shuangshipu farmers market | Unknown | 20150623 |
| SA188 | Raw meat | Chicken | unpacked | farmer’s market/street vendors | >500g | 2015-06-23 | Shaanxi | Baoji city | Shuangshipu farmers market Feng Fei chicken | Unknown | 20151027 |
| SA189 | Raw meat | Duck | unpacked | farmer’s market/street vendors | >500g | 2015-06-23 | Shaanxi | Baoji city | Shuangshipu farmers market Feng Fei chicken | Unknown | 2015.5.18 |
| SA190 | Raw meat | Pork | unpacked | Supermarket/department stores | >500g | 2015-07-21 | Shaanxi | Baoji city | Home mega Fengxiang donghu | Unknown | 2015.5.18 |
| SA191 | Raw meat | Mutton | unpacked | farmer’s market/street vendors | >500g | 2015-09-15 | Shaanxi | Baoji city | Unknown | Qianyang County market halal meat shop | 20150915 |
| SA192 | Raw meat | Mutton | unpacked | farmer’s market/street vendors | >500g | 2015-10-27 | Shaanxi | Baoji city | People's street market | Halal market first | 20151027 |
| SA193 | Raw meat | Pork | unpacked | farmer’s market/street vendors | >500g | 2015-10-27 | Shaanxi | Baoji city | People's street market | Unknown | 20151027 |
| SA194 | Raw meat | Chicken | unpacked | farmer’s market/street vendors | >500g | 2015-11-03 | Shaanxi | Baoji city | Two lane road vegetable wholesale market | Unknown | 20151103 |
| SA195 | Raw meat | Pork | unpacked | farmer’s market/street vendors | >500g | 2015-04-13 | Shaanxi | Hanzhong city | Zhou Jia town, Nanhu road farmers ' market | Santan | 2015.4.13 |
| SA196 | Raw meat | Mutton | unpacked | farmer’s market/street vendors | >500g | 2015-05-18 | Shaanxi | Hanzhong city | West Street cross halal meat market | Santan | 2015.5.18 |
| SA197 | Raw meat | Pork | unpacked | farmer’s market/street vendors | >500g | 2015-05-18 | Shaanxi | Hanzhong city | Wenchang road farmers ' market | Santan | 2015.5.18 |
| SA198 | Raw meat | Pork | unpacked | farmer’s market/street vendors | >500g | 2015-06-15 | Shaanxi | Hanzhong city | Farmers market, market road Santan | Santan | 2015.6.15 |
| SA199 | Raw meat | Pork | unpacked | farmer’s market/street vendors | >500g | 2015-06-15 | Shaanxi | Hanzhong city | Farmers market, market road Santan | Santan | 2015.6.15 |
| SA200 | Raw meat | Pork | unpacked | farmer’s market/street vendors | >500g | 2015-06-15 | Shaanxi | Hanzhong city | Farmers market, market road Santan | Santan | 2015.6.15 |
| SA201 | Raw meat | Pork | unpacked | farmer’s market/street vendors | >500g | 2015-08-24 | Shaanxi | Hanzhong city | Snow King Xiang Yu store | In-store produced | 2015.8.24 |
| SA202 | Raw meat | Pork | unpacked | farmer’s market/street vendors | >500g | 2015-08-24 | Shaanxi | Hanzhong city | West city farmers ' market after Santan | Santan | 2015.8.24 |
| SA203 | Raw meat | Pork | unpacked | farmer’s market/street vendors | >500g | 2015-08-24 | Shaanxi | Hanzhong city | Crown King Xiang Yu Guo Jie Lou outlet store | In-store produced | 2015.8.24 |
| SA204 | Raw meat | Duck | unpacked | farmer’s market/street vendors | >500g | 2015-09-15 | Shaanxi | Hanzhong city | King Xiang Yu Xia Yuan and shops | In-store produced | 2015.8.24 |
| SA205 | Raw meat | Duck | unpacked | farmer’s market/street vendors | >500g | 2015-09-15 | Shaanxi | Hanzhong city | Zhang Zhou Jia town, integrated market and shops | In-store produced | 2015.8.24 |
| SA206 | Raw meat | Chicken | unpacked | farmer’s market/street vendors | >500g | 2015-09-15 | Shaanxi | Hanzhong city | Zhou Jia Li DAO ping Zhen integrated market and shops | In-store produced | 2015.06.29 |
| SA207 | Raw meat | Chicken | unpacked | farmer’s market/street vendors | >500g | 2015-09-15 | Shaanxi | Hanzhong city | Zhang Zhou Jia town, integrated market and shops | In-store produced | 2015.06.29 |
| SA208 | Raw meat | Pork | unpacked | Supermarket/department stores | >500g | 2015-10-12 | Shaanxi | Hanzhong city | West Loop huasheng supermarket | Supermarket produce | 2015.10.12 |
| SA209 | Raw meat | Pork | unpacked | farmer’s market/street vendors | >500g | 2015-10-12 | Shaanxi | Hanzhong city | West city farmers ' market after nameless Santan | Santan | 2015.10.12 |
| SA210 | Raw meat | Pork | unpacked | Supermarket/department stores | >500g | 2015-10-12 | Shaanxi | Hanzhong city | Tian Han road, Minsheng Jiale supermarket | Supermarket produce | 2015.10.12 |
| SA211 | Raw meat | Pork | unpacked | farmer’s market/street vendors | >500g | 2015-05-20 | Shaanxi | In shangluo city | Solidarity road farmers ' market stalls | Unknown | 2015.05.20 |
| SA212 | Raw meat | Beef | unpacked | farmer’s market/street vendors | >500g | 2015-05-20 | Shaanxi | In shangluo city | Solidarity road farmers ' market stalls | Unknown | 2015.05.20 |
| SA213 | Raw meat | Pork | unpacked | farmer’s market/street vendors | >500g | 2015-05-20 | Shaanxi | In shangluo city | Solidarity road farmers market-River to remember the butcher's | Unknown | 2015.05.20 |
| SA214 | Raw meat | Mutton | unpacked | farmer’s market/street vendors | >500g | 2015-05-20 | Shaanxi | In shangluo city | Solidarity road farmers ' market stalls | Unknown | 2015.05.20 |
| SA215 | Raw meat | Mutton | unpacked | farmer’s market/street vendors | >500g | 2015-05-20 | Shaanxi | In shangluo city | Solidarity road farmers ' market stalls | Unknown | 2015.05.20 |
| SA216 | Raw meat | Beef | unpacked | farmer’s market/street vendors | >500g | 2015-05-20 | Shaanxi | In shangluo city | Solidarity road farmers ' market stalls | Unknown | 2015.05.20 |
| SA217 | Raw meat | Chicken | unpacked | farmer’s market/street vendors | >500g | 2015-09-06 | Shaanxi | In shangluo city | United farmers market chicken shop | Unknown | 2015.09.06 |
| SA218 | Raw meat | Chicken | unpacked | farmer’s market/street vendors | >500g | 2015-09-06 | Shaanxi | In shangluo city | United farmers market chicken shop | Unknown | 2015.09.06 |
| SA219 | Raw meat | Duck | unpacked | farmer’s market/street vendors | >500g | 2015-09-06 | Shaanxi | In shangluo city | United farmers market chicken shop | Unknown | 2015.09.06 |
| SA220 | Raw meat | Chicken | unpacked | farmer’s market/street vendors | >500g | 2015-06-29 | Shaanxi | XI ' an city | Chicken segment of xixiang street market shop | Production | 2015.06.29 |
| SA221 | Raw meat | Duck | unpacked | farmer’s market/street vendors | >500g | 2015-06-29 | Shaanxi | XI ' an city | Chicken segment of xixiang street market shop | Unknown | 2015.06.29 |
| SA222 | Raw meat | Chicken | unpacked | Supermarket/department stores | >500g | 2015-06-29 | Shaanxi | XI ' an city | Autumn forest company Li Jia village shop | Unknown | 2015.06.29 |
| SA223 | Raw meat | Chicken | unpacked | Supermarket/department stores | >500g | 2015-06-29 | Shaanxi | XI ' an city | Autumn forest company Li Jia village shop | Unknown | 2015.06.29 |
| SA224 | Raw meat | Chicken | unpacked | farmer’s market/street vendors | >500g | 2015-06-29 | Shaanxi | XI ' an city | Founding of comprehensive market fresh chicken | Unknown | 2015.06.29 |
| SA225 | Raw meat | Duck | unpacked | farmer’s market/street vendors | >500g | 2015-06-29 | Shaanxi | XI ' an city | Founding of comprehensive market fresh chicken | Unknown | 2015.06.29 |
| SA226 | Raw meat | Chicken | unpacked | farmer’s market/street vendors | >500g | 2015-06-29 | Shaanxi | XI ' an city | Founding of comprehensive market June frozen shop | Unknown | 2015.06.29 |
| SA227 | Raw meat | Duck | unpacked | farmer’s market/street vendors | >500g | 2015-10-27 | Shaanxi | XI ' an city | Horse mausoleum nameless shop | Unknown | 2015.8.10 |
| SA228 | Raw meat | Chicken | unpacked | farmer’s market/street vendors | >500g | 2015-10-27 | Shaanxi | XI ' an city | Jianguomen market nameless shop | Unknown | 2015.8.10 |
| SA229 | Raw meat | Chicken | unpacked | farmer’s market/street vendors | >500g | 2015-10-27 | Shaanxi | XI ' an city | Jianguomen market fresh chicken | Unknown | 2015.8.10 |
| SA230 | Raw meat | Chicken | unpacked | Supermarket/department stores | >500g | 2015-10-27 | Shaanxi | XI ' an city | Wal-Mart Li Jia Cun, Wanda | Unknown | 2015.8.10 |
| SA231 | Raw meat | Chicken | unpacked | farmer’s market/street vendors | >500g | 2015-10-27 | Shaanxi | XI ' an city | XI Jie Shi pengxiang aquatic shop | Unknown | 2015.8.10 |
| SA232 | Raw meat | Chicken | unpacked | Supermarket/department stores | >500g | 2015-10-27 | Shaanxi | XI ' an city | CRV Yanta Northern road | Unknown | 2015.8.10 |
| SA233 | Raw meat | Chicken | unpacked | Supermarket/department stores | >500g | 2015-10-27 | Shaanxi | XI ' an city | CRV Yanta Northern road | Shanxi as agriculture and animal husbandry science and technology limited company | 2015.8.10 |
| SA234 | Raw meat | Chicken | unpacked | Supermarket/department stores | >500g | 2015-10-27 | Shaanxi | XI ' an city | Autumn forest company Li Jia village shop | Shanxi Dan Feng Hua Mao livestock | 2015.8.10 |
| SA235 | Raw meat | Beef | unpacked | farmer’s market/street vendors | >500g | 2015-06-29 | Shaanxi | Tongchuan city | Red Bridge market halal beef in stone shop | Production | 2015.6.29 |
| SA236 | Raw meat | Mutton | unpacked | farmer’s market/street vendors | >500g | 2015-06-29 | Shaanxi | Tongchuan city | Red Bridge market halal beef in stone shop | Production | 2015.6.29 |
| SA237 | Raw meat | Beef | unpacked | farmer’s market/street vendors | >500g | 2015-08-10 | Shaanxi | Tongchuan city | Riverbanks farmers market halal meat shop | Production | 2015.8.10 |
| SA238 | Raw meat | Mutton | unpacked | farmer’s market/street vendors | >500g | 2015-08-10 | Shaanxi | Tongchuan city | Riverbanks farmers market halal meat shop | Production | 2015.8.10 |
| SA239 | Raw meat | Beef | unpacked | farmer’s market/street vendors | >500g | 2015-08-10 | Shaanxi | Tongchuan city | Bridge farmers market waves, raw and cooked beef | Production | 2015.8.10 |
| SA240 | Raw meat | Duck | unpacked | farmer’s market/street vendors | >500g | 2015-11-02 | Shaanxi | Tongchuan city | Changfeng xinmiao seafood market chickens, ducks, fish wholesale Center | Production | 2015.11.2 |
| SA241 | Raw meat | Pork | unpacked | farmer’s market/street vendors | >500g | 2015-05-05 | Shaanxi | In weinan city | Southwest corner of the South pond, farmer's market fresh Santan | Homemade | 20150505 |
| SA242 | Raw meat | Pork | unpacked | farmer’s market/street vendors | >500g | 2015-05-05 | Shaanxi | In weinan city | Dawn binhe agricultural and sideline products markets Wang assured meat shop | Homemade | 20150505 |
| SA243 | Raw meat | Pork | unpacked | farmer’s market/street vendors | >500g | 2015-06-01 | Shaanxi | In weinan city | Day road farmers market shuanghui chilled meat shop | Owner made | 20150601 |
| SA244 | Raw meat | Mutton | unpacked | farmer’s market/street vendors | >500g | 2015-06-01 | Shaanxi | In weinan city | Qin LU, new live poultry meat shop | Owner made | 20150601 |
| SA245 | Raw meat | Mutton | unpacked | farmer’s market/street vendors | >500g | 2015-07-20 | Shaanxi | In weinan city | Self home the middle of the street meat shop | Store produce | 20150720 |
| SA246 | Raw meat | Beef | unpacked | farmer’s market/street vendors | >500g | 2015-07-20 | Shaanxi | In weinan city | Jie Xu Jia, meat shop | Store produce | 20150720 |
| SA247 | Raw meat | Beef | unpacked | farmer’s market/street vendors | >500g | 2015-07-20 | Shaanxi | In weinan city | Jie Zhong Duan halal meat shop | Baishui Xigu Town slaughterhouse | 20150720 |
| SA248 | Raw meat | Pork | unpacked | farmer’s market/street vendors | >500g | 2015-08-04 | Shaanxi | In weinan city | South Street farmer's market in Northwest ingredients greengrocer | Owner production | 20150804 |
| SA249 | Raw meat | Mutton | unpacked | farmer’s market/street vendors | >500g | 2015-08-04 | Shaanxi | In weinan city | Imam Street farmer's market in South Korea for beef and mutton shops | Owner production | 20150804 |
| SA250 | Raw meat | Duck | unpacked | Supermarket/department stores | >500g | 2015-08-04 | Shaanxi | In weinan city | Kingdom of the Golden Dragon Boulevard shopping mall | Supermarket self- | 20150804 |
| SA251 | Raw meat | Duck | unpacked | Supermarket/department stores | >500g | 2015-08-04 | Shaanxi | In weinan city | Kingdom of station Street West Shaanxi branch | Supermarket self- | 20150804 |
| SA252 | Raw meat | Duck | unpacked | farmer’s market/street vendors | >500g | 2015-08-04 | Shaanxi | In weinan city | Station Street stars aquatic vegetables seasoning shop | Owner made | 20150804 |
| SA253 | Raw meat | Beef | unpacked | farmer’s market/street vendors | >500g | 2015-08-17 | Shaanxi | In weinan city | North of Huan Cheng bei Jie, Li Feng of the market of beef and mutton and vegetables stores | Owner made | 20150817 |
| SA254 | Raw meat | Chicken | unpacked | farmer’s market/street vendors | >500g | 2015-08-17 | Shaanxi | In weinan city | Huan Cheng bei Jie city vegetable market 369 Aquatic feed | Huayin city farms | 20150816 |
| SA255 | Raw meat | Beef | unpacked | farmer’s market/street vendors | >500g | 2015-09-14 | Shaanxi | In weinan city | Guzhen town Fairgrounds, lane original beef and mutton shops | Owner production | 20150914 |
| SA256 | Raw meat | Beef | unpacked | farmer’s market/street vendors | >500g | 2015-09-14 | Shaanxi | In weinan city | Dong Huan Lu Xiang Yuan Lu Jia beef, farmers ' markets and old shops | Owner production | 20150914 |
| SA257 | Raw meat | Chicken | unpacked | farmer’s market/street vendors | >500g | 2015-09-14 | Shaanxi | In weinan city | Dong Huan Lu Xiang Yuan market meat flavored aquatic products wholesale Center, wide | Owner production | 20150913 |
| SA258 | Raw meat | Chicken | unpacked | farmer’s market/street vendors | >500g | 2015-09-14 | Shaanxi | In weinan city | Town lane market retail | Owner production | 20150913 |
| SA259 | Raw meat | Pork | unpacked | farmer’s market/street vendors | >500g | 2015-11-23 | Shaanxi | In weinan city | New road farmers market Wang butcher shop | Owner made | 20151123 |
| SA260 | Raw meat | Pork | unpacked | farmer’s market/street vendors | >500g | 2015-11-23 | Shaanxi | In weinan city | Pond Road in the South West of the farmers market Santan | Linwei district Hung-yun, weinan city slaughter plant | 20151123 |
| SA261 | Raw meat | Pork | unpacked | farmer’s market/street vendors | >500g | 2015-11-23 | Shaanxi | In weinan city | Northwest corner of South pond road farmers market Santan | Linwei district Hung-yun, weinan city slaughter plant | 20151123 |
| SA262 | Raw meat | Chicken | unpacked | farmer’s market/street vendors | >500g | 2015-11-23 | Shaanxi | In weinan city | New road north of farmers ' market Santan | Owner made | 20151123 |
| SA263 | Raw meat | Chicken | unpacked | farmer’s market/street vendors | >500g | 2015-11-23 | Shaanxi | In weinan city | Cangchenglu Wang, Nick farmer's market West of Santan | Owner made | 20151123 |
| SA264 | Raw meat | Pork | unpacked | Supermarket/department stores | >500g | 2015-05-11 | Shaanxi | XI ' an city | Fang Jie Zheng 364 More supermarket | Unknown | 2015-5-11 |
| SA265 | Raw meat | Mutton | unpacked | farmer’s market/street vendors | >500g | 2015-05-11 | Shaanxi | XI ' an city | Fang Yi Jie 260 Deer, Highland street market 2 Set Pan Jiawei mutton and beef wholesale Department | Unknown | 2015-5-11 |
| SA266 | Raw meat | Chicken | unpacked | farmer’s market/street vendors | >500g | 2015-05-18 | Shaanxi | XI ' an city | Zhangba road 16 Jane Finch farmers ' markets 1-3 Two Tigers aquatic product frozen goods | Unknown | 2015-5-18 |
| SA267 | Raw meat | Chicken | unpacked | farmer’s market/street vendors | >500g | 2015-05-25 | Shaanxi | XI ' an city | 2 Area road 2 East West, farmers ' markets a third niche fresh chickens live fish wholesale | Unknown | 20150525 |
| SA268 | Raw meat | Chicken | unpacked | Supermarket/department stores | >500g | 2015-06-01 | Shaanxi | XI ' an city | Peace villages southwest of cross everybody supermarket | Unknown | 20150601 |
| SA269 | Raw meat | Beef | unpacked | farmer’s market/street vendors | >500g | 2015-06-15 | Shaanxi | XI ' an city | Wen Wei Lu 101 Blind farmer's market West 31 Any meat shop | Unknown | 20150615 |
| SA270 | Raw meat | Chicken | unpacked | farmer’s market/street vendors | >500g | 2015-06-15 | Shaanxi | XI ' an city | Wen Wei Lu 101 Blind farmer's market, Eastern 14 Small chicken shop | Unknown | 20150615 |
| SA271 | Raw meat | Chicken | unpacked | farmer’s market/street vendors | >500g | 2015-07-27 | Shaanxi | XI ' an city | Fang Yi Jie 260 First shed deer North Highland street market, West row 1 | Unknown | 20150727 |
| SA272 | Raw meat | Beef | unpacked | farmer’s market/street vendors | >500g | 2015-07-27 | Shaanxi | XI ' an city | Fang Yi Jie 260 Deer, Highland street market 2 Set Pan Jiawei mutton and beef wholesale Department | Unknown | 20150727 |
| SA273 | Raw meat | Pork | unpacked | Supermarket/department stores | >500g | 2015-07-27 | Shaanxi | XI ' an city | Fang Jie Zheng 364 More supermarket | Unknown | 20150727 |
| SA274 | Raw meat | Duck | unpacked | farmer’s market/street vendors | >500g | 2015-08-03 | Shaanxi | XI ' an city | Xiyinglu 220 Wang Jia Cun Shi bei er fourth Wang Jiaxian chicken shop | Unknown | 20150803 |
| SA275 | Raw meat | Pork | unpacked | farmer’s market/street vendors | >500g | 2015-08-11 | Shaanxi | XI ' an city | 2 Area road 2 East side, West farmers ' market fourth China pure enthusiasm and sheep meat | Unknown | 20150811 |
| SA276 | Raw meat | Pork | unpacked | farmer’s market/street vendors | >500g | 2015-08-11 | Shaanxi | XI ' an city | Long cloud Tower square G-003 Mother take food | Unknown | 20150811 |
| SA277 | Raw meat | Mutton | unpacked | farmer’s market/street vendors | >500g | 2015-08-17 | Shaanxi | XI ' an city | Fu Dong village farmers market King second and sixth small farmer's distribution Department | Inner Mongolia little sheep seasoning food co | 20150706 |
| SA278 | Raw meat | Chicken | unpacked | farmer’s market/street vendors | >500g | 2015-08-17 | Shaanxi | XI ' an city | Fifth Jian Xiang Fu Dong village farmers ' markets and poultry shops | Unknown | 20150817 |
| SA279 | Raw meat | Chicken | unpacked | Supermarket/department stores | >500g | 2015-09-07 | Shaanxi | XI ' an city | Gaoling deer songs 59 Mart Shopping Plaza, | Unknown | 20150907 |
| SA280 | Raw meat | Pork | unpacked | farmer’s market/street vendors | >500g | 2015-09-14 | Shaanxi | XI ' an city | North Gate space six farmers ' markets first Fang Xin brand fresh pork store | Unknown | 20150914 |
| SA281 | Raw meat | Chicken | unpacked | farmer’s market/street vendors | >500g | 2015-10-12 | Shaanxi | XI ' an city | Fang Yi Jie 260 Deer, Highland street markets East of the first row of the first | Unknown | 20151012 |
| SA282 | Raw meat | Pork | unpacked | farmer’s market/street vendors | >500g | 2015-10-19 | Shaanxi | XI ' an city | Qujiang mercy West Road, Qin, Han and Tang Plaza C 2 Loudeshou Palace | Unknown | 20151019 |
| SA283 | Raw meat | Chicken | unpacked | farmer’s market/street vendors | >500g | 2015-10-26 | Shaanxi | XI ' an city | 2 Area road 2 East West, farmers ' markets North third (niche) fresh chicken fish wholesale | Unknown | 20151026 |
| SA284 | Raw meat | Mutton | unpacked | Supermarket/department stores | >500g | 2015-11-16 | Shaanxi | XI ' an city | Deer songs 60 Mart Shopping Plaza, | Unknown | 20151116 |
| SA285 | Raw meat | Mutton | unpacked | farmer’s market/street vendors | >500g | 2015-05-14 | Shaanxi | Xianyang city | Min yuan cross John mutton and beef wholesale Department | Shaanxi xianyang Institute of cross John mutton and beef wholesale Department | 20150514 |
| SA286 | Raw meat | Mutton | unpacked | farmer’s market/street vendors | >500g | 2015-05-14 | Shaanxi | Xianyang city | Qin Wei yang road farmers ' market | Shaanxi Xian Yang Weiyang Lu Qin farmers market | 20150514 |
| SA287 | Raw meat | Mutton | unpacked | Supermarket/department stores | >500g | 2015-05-14 | Shaanxi | Xianyang city | Wen Hui people man music store | Baoji city, Shengyuan food co | 20141203 |
| SA288 | Raw meat | Chicken | unpacked | farmer’s market/street vendors | >500g | 2015-10-13 | Shaanxi | Xianyang city | University farmers ' market where live poultry chicken shop | Unknown | 20151013 |
| SA289 | Raw meat | Duck | unpacked | farmer’s market/street vendors | >500g | 2015-10-13 | Shaanxi | Xianyang city | Min yuan yan four birds, farmers market butcher shop | Unknown | 20151013 |
| SA290 | Raw meat | Pork | unpacked | farmer’s market/street vendors | >500g | 2015-04-07 | Shaanxi | Yanan city | Qi Jia Xu Xiaobao Bay farmers ' markets | Zhang Lizi ditch slaughterhouse | 20150407 |
| SA291 | Raw meat | Mutton | unpacked | farmer’s market/street vendors | >500g | 2015-04-20 | Shaanxi | Yanan city | Jin Yuan lamb pork wholesale and retail | Wu Qi slaughterhouse | 20150420 |
| SA292 | Raw meat | Chicken | unpacked | farmer’s market/street vendors | >500g | 2015-04-20 | Shaanxi | Yanan city | Jin Yuan lamb pork wholesale and retail | Unknown | 20150221 |
| SA293 | Raw meat | Chicken | unpacked | farmer’s market/street vendors | >500g | 2015-04-20 | Shaanxi | Yanan city | Victory mountain Jin Yuan lamb pork market in wholesale and retail | Unknown | 20150230 |
| SA294 | Raw meat | Chicken | unpacked | farmer’s market/street vendors | >500g | 2015-04-21 | Shaanxi | Yanan city | Bridge market fish shop | Unknown | 20150402 |
| SA295 | Raw meat | Pork | unpacked | farmer’s market/street vendors | >500g | 2015-06-01 | Shaanxi | Yanan city | Sha Jie Shi Xiao-Wei Leung meat sales | Unknown | 20150601 |
| SA296 | Raw meat | Pork | unpacked | farmer’s market/street vendors | >500g | 2015-07-14 | Shaanxi | Yanan city | Rural agricultural products meat market area 33 | Ansai slaughterhouse | 20150714 |
| SA297 | Raw meat | Mutton | unpacked | farmer’s market/street vendors | >500g | 2015-09-15 | Shaanxi | Yanan city | Two Guo Li Zhuang Ke Mizoguchi meat Department | Kawaguchi, slaughterhouse | 20150915 |
| SA298 | Raw meat | Mutton | unpacked | farmer’s market/street vendors | >500g | 2015-09-28 | Shaanxi | Yanan city | ER DAO Li Yanxiong meat Street farmers ' markets | King Cha Gou slaughterhouse | 20150928 |
| SA299 | Raw meat | Chicken | unpacked | farmer’s market/street vendors | >500g | 2015-10-13 | Shaanxi | Yanan city | Liu Xinyan chicken next to farmers ' markets in the three live fish wholesale market | King Cha Gou slaughterhouse | 20151013 |
| SA300 | Raw meat | Duck | unpacked | Supermarket/department stores | >500g | 2015-10-13 | Shaanxi | Yanan city | Dongxing Parkway supermarket | Unknown | 20151006 |
| SA301 | Raw meat | Beef | unpacked | farmer’s market/street vendors | >500g | 2015-04-27 | Shaanxi | In Yulin city | Old town farmers market North East Gate 1 Integrity beef | Unknown | 20150427 |
| SA302 | Raw meat | Pork | unpacked | farmer’s market/street vendors | >500g | 2015-04-27 | Shaanxi | In Yulin city | North East loop farmers ' markets -5 Yokoyama mutton pork shop | Unknown | 20150427 |
| SA303 | Raw meat | Pork | unpacked | farmer’s market/street vendors | >500g | 2015-04-27 | Shaanxi | In Yulin city | County Road farmers market on East row 7 Assured, the butcher's | Unknown | 20150427 |
| SA304 | Raw meat | Pork | unpacked | farmer’s market/street vendors | >500g | 2015-04-27 | Shaanxi | In Yulin city | Yuyang sancha village of town bridge meat vegetables grocery supermarket | Unknown | 20150427 |
| SA305 | Raw meat | Pork | unpacked | farmer’s market/street vendors | >500g | 2015-05-05 | Shaanxi | In Yulin city | Jia Jun South Street farmer's market pork meat shop | Unknown | 20150505 |
| SA306 | Raw meat | Pork | unpacked | farmer’s market/street vendors | >500g | 2015-05-05 | Shaanxi | In Yulin city | Market lane integrity pork | Unknown | 20150505 |
| SA307 | Raw meat | Pork | unpacked | farmer’s market/street vendors | >500g | 2015-11-03 | Shaanxi | In Yulin city | Sha Qu wet market pork products 2 | Unknown | 20151103 |
| SA308 | Raw meat | Pork | unpacked | farmer’s market/street vendors | >500g | 2015-11-03 | Shaanxi | In Yulin city | NaN Guan farmers market 38 Guo Zhi, just us and UK meat shop | Unknown | 20151103 |
| SA309 | Raw meat | Chicken | unpacked | Supermarket/department stores | >500g | 2015-11-17 | Shaanxi | In Yulin city | One family shopping mall | Unknown | 20151117 |
| SA310 | Raw meat | Chicken | unpacked | farmer’s market/street vendors | >500g | 2015-11-17 | Shaanxi | In Yulin city | Yulin City poultry market area 11 Rural sales of live birds | Unknown | 20151117 |
| SA311 | Raw meat | Chicken | unpacked | farmer’s market/street vendors | >500g | 2015-11-17 | Shaanxi | In Yulin city | Yulin city market is full of fragrant spices aquatic products wholesale | Unknown | 20151117 |
| SA312 | Raw meat | Pork | unpacked | Supermarket/department stores | >500g | 2015-04-22 | Fujian | Fuzhou City | Tesco (Pu road) | Tesco (Pu road) round Sweet Shoppe | 20150422 |
| SA313 | Raw meat | Pork | unpacked | Supermarket/department stores | >500g | 2015-04-22 | Fujian | Fuzhou City | Tesco (Pu road) | Tesco (Pu road) guiyou Shoppe | 20150422 |
| SA314 | Raw meat | Chicken | unpacked | Supermarket/department stores | >500g | 2015-09-22 | Fujian | Ningde city | Crown supermarket (gutian supply store) | / | 20151117 |
| SA315 | Raw meat | Beef | unpacked | farmer’s market/street vendors | >500g | 2015-10-26 | Fujian | In Putian City | Overseas markets 15 | Unknown | 20151026 |
| SA316 | Raw meat | Duck | unpacked | Supermarket/department stores | >500g | 2015-04-07 | Fujian | In Xiamen City | Le du Hui Wuyuan Bay Mall | Xiamen Bao food trading limited | 20151026 |
| SA317 | Raw meat | Mutton | unpacked | Supermarket/department stores | >500g | 2015-10-26 | Fujian | Zhangzhou city | Wal-Mart stores (Mall Branch) | Unknown | 20151025 |
| SA318 | Raw meat | Duck | unpacked | farmer’s market/street vendors | >500g | 2015-08-03 | Fujian | In sanming city | Hakka Center farmers ' market poultry stalls | / | 20151026 |
| SA319 | Raw meat | Duck | unpacked | farmer’s market/street vendors | >500g | 2015-06-09 | Fujian | In sanming city | 51 bridge market poultry stalls | 51 bridge market poultry stalls | 20150609 |
| SA320 | Raw meat | Chicken | unpacked | farmer’s market/street vendors | >500g | 2015-08-03 | Fujian | In sanming city | Hakka Center farmers ' market poultry stalls | / | 20151026 |
| SA321 | Raw meat | Duck | unpacked | farmer’s market/street vendors | >500g | 2015-07-15 | Hunan | Xiangtan City | Yi Su he Zhen road intersection with Ginkgo yisuhe Fenghuang road farmers market meat or poultry 76 | Unknown | 20150715 |
| SA322 | Raw meat | Mutton | unpacked | farmer’s market/street vendors | >500g | 2015-10-26 | Hunan | Xiangtan City | Tang Xin Pu Jin Xiang textile City Avenue farmers market group of poultry | Unknown | 20151026 |
| SA323 | Raw meat | Chicken | unpacked | farmer’s market/street vendors | >500g | 2015-10-26 | Hunan | Xiangtan City | Tang Kam Lely live poultry in Hunan vegetable shop head office 13787329576 | Unknown | 20151026 |
| SA324 | Raw meat | Duck | unpacked | farmer’s market/street vendors | >500g | 2015-10-26 | Hunan | Xiangtan City | Dongfeng Dongfeng road Shandong jinluo meat farmers market outlet store 13973214416 Across from poultry stalls | Unknown | 20151026 |
| SA325 | Raw meat | Duck | unpacked | Supermarket/department stores | >500g | 2015-10-26 | Hunan | Xiangtan City | Fang Cheng Tang Pu road heart shop | Unknown | 20151026 |
| SA326 | Raw meat | Pork | unpacked | farmer’s market/street vendors | >500g | 2015-10-26 | Hunan | Xiangtan City | Construction junction ningxiang pork markets two branches 15907321319 | Unknown | 20151026 |
| SA327 | Raw meat | Pork | unpacked | farmer’s market/street vendors | >500g | 2015-10-26 | Hunan | Xiangtan City | Abundant green of South Street, the Eight Immortals bridge farm NO.18390229321 | Unknown | 20151026 |
| SA328 | Raw meat | Pork | unpacked | farmer’s market/street vendors | >500g | 2015-05-25 | Hunan | Yiyang city | Boss Liu Jinshan Gold and silver mountain accessible temporary market stalls | Unknown | 20150525 |
| SA329 | Raw meat | Pork | unpacked | farmer’s market/street vendors | >500g | 2015-05-25 | Hunan | Yiyang city | Jinshanlujiangjin Cao bosses, farmers ' market stalls | Unknown | 20150525 |
| SA330 | Raw meat | Pork | unpacked | farmer’s market/street vendors | >500g | 2015-05-25 | Hunan | Yiyang city | Yi feng antlers Park, Golden Hill Road farmers market monopoly | Unknown | 20150525 |
| SA331 | Raw meat | Chicken | unpacked | farmer’s market/street vendors | >500g | 2015-06-24 | Hunan | Yiyang city | Boss Liu Jinshan Gold farmers ' markets stalls | Unknown | 20150624 |
| SA332 | Raw meat | Chicken | unpacked | farmer’s market/street vendors | >500g | 2015-06-24 | Hunan | Yiyang city | Peach farmer's market peach road boss Wang pitch | Unknown | 20150624 |
| SA333 | Raw meat | Duck | unpacked | farmer’s market/street vendors | >500g | 2015-06-24 | Hunan | Yiyang city | Big peach, peach road farmers ' market | Unknown | 20150624 |
| SA334 | Raw meat | Pork | unpacked | farmer’s market/street vendors | >500g | 2015-07-06 | Hunan | Yiyang city | Qionghu East Commerce Street farmers ' market | Unknown | 20150706 |
| SA335 | Raw meat | Pork | unpacked | farmer’s market/street vendors | >500g | 2015-07-06 | Hunan | Yiyang city | Dongting road north of farmers ' markets | Unknown | 20150706 |
| SA336 | Raw meat | Beef | unpacked | farmer’s market/street vendors | >500g | 2015-07-06 | Hunan | Yiyang city | Dongting road north of farmers ' markets | Unknown | 20150706 |
| SA337 | Raw meat | Beef | unpacked | farmer’s market/street vendors | >500g | 2015-07-06 | Hunan | Yiyang city | Hector road vegetable wholesale | Unknown | 20150706 |
| SA338 | Raw meat | Pork | unpacked | Supermarket/department stores | >500g | 2015-07-06 | Hunan | Yiyang city | Xiu Feng Zhong Lu Guang-Jia shopping mall | Unknown | 20150706 |
| SA339 | Raw meat | Chicken | unpacked | farmer’s market/street vendors | >500g | 2015-09-21 | Hunan | Yiyang city | Zijiang Lu Zhenhua vegetable wholesale market Wu Jianwen cow chicken | Unknown | 20150921 |
| SA340 | Raw meat | Duck | unpacked | farmer’s market/street vendors | >500g | 2015-09-21 | Hunan | Yiyang city | Zijiang Lu Zhenhua Yin Lianghua poultry wholesale vegetable market | Unknown | 20150921 |
| SA341 | Raw meat | Duck | unpacked | farmer’s market/street vendors | >500g | 2015-09-21 | Hunan | Yiyang city | Binjiang Lu, Cui Lijun Tuan Chau market stalls | Unknown | 20150921 |
| SA342 | Raw meat | Beef | unpacked | farmer’s market/street vendors | >500g | 2015-10-08 | Hunan | Yiyang city | Wu Chun-Hui city farmers ' markets of beef | Unknown | 20151008 |
| SA343 | Raw meat | Pork | unpacked | Supermarket/department stores | >500g | 2015-06-01 | Hunan | In Yueyang city | North street-food village square | Unknown | 20150921 |
| SA344 | Raw meat | Pork | unpacked | farmer’s market/street vendors | >500g | 2015-06-01 | Hunan | In Yueyang city | Tao Lin Zhen Shi Annan road farmers ' market | Unknown | 20150921 |
| SA345 | Raw meat | Duck | unpacked | Supermarket/department stores | >500g | 2015-06-01 | Hunan | In Yueyang city | Nian Zhong Road RT supermarket | Unknown | 20150921 |
| SA346 | Raw meat | Duck | unpacked | farmer’s market/street vendors | >500g | 2015-08-24 | Hunan | In Yueyang city | TS South Gate market 8 Booth | Unknown | 20150921 |
| SA347 | Raw meat | Chicken | unpacked | farmer’s market/street vendors | >500g | 2015-08-24 | Hunan | In Yueyang city | North main street farmers market poultry, linxiang 1 Booth | Unknown | 20150921 |
| SA348 | Raw meat | Pork | unpacked | Supermarket/department stores | >500g | 2015-08-24 | Hunan | In Yongzhou city | Lengshuitan district Ling ling bei Lu BU BU Gao Chao Shun Tak shop | Unknown | 2015.08.24 |
| SA349 | Raw meat | Pork | unpacked | Supermarket/department stores | >500g | 2015-05-15 | Hunan | Zhangjiajie city | Yang Zhong Road Lotus supermarket (train station) | Unknown | 20150515 |
| SA350 | Raw meat | Chicken | unpacked | Supermarket/department stores | >500g | 2015-08-19 | Hunan | Zhangjiajie city | Li Yuan Zhen, sangzhi County, Heping road courtesy supermarket | Unknown | 20150819 |
| SA351 | Raw meat | Pork | unpacked | farmer’s market/street vendors | >500g | 2015-05-21 | Hunan | Xiangxi Tujia and Miao autonomous | West entrance farmers markets meat products 4 Booth | Unknown | 20150521 |
| SA352 | Raw meat | Pork | unpacked | farmer’s market/street vendors | >500g | 2015-05-21 | Hunan | Xiangxi Tujia and Miao autonomous | West entrance farmers markets meat products 5 Booth | Unknown | 20150521 |
| SA353 | Raw meat | Chicken | unpacked | farmer’s market/street vendors | >500g | 2015-08-25 | Hunan | Xiangxi Tujia and Miao autonomous | Bai Sha Lu Xin female farmers market poultry meat 1 Booth | Unknown | 20150825 |
| SA354 | Raw meat | Chicken | unpacked | farmer’s market/street vendors | >500g | 2015-05-04 | Shandong | Liaocheng city | Wang farmers ' market South 2 Pai Dong 1 Booth | Homemade | 20150504 |
| SA355 | Raw meat | Chicken | unpacked | farmer’s market/street vendors | >500g | 2015-05-04 | Shandong | Liaocheng city | Wang farmers ' market South 2 Pai Dong 2 Booth | Homemade | 20150504 |
| SA356 | Raw meat | Chicken | unpacked | farmer’s market/street vendors | >500g | 2015-05-18 | Shandong | In zaozhuang city | Street rain shanting integrated fruit wholesale market in Hong Kong cold meat | Unknown | 20150825 |
| SA357 | Raw meat | Chicken | unpacked | Supermarket/department stores | >500g | 2015-05-18 | Shandong | In zaozhuang city | Qing Tan Lu Guicheng Super Nova store | Unknown | 20150825 |
| SA358 | Raw meat | Duck | unpacked | farmer’s market/street vendors | >500g | 2015-05-21 | Shandong | Rizhao city | Zhengyang Road market frozen meat products | Shandong Taiko foods, Ltd | 20150825 |
| SA359 | Raw meat | Chicken | unpacked | farmer’s market/street vendors | >500g | 2015-06-02 | Shandong | Dezhou city | Center Street farmers ' market 12 Shop | Unknown | 20150921 |
| SA360 | Raw meat | Chicken | unpacked | farmer’s market/street vendors | >500g | 2015-06-02 | Shandong | Dezhou city | Dry City area 5 Building retail meat shops along the street | Unknown | 20150921 |
| SA361 | Raw meat | Chicken | unpacked | Supermarket/department stores | >500g | 2015-05-05 | Shandong | Dongying city | Estuarine commercial Street 38 Ginza supermarket | Watson Muslim meat company | 20150921 |
| SA362 | Raw meat | Chicken | unpacked | Supermarket/department stores | >500g | 2015-05-05 | Shandong | Dongying city | Park, guangrao city hongxing aquatic total dealership | Unknown | 20150921 |
| SA363 | Raw meat | Pork | unpacked | farmer’s market/street vendors | >500g | 2015-05-06 | Shandong | Dongying city | Kenli County transit trade city 15 Butcher's shop | Kenli County transit trade city 15 Butcher's shop | 20150921 |
| SA364 | Raw meat | Duck | unpacked | Supermarket/department stores | >500g | 2015-05-19 | Shandong | Weihai city | Family joy supermarket South Gou store | Xintai and all the guests that steady-state food co | 20150921 |
| SA365 | Raw meat | Mutton | unpacked | farmer’s market/street vendors | >500g | 2015-06-01 | Shandong | Zibo city | Zichuan Mu Wang aquatic product city F26 Store song Ling West Road, | Unknown | 20150921 |
| SA366 | Raw meat | Beef | unpacked | farmer’s market/street vendors | >500g | 2015-06-01 | Shandong | Zibo city | Zichuan Mu Wang aquatic product city booth 2 Song Ling West Road | Unknown | 20150921 |
| SA367 | Raw meat | Pork | unpacked | farmer’s market/street vendors | >500g | 2015-06-01 | Shandong | Zibo city | Zichuan Mu Wang Zhao stalls aquatic city song Ling West Road | Unknown | 20150921 |
| SA368 | Raw meat | Pork | unpacked | farmer’s market/street vendors | >500g | 2015-06-01 | Shandong | Zibo city | Zichuan Mu Wang aquatic product city booth 3 Song Ling West Road | Unknown | 20150921 |
| SA369 | Raw meat | Pork | unpacked | Supermarket/department stores | >500g | 2015-06-01 | Shandong | Zibo city | Zichuan yinzuo song Ling and Zhang Bolu road intersection | Unknown | 20150921 |
| SA370 | Raw meat | Pork | unpacked | Supermarket/department stores | >500g | 2015-06-01 | Shandong | Zibo city | Zichuan yinzuo song Ling and Zhang Bolu road intersection | Unknown | 20150921 |
| SA371 | Raw meat | Chicken | unpacked | farmer’s market/street vendors | >500g | 2015-06-01 | Shandong | Zibo city | Zichuan Mu Wang aquatic product city eviscerated chicken sales song Ling West Road | Unknown | 20150921 |
| SA372 | Raw meat | Chicken | unpacked | farmer’s market/street vendors | >500g | 2015-06-01 | Shandong | Zibo city | Zichuan Mu Wang aquatic product city booth 1 Song Ling West Road | Unknown | 20150921 |
| SA373 | Raw meat | Chicken | unpacked | farmer’s market/street vendors | >500g | 2015-06-01 | Shandong | Zibo city | Zichuan Mu Wang aquatic product city booth 1 Song Ling West Road | Unknown | 20150921 |
| SA374 | Raw meat | Mutton | unpacked | farmer’s market/street vendors | >500g | 2015-06-01 | Shandong | Of Taian city | Xiang Yang market, Shun he Dong Road, xintai city, Simon | Xintai city Xiangyang markets Ding Changjiang homemade | 20150819 |
| SA375 | Raw meat | Beef | unpacked | Supermarket/department stores | >500g | 2015-06-01 | Shandong | Of Taian city | Dongping County Dong DAO XI Shan Lu general merchandise supermarkets | Ominous | 20150819 |
| SA376 | Raw meat | Chicken | unpacked | Supermarket/department stores | >500g | 2015-06-01 | Shandong | Of Taian city | Tsing Wun Road, xintai city 895 Qingyun Wang, Dong Cheng branch | Ominous | 20150819 |
| SA377 | Raw meat | Chicken | unpacked | farmer’s market/street vendors | >500g | 2015-06-01 | Shandong | Of Taian city | Ningyang County North of closed markets, Wang Chih-kang wholesale Department | Ominous | 20150819 |
| SA378 | Raw meat | Duck | unpacked | farmer’s market/street vendors | >500g | 2015-06-01 | Shandong | Of Taian city | Ningyang County North relations marketing Liang ying wholesale Department | Ominous | 20150819 |
| SA379 | Raw meat | Duck | unpacked | Supermarket/department stores | >500g | 2015-06-01 | Shandong | Of Taian city | Dong ping XI Shan Lu Ru original Street Dongping County supermarkets | Unknown | 20150819 |
| SA380 | Raw meat | Duck | unpacked | Supermarket/department stores | >500g | 2015-06-01 | Shandong | Of Taian city | Dong ping XI Shan Lu Ru original Street Dongping County supermarkets | Unknown | 20150819 |
| SA381 | Raw meat | Pork | unpacked | Supermarket/department stores | >500g | 2015-06-09 | Shandong | Laiwu city | Friendship Street 23 Laiwu steel, Department store | Unknown | 20150819 |
| SA382 | Raw meat | Mutton | unpacked | farmer’s market/street vendors | >500g | 2015-06-09 | Shandong | Laiwu city | Honggou cherry Park Community farmers ' market | Unknown | 20150819 |
| SA383 | Raw meat | Pork | unpacked | farmer’s market/street vendors | >500g | 2015-06-09 | Shandong | Laiwu city | Wan Funan Lu Wu gardens farmers ' market | Unknown | 20150819 |
| SA384 | Raw meat | Mutton | unpacked | farmer’s market/street vendors | >500g | 2015-06-09 | Shandong | Laiwu city | Chang Shao Guan SI, South farmers market | Unknown | 20150819 |
| SA385 | Raw meat | Mutton | unpacked | farmer’s market/street vendors | >500g | 2015-06-09 | Shandong | Laiwu city | Wen Yang da 21 Horse fat, Grill | Unknown | 20150819 |
| SA386 | Raw meat | Mutton | unpacked | Supermarket/department stores | >500g | 2015-06-09 | Shandong | Laiwu city | Win mouda street of fengcheng West Street 108 Reputation building supermarkets | Unknown | 20150615 |
| SA387 | Raw meat | Duck | unpacked | farmer’s market/street vendors | >500g | 2015-06-23 | Shandong | Laiwu city | Yongxing road, city farmers ' market | Unknown | 20150615 |
| SA388 | Raw meat | Chicken | unpacked | farmer’s market/street vendors | >500g | 2015-06-23 | Shandong | Laiwu city | Honggou cherry Park Community farmers ' market | Unknown | 20150615 |
| SA389 | Raw meat | Chicken | unpacked | farmer’s market/street vendors | >500g | 2015-06-23 | Shandong | Laiwu city | Honggou cherry Park Community farmers ' market | Unknown | 20150615 |
| SA390 | Raw meat | Chicken | unpacked | farmer’s market/street vendors | >500g | 2015-06-23 | Shandong | Laiwu city | Wan Funan Lu Wu gardens farmers ' market | Unknown | 20150615 |
| SA391 | Raw meat | Duck | unpacked | Supermarket/department stores | >500g | 2015-06-23 | Shandong | Laiwu city | Win mouda street of fengcheng West Street 108 Reputation building supermarkets | Taishan and six food company | 20150615 |
| SA392 | Raw meat | Duck | unpacked | Supermarket/department stores | >500g | 2015-06-23 | Shandong | Laiwu city | North Garden Road 35 RT-Mart commercial limited company | Unknown | 20150615 |
| SA393 | Raw meat | Chicken | unpacked | Supermarket/department stores | >500g | 2015-06-23 | Shandong | Laiwu city | North Garden Road 35 RT-Mart commercial limited company | Unknown | 20150615 |
| SA394 | Raw meat | Duck | unpacked | farmer’s market/street vendors | >500g | 2015-07-06 | Shandong | Liaocheng city | Wanda West Canal Road complex shunfa aquatic product market | Shandong Fengxiang industrial limited | 20150615 |
| SA395 | Raw meat | Pork | unpacked | farmer’s market/street vendors | >500g | 2015-07-06 | Shandong | Liaocheng city | Qinghe Nan Jie market morning cold meat | Unknown | 20150615 |
| SA396 | Raw meat | Beef | unpacked | farmer’s market/street vendors | >500g | 2015-07-06 | Shandong | Liaocheng city | Qinghe Nan Jie market halal meat shop | Unknown | 20150615 |
| SA397 | Raw meat | Chicken | unpacked | farmer’s market/street vendors | >500g | 2015-08-04 | Shandong | Dezhou city | Middle Road, Desi in Wuzhou restaurant | Laiyang Jubilee foods limited | 20150615 |
| SA398 | Raw meat | Chicken | unpacked | farmer’s market/street vendors | >500g | 2015-08-04 | Shandong | Dezhou city | City cross street market 31 Live chicken wholesale | Unknown | 20150615 |
| SA399 | Raw meat | Duck | unpacked | farmer’s market/street vendors | >500g | 2015-08-04 | Shandong | Dezhou city | Yu Wang, City Road market 12 Old Peking duck restaurant | Unknown | 20150615 |
| SA400 | Raw meat | Beef | unpacked | farmer’s market/street vendors | >500g | 2015-09-07 | Shandong | Zibo city | Linzi align double park farmers ' market: road 18-12 Near North 2 Booth | Unknown | 20150615 |
| SA401 | Raw meat | Mutton | unpacked | farmer’s market/street vendors | >500g | 2015-09-07 | Shandong | Zibo city | Linzi align double park farmers ' market: road 18-12 Near South 7 Booth | Unknown | 20150615 |
| SA402 | Raw meat | Pork | unpacked | farmer’s market/street vendors | >500g | 2015-09-07 | Shandong | Zibo city | Linzi align double park farmers ' market: road 18-12 In the near 4 Booth | Unknown | 20150615 |
| SA403 | Raw meat | Pork | unpacked | farmer’s market/street vendors | >500g | 2015-09-07 | Shandong | Zibo city | Linzi align double park farmers ' market: road 18-12 In the near 1 Booth | Unknown | 20150615 |
| SA404 | Raw meat | Mutton | unpacked | Supermarket/department stores | >500g | 2015-09-07 | Shandong | Zibo city | Maoye Shung road in times square 63 | Unknown | 20150615 |
| SA405 | Raw meat | Duck | unpacked | farmer’s market/street vendors | >500g | 2015-09-07 | Shandong | Zibo city | Linzi align Park farmers ' market 582 Booth | Unknown | 20150615 |
| SA406 | Raw meat | Duck | unpacked | farmer’s market/street vendors | >500g | 2015-08-24 | Shandong | Liaocheng city | Zhenxing road, Daihatsu Aquatic Center market spices | Unknown | 20150615 |
| SA407 | Raw meat | Beef | unpacked | farmer’s market/street vendors | >500g | 2015-09-07 | Shandong | Liaocheng city | Chun ling of the market of beef and mutton, gulou East Road shop | Unknown | 20150615 |
| SA408 | Raw meat | Chicken | unpacked | farmer’s market/street vendors | >500g | 2015-09-08 | Shandong | Dezhou city | Cross street market 31 | Unknown | 20151026 |
| SA409 | Raw meat | Chicken | unpacked | farmer’s market/street vendors | >500g | 2015-09-08 | Shandong | Dezhou city | Yu the great convenience market 27 | Unknown | 20151026 |
| SA410 | Raw meat | Pork | unpacked | farmer’s market/street vendors | >500g | 2015-09-08 | Shandong | Dezhou city | Silver San Li Zhuang Cun 16 Hongying, grocery store | Unknown | 20151026 |
| SA411 | Raw meat | Beef | unpacked | farmer’s market/street vendors | >500g | 2015-09-08 | Shandong | Dezhou city | Ling Lu halal meat shops | Lingxian, Shandong meat co | 20151026 |
| SA412 | Raw meat | Chicken | unpacked | farmer’s market/street vendors | >500g | 2015-09-08 | Shandong | Dezhou city | Dong Guan Dong Guan market community hospital 10 | Unknown | 20151026 |
| SA413 | Raw meat | Pork | unpacked | farmer’s market/street vendors | >500g | 2015-09-08 | Shandong | Dezhou city | Cao Ji central square, 构词成分。 Park grills | Unknown | 20151026 |
| SA414 | Raw meat | Pork | unpacked | farmer’s market/street vendors | >500g | 2015-09-08 | Shandong | Dezhou city | Dong Guan Dong Guan market community hospital 13 Jincheng, a butcher's shop | Unknown | 20151026 |
| SA415 | Raw meat | Beef | unpacked | farmer’s market/street vendors | >500g | 2015-10-13 | Shandong | Dezhou city | South High Street North of yanmazhuang halal meat shops | Unknown | 20151026 |
| SA416 | Raw meat | Beef | unpacked | farmer’s market/street vendors | >500g | 2015-10-13 | Shandong | Dezhou city | Greenhouse in North High Street North of halal meat shop | Unknown | 20151026 |
| SA417 | Raw meat | Pork | unpacked | Supermarket/department stores | >500g | 2015-09-15 | Shandong | In Jining city | Lu, Hualian supermarket | Unknown | 20151026 |
| SA418 | Raw meat | Chicken | unpacked | farmer’s market/street vendors | >500g | 2015-09-15 | Shandong | In Jining city | SI Wang Xin River Road chicken live fish shop | Unknown | 20151026 |
| SA419 | Raw meat | Duck | unpacked | farmer’s market/street vendors | >500g | 2015-09-15 | Shandong | In Jining city | SI River Road farmers market crispy duck restaurant | Unknown | 20151026 |
| SA420 | Raw meat | Chicken | unpacked | farmer’s market/street vendors | >500g | 2015-10-12 | Shandong | In Jining city | City of bridges 108 Evergreen market 126 Li min, booth | Unknown | 20151026 |
| SA421 | Raw meat | Mutton | unpacked | Supermarket/department stores | >500g | 2015-10-12 | Shandong | Zibo city | Zhang bei road 1373 Credibility f, huantai building | Unknown | 20151026 |
| SA422 | Raw meat | Chicken | unpacked | farmer’s market/street vendors | >500g | 2015-11-02 | Ningxia | Zhongwei city | Sea town Lu Li chengfang, cattle and sheep meat | Unknown | 20151102 |
| SA423 | Raw meat | Chicken | unpacked | farmer’s market/street vendors | >500g | 2015-11-02 | Ningxia | Zhongwei city | Government Street South, South Gate Ma Xiulan vegetable market stalls | Unknown | 20151102 |
| SA424 | Raw meat | Pork | unpacked | farmer’s market/street vendors | >500g | 2015-10-26 | Ningxia | Zhongwei city | Fu XING Xiang Liu butcher shop | Unknown | 20151026 |
| SA425 | Raw meat | Pork | unpacked | farmer’s market/street vendors | >500g | 2015-06-15 | Ningxia | Zhongwei city | Gulou East Street bus station-South season pork, fresh agricultural products wholesale market in East Gate day evaluation hypermarket | Unknown | 20150615 |
| SA426 | Raw meat | Chicken | unpacked | farmer’s market/street vendors | >500g | 2015-05-11 | Ningxia | Zhongwei city | Christmas Nan Jie Liu Tian Hong Bao integrated wholesale market chicken fish wholesale and retail | Unknown | 20150511 |
| SA427 | Raw meat | Chicken | unpacked | farmer’s market/street vendors | >500g | 2015-05-11 | Ningxia | Zhongwei city | Christmas Nan Jie Hong Bao Xu da San-Huang chicken wholesale market shop | Unknown | 20150511 |
| SA428 | Raw meat | Chicken | unpacked | Supermarket/department stores | >500g | 2015-05-11 | Ningxia | Zhongwei city | Cross Street South and old South spring department store | Huimin County, Shandong province economic development zone | 20150425 |
| SA429 | Raw meat | Pork | unpacked | farmer’s market/street vendors | >500g | 2015-04-20 | Ningxia | Zhongwei city | Christmas Nan Jie Hong Bao Xu Yongfeng meat wholesale market the wholesale store | Unknown | 20150420 |
| SA430 | Raw meat | Pork | unpacked | Supermarket/department stores | >500g | 2015-04-20 | Ningxia | Zhongwei city | Peace Street Blum Mall | Zhongning medlar student cooperative association | 20150420 |
| SA431 | Raw meat | Chicken | unpacked | farmer’s market/street vendors | >500g | 2015-10-26 | Ningxia | Wuzhong city | Jian Ming Street 22 Jin hua chicken shop | Golden Chicken shop | 2015.10.26 |
| SA432 | Raw meat | Duck | unpacked | Supermarket/department stores | >500g | 2015-05-18 | Ningxia | Shizuishan | Helan mountain North Road baide supermarket | Lu Xin, Shandong Yanggu electric six and foods limited | 21050510 |
| SA433 | Raw meat | Mutton | unpacked | farmer’s market/street vendors | >500g | 2015-05-12 | Ningxia | Yinchuan city | Regent Street, North Central wholesale market 52 Wang, four-meat shop | Unknown | 20150512 |
| SA434 | Raw meat | Chicken | unpacked | Supermarket/department stores | >500g | 2015-10-08 | Shanghai | Yangpu district | Century and of Lianhua road (State Road 753 Number) | Unknown | 15-10-8 |
| SA435 | Raw meat | Chicken | unpacked | Supermarket/department stores | >500g | 2015-11-03 | Shanghai | Yangpu district | Tesco (Tang Shan Road 1018 , Ground floor, first floor) | Unknown | 15-11-3 |
| SA436 | Raw meat | Pork | unpacked | farmer’s market/street vendors | >500g | 2015-08-11 | Shanghai | Qingpu district | Chen Chunfang xiayang Lake farmers ' markets - Huaqing road, Qingpu district, 515 | Unknown | 20150811 |
| SA437 | Raw meat | Beef | unpacked | farmer’s market/street vendors | >500g | 2015-04-20 | Shanghai | Yangpu district | Vegetable shuangyang road, range road 419 | Booth 308 | 2015/4/20 |
| SA438 | Raw meat | Beef | unpacked | farmer’s market/street vendors | >500g | 2015-11-03 | Shanghai | Yangpu district | Huoshan road food market (Huoshan road 1118 Number) booth 222 | Unknown | 15-11-3 |
| SA439 | Raw meat | Duck | unpacked | farmer’s market/street vendors | >500g | 2015-09-01 | Shanghai | Yangpu district | Guo Lu CAI Chang (Zhong Yuan lu 209 Number) booth 239 | Unknown | 15-9-1 |
| SA440 | Raw meat | Pork | unpacked | Supermarket/department stores | >500g | 2015-10-08 | Shanghai | Yangpu district | Century and of Lianhua road (State Road 753 Number) | Unknown | 15-10-8 |
| SA441 | Raw meat | Chicken | unpacked | farmer’s market/street vendors | >500g | 2015-11-03 | Shanghai | Yangpu district | Huoshan road food market (Huoshan road 1118 Number) booth 226 | Beijing century Liyuan ecological agriculture co | 15-6-1 |
| SA442 | Raw meat | Chicken | unpacked | farmer’s market/street vendors | >500g | 2015-09-08 | Shanghai | Qingpu district | Zhao Xiang, the farmers ' market E-2- Zhao Zhong Road, Qingpu district, 77 | Unknown | 20150908 |
| SA443 | Raw meat | Pork | unpacked | farmer’s market/street vendors | >500g | 2015-05-11 | Shanghai | Yangpu district | Pujingyu Yang road, Shanghai market (Jing Yu Nan lu 78 Number) booth 345 | Pujingyu Yang road, Shanghai market (Jing Yu Nan lu 78 Number) booth 345 | 2015/5/11 |
| SA444 | Raw meat | Beef | unpacked | Supermarket/department stores | >500g | 2015-11-03 | Shanghai | Yangpu district | Tesco (Tang Shan Road 1018 , Ground floor, first floor) | TESCO Tesco, Dalian, Kunming store homemade | 15-11-3 |
| SA445 | Raw meat | Mutton | unpacked | Supermarket/department stores | >500g | 2015-04-07 | Shanghai | Qingpu district | NGS supermarket zhujiajiao store - Cao Ping Road, Qingpu district, 26 | Shanghai really foods limited | 14-12-14 |
| SA446 | Raw meat | Duck | unpacked | farmer’s market/street vendors | >500g | 2015-10-08 | Shanghai | Yangpu district | Dunhua road farms (dunhua road 168 P) booth 203 | Unknown | 15-10-8 |
| SA447 | Raw meat | Beef | unpacked | farmer’s market/street vendors | >500g | 2015-04-13 | Shanghai | PuTuo district | Wind market meats 1 Booth - Daduhe road 845 | Unknown | 20150914 |
| SA448 | Raw meat | Chicken | unpacked | farmer’s market/street vendors | >500g | 2015-06-09 | Shanghai | PuTuo district | Huachi market A7-6 Booth - Chi Lu 51 | Unknown | 15-6-9 |
| SA449 | Raw meat | Duck | unpacked | farmer’s market/street vendors | >500g | 2015-07-06 | Shanghai | Qingpu district | Three rivers market Chen Yanjun - West Town Road, Qingpu district, 258 | Unknown | 20150706 |
| SA450 | Raw meat | Chicken | unpacked | farmer’s market/street vendors | >500g | 2015-09-08 | Shanghai | Qingpu district | Zhao Xiang, the farmers ' market E-1- Zhao Zhong Road, Qingpu district, 77 | Unknown | 20150908 |
| SA451 | Raw meat | Chicken | unpacked | Supermarket/department stores | >500g | 2015-05-11 | Shanghai | Yangpu district | RT-Mart supermarket in Shanghai (huangxing road 1616 Number) | Shanghai six strong foods, Ltd | 2015/5/11 |
| SA452 | Raw meat | Pork | unpacked | farmer’s market/street vendors | >500g | 2015-09-01 | Shanghai | Yangpu district | Guo Lu CAI Chang (Zhong Yuan lu 209 Number) booth 212 | Unknown | 15-9-1 |
| SA453 | Raw meat | Chicken | unpacked | farmer’s market/street vendors | >500g | 2015-09-01 | Shanghai | Yangpu district | Guo Lu CAI Chang (Zhong Yuan lu 209 Number) booth 239 | Unknown | 15-9-1 |
| SA454 | Raw meat | Pork | unpacked | farmer’s market/street vendors | >500g | 2015-10-08 | Shanghai | Yangpu district | Dunhua road farms (dunhua road 168 P) booth 222 | Unknown | 15-10-8 |
| SA455 | Raw meat | Chicken | unpacked | farmer’s market/street vendors | >500g | 2015-09-14 | Shanghai | Fengxian district | Zhuang farmers market 65 Booth, buyun, zhuanghang Town Road, Fengxian district, Shanghai City 151 | Unknown | 20150914 |
| SA456 | Raw meat | Chicken | unpacked | farmer’s market/street vendors | >500g | 2015-05-11 | Shanghai | Yangpu district | Pujingyu Yang road, Shanghai market (Jing Yu Nan lu 78 Number) booth 343 | Pujingyu Yang road, Shanghai market (Jing Yu Nan lu 78 Number) booth 343 | 2015/5/11 |
| SA457 | Raw meat | Mutton | unpacked | Supermarket/department stores | >500g | 2015-09-01 | Shanghai | Yangpu district | Shop Wal-Mart wujiaochang (songhu road 125 2 Building) | Unknown | 15-8-29 |
| SA458 | Raw meat | Beef | unpacked | Supermarket/department stores | >500g | 2015-04-20 | Guangdong | Shanwei city | Sky Plaza, Station Road | Unknown | 15-4-20 |
| SA459 | Raw meat | Mutton | unpacked | farmer’s market/street vendors | >500g | 2015-04-21 | Guangdong | In Qingyuan city | Lianjiang road triangle market in first gear | Unknown | 20150421 |
| SA460 | Raw meat | Chicken | unpacked | farmer’s market/street vendors | >500g | 2015-05-13 | Guangdong | Foshan City | Tracker three birds stalls in Heilongjiang province 1 | Unknown | 15-5-13 |
| SA461 | Raw meat | Pork | unpacked | farmer’s market/street vendors | >500g | 2015-05-19 | Guangdong | Foshan City | Temple Street South Avenue in xianju | Unknown | 20150519 |
| SA462 | Raw meat | Pork | unpacked | farmer’s market/street vendors | >500g | 2015-05-20 | Guangdong | Foshan City | Lion City Road, shishan town meat market 1 Row 7 | Unknown | 20150520 |
| SA463 | Raw meat | Chicken | unpacked | farmer’s market/street vendors | >500g | 2015-05-25 | Guangdong | Shaoguan city | Xiongzhou road, prosperous farmers market | Unknown | 20150525 |
| SA464 | Raw meat | Duck | unpacked | farmer’s market/street vendors | >500g | 2015-05-25 | Guangdong | Yunfu city | Central market of the city 1 Wu Di, duck | Ominous | 2015.5.25 |
| SA465 | Raw meat | Chicken | unpacked | farmer’s market/street vendors | >500g | 2015-05-25 | Guangdong | Yunfu city | Love Ho linxi road supermarket | Ominous | 2015.5.25 |
| SA466 | Raw meat | Duck | unpacked | farmer’s market/street vendors | >500g | 2015-05-25 | Guangdong | Yunfu city | Li Guilin, Jiefang West Road chicken stalls | Ominous | 2015.5.25 |
| SA467 | Raw meat | Duck | unpacked | farmer’s market/street vendors | >500g | 2015-05-25 | Guangdong | In Meizhou city | China and Hong Kong Street, overseas Chinese town meat market good shops three | Unknown | 15-5-25 |
| SA468 | Raw meat | Duck | unpacked | farmer’s market/street vendors | >500g | 2015-05-28 | Guangdong | In Zhanjiang city | Li red meiluzhen West Renmin Road Central market stalls | Unknown | 20150528 |
| SA469 | Raw meat | Chicken | unpacked | farmer’s market/street vendors | >500g | 2015-05-26 | Guangdong | Shenzhen | South new road 2008 Namsan market 17 | Unknown | 20150526 |
| SA470 | Raw meat | Chicken | unpacked | farmer’s market/street vendors | >500g | 2015-05-26 | Guangdong | Shenzhen | South new road 2008 Namsan market 17 | Unknown | 20150526 |
| SA471 | Raw meat | Chicken | unpacked | Supermarket/department stores | >500g | 2015-05-26 | Guangdong | Shenzhen | Jinhai road, xixiang Street, Wal-Mart | Unknown | 20150526 |
| SA472 | Raw meat | Chicken | unpacked | Supermarket/department stores | >500g | 2015-05-28 | Guangdong | Jiangmen city | Revitalization of the road 30 CRV (new store) | Unknown | 15-5-23 |
| SA473 | Raw meat | Pork | unpacked | farmer’s market/street vendors | >500g | 2015-05-08 | Guangdong | Jieyang city | Hiu Tsui Road, dongsheng Street New River farmers market | / | 15-5-8 |
| SA474 | Raw meat | Chicken | unpacked | farmer’s market/street vendors | >500g | 2015-05-12 | Guangdong | Jieyang city | New River Road, dongsheng Street Mizoguchi market | / | 15-5-12 |
| SA475 | Raw meat | Chicken | unpacked | farmer’s market/street vendors | >500g | 2015-05-12 | Guangdong | Jieyang city | Quicksand quicksand quicksand Avenue East of the street market | / | 15-5-12 |
| SA476 | Raw meat | Pork | unpacked | farmer’s market/street vendors | >500g | 2015-06-08 | Guangdong | In Qingyuan city | Lianjiang road triangle market Li Guirong | Unknown | 20150608 |
| SA477 | Raw meat | Beef | unpacked | farmer’s market/street vendors | >500g | 2015-06-08 | Guangdong | In Qingyuan city | Lianjiang road triangle market Zhang Ziming | Unknown | 20150608 |
| SA478 | Raw meat | Pork | unpacked | farmer’s market/street vendors | >500g | 2015-06-08 | Guangdong | In Qingyuan city | German star Street, star market 6 | Unknown | 20150608 |
| SA479 | Raw meat | Pork | unpacked | Supermarket/department stores | >500g | 2015-06-08 | Guangdong | In Qingyuan city | Loop the new one better supermarkets | Unknown | 20150608 |
| SA480 | Raw meat | Chicken | unpacked | Supermarket/department stores | >500g | 2015-06-08 | Guangdong | In Qingyuan city | Loop the new one better supermarkets | Unknown | 20150608 |
| SA481 | Raw meat | Chicken | unpacked | farmer’s market/street vendors | >500g | 2015-06-08 | Guangdong | In Qingyuan city | German star Street, star market authentic Qingyuan chicken | Unknown | 20150608 |
| SA482 | Raw meat | Chicken | unpacked | farmer’s market/street vendors | >500g | 2015-06-04 | Guangdong | In Maoming city | Dong Lake Road East market of live chicken stalls | Unknown | 15-6-4 |
| SA483 | Raw meat | Duck | unpacked | farmer’s market/street vendors | >500g | 2015-06-09 | Guangdong | In Maoming city | High-tech Road East Choi Yi chicken duck | Unknown | 15-6-9 |
| SA484 | Raw meat | Chicken | unpacked | farmer’s market/street vendors | >500g | 2015-06-09 | Guangdong | In Maoming city | ChangPo visage of Wu Xing Lu Xinshi town line a long roof sloping comprehensive market chicken stalls | Unknown | 15-6-9 |
| SA485 | Raw meat | Pork | unpacked | Supermarket/department stores | >500g | 2015-06-10 | Guangdong | In Maoming city | Renmin South Road 2-8 Lake supermarket head offices | Unknown | 15-6-10 |
| SA486 | Raw meat | Beef | unpacked | farmer’s market/street vendors | >500g | 2015-06-10 | Guangdong | In Maoming city | Aunt vinyl shuangshan five road market beef | Unknown | 15-6-10 |
| SA487 | Raw meat | Pork | unpacked | farmer’s market/street vendors | >500g | 2015-06-03 | Guangdong | In Maoming city | Guan Qiao Zhen XING Lu XING long snack bar | Unknown | 15-6-3 |
| SA488 | Raw meat | Chicken | unpacked | Supermarket/department stores | >500g | 2015-07-14 | Guangdong | Foshan City | Daliang way too Gen 11 Tupperware store | Unknown | 15-7-14 |
| SA489 | Raw meat | Chicken | unpacked | Supermarket/department stores | >500g | 2015-07-09 | Guangdong | In Maoming city | Dong Dong Hu road, Wan Shan Plaza Mart supermarket | Unknown | 20150608 |
| SA490 | Raw meat | Pork | unpacked | Supermarket/department stores | >500g | 2015-07-06 | Guangdong | In Maoming city | Renmin South Road, Lai Crystal supermarket | Unknown | 15-7-6 |
| SA491 | Raw meat | Beef | unpacked | farmer’s market/street vendors | >500g | 2015-07-06 | Guangdong | In Maoming city | Oil city of six emerging markets B17 | Unknown | 15-7-6 |
| SA492 | Raw meat | Mutton | unpacked | farmer’s market/street vendors | >500g | 2015-07-06 | Guangdong | In Maoming city | Meter road, East of the market B38 | Unknown | 15-7-6 |
| SA493 | Raw meat | Chicken | unpacked | farmer’s market/street vendors | >500g | 2015-05-20 | Guangdong | Zhongshan city | Eastern xingwenluxingwen markets 10 | Unknown | 20150520 |
| SA494 | Raw meat | Chicken | unpacked | farmer’s market/street vendors | >500g | 2015-08-10 | Guangdong | Zhaoqing city | Lian Lian Mai Zhen Lian Mai market | Lian Lian Mai Zhen Lian Mai market | 20150809 |
| SA495 | Raw meat | Pork | unpacked | farmer’s market/street vendors | >500g | 2015-08-10 | Guangdong | Zhaoqing city | Huaicheng Yanjiang road, Riverside market | Unknown | 20150809 |
| SA496 | Raw meat | Pork | unpacked | farmer’s market/street vendors | >500g | 2015-08-10 | Guangdong | Zhaoqing city | Lian Lian Mai Zhen Lian Mai market home cuisine food | Home food food | 20150809 |
| SA497 | Raw meat | Pork | unpacked | farmer’s market/street vendors | >500g | 2015-07-22 | Guangdong | Jiangmen city | Lu Jiang highway bridge in the East China Sea side of Mong Kok food | Unknown | 15-7-22 |
| SA498 | Raw meat | Beef | unpacked | Supermarket/department stores | >500g | 2015-09-15 | Guangdong | Jiangmen city | East Gate Plaza, enping CRV (town Branch) | Unknown | 20150915 |
| SA499 | Raw meat | Chicken | unpacked | farmer’s market/street vendors | >500g | 2015-05-05 | Sichuan | In Nanchong city | Wang Zhen Nong Mao Shi Chang, Wang Zhongwei booth | No | 20150915 |
| SA500 | Raw meat | Chicken | unpacked | farmer’s market/street vendors | >500g | 2015-05-06 | Sichuan | In Nanchong city | Wu Chunrong que town farmers ' markets stalls | No | 20150915 |
| SA501 | Raw meat | Chicken | unpacked | farmer’s market/street vendors | >500g | 2015-05-08 | Sichuan | In Nanchong city | Wang Xiaofeng Li Jia Zhen farmers ' markets stalls | No | 20150915 |
| SA502 | Raw meat | Duck | unpacked | Supermarket/department stores | >500g | 2015-05-11 | Sichuan | In Nanchong city | Hontex Avenue baixin supermarket | No | 20150915 |
| SA503 | Raw meat | Chicken | unpacked | farmer’s market/street vendors | >500g | 2015-05-11 | Sichuan | In Nanchong city | Liu Yanan Road North market stalls | No | 2015.5.11 |
| SA504 | Raw meat | Chicken | unpacked | farmer’s market/street vendors | >500g | 2015-06-09 | Sichuan | Meishan city | Dongpo district East of the city farmers ' market 08 Soong poultry stalls, stalls | / | 2015-06-09 |
| SA505 | Raw meat | Chicken | unpacked | farmer’s market/street vendors | >500g | 2015-04-13 | Sichuan | Yibin city | Xilai xuefu road kitchen restaurant | Unknown | 2015.4.12 |
| SA506 | Raw meat | Pork | unpacked | Supermarket/department stores | >500g | 2015-07-02 | Sichuan | In Guangyuan city | Cangxi County, East Street 12# Hualian supermarket | Cangxi County Hongyu slaughter | 2015-06-09 |
| SA507 | Raw meat | Pork | unpacked | Supermarket/department stores | >500g | 2015-07-03 | Sichuan | In Guangyuan city | Zhao Qing road 5# Barn fresh food supermarket | Golden foods limited | 2015-06-09 |
| SA508 | Raw meat | Chicken | unpacked | farmer’s market/street vendors | >500g | 2015-07-03 | Sichuan | In Nanchong city | Tsao lidu town farmers ' markets frozen wholesale | No | 2015-06-09 |
| SA509 | Raw meat | Duck | unpacked | farmer’s market/street vendors | >500g | 2015-07-06 | Sichuan | In Nanchong city | Nan lu, South of eating fresh market refrigerated | No | 2015-06-09 |
| SA510 | Raw meat | Duck | unpacked | farmer’s market/street vendors | >500g | 2015-07-08 | Sichuan | In Nanchong city | Dong Guan Zhen Nong Mao Shi Chang, dongqiang frozen | No | 2015-06-09 |
| SA511 | Raw meat | Chicken | unpacked | farmer’s market/street vendors | >500g | 2015-07-06 | Sichuan | Ziyang city | Zhao Xianhua Dragon market stalls | Production | 2015-06-09 |
| SA512 | Raw meat | Chicken | unpacked | farmer’s market/street vendors | >500g | 2015-07-06 | Sichuan | Ziyang city | Zhao Xianhua Dragon market stalls | Production | 20150706 |
| SA513 | Raw meat | Duck | unpacked | farmer’s market/street vendors | >500g | 2015-07-06 | Sichuan | Ziyang city | Zhao Xianhua Dragon market stalls | Production | 20150706 |
| SA514 | Raw meat | Chicken | unpacked | farmer’s market/street vendors | >500g | 2015-07-06 | Sichuan | Ziyang city | Zhao Xianhua Dragon market stalls | Production | 20150706 |
| SA515 | Raw meat | Beef | unpacked | farmer’s market/street vendors | >500g | 2015-07-06 | Sichuan | Ziyang city | Zhao Xianhua Dragon market stalls | Production | 20150706 |
| SA516 | Raw meat | Duck | unpacked | farmer’s market/street vendors | >500g | 2015-07-06 | Sichuan | Ziyang city | Zhao Xianhua Dragon market stalls | Production | 20150706 |
| SA517 | Raw meat | Chicken | unpacked | farmer’s market/street vendors | >500g | 2015-07-06 | Sichuan | Ziyang city | Huanglong Rayong, market stalls | Production | 20150706 |
| SA518 | Raw meat | Chicken | unpacked | farmer’s market/street vendors | >500g | 2015-07-06 | Sichuan | Ziyang city | Huanglong Rayong, market stalls | Production | 20150706 |
| SA519 | Raw meat | Chicken | unpacked | farmer’s market/street vendors | >500g | 2015-07-06 | Sichuan | Ziyang city | Huanglong Rayong, market stalls | Production | 20150706 |
| SA520 | Raw meat | Duck | unpacked | farmer’s market/street vendors | >500g | 2015-07-06 | Sichuan | Ziyang city | Huanglong Rayong, market stalls | Production | 20150706 |
| SA521 | Raw meat | Chicken | unpacked | farmer’s market/street vendors | >500g | 2015-07-06 | Sichuan | Ziyang city | Huanglong Rayong, market stalls | Production | 20150706 |
| SA522 | Raw meat | Duck | unpacked | farmer’s market/street vendors | >500g | 2015-07-06 | Sichuan | Ziyang city | Dragon marketing Kim booth | Production | 20150706 |
| SA523 | Raw meat | Chicken | unpacked | farmer’s market/street vendors | >500g | 2015-07-06 | Sichuan | Ziyang city | Dragon marketing Kim booth | Production | 20150706 |
| SA524 | Raw meat | Chicken | unpacked | farmer’s market/street vendors | >500g | 2015-07-06 | Sichuan | Ziyang city | Dragon marketing Kim booth | Production | 20150706 |
| SA525 | Raw meat | Mutton | unpacked | Supermarket/department stores | >500g | 2015-08-19 | Sichuan | Bazhong city | East Street 129 Teck Whye baixin supermarket | Inner Mongolia o Phillips foods, Ltd | 20150626 |
| SA526 | Raw meat | Chicken | unpacked | farmer’s market/street vendors | >500g | 2015-07-27 | Sichuan | Yibin city | Farmers ' market on the wenjiangzhen Yan Wei mobile stalls | Sellers made | 2015-07-27 |
| SA527 | Raw meat | Duck | unpacked | farmer’s market/street vendors | >500g | 2015-09-14 | Sichuan | Ziyang city | Red West market poultry, seafood, frozen food business Department | Production | 20150914 |
| SA528 | Raw meat | Duck | unpacked | farmer’s market/street vendors | >500g | 2015-09-14 | Sichuan | Ziyang city | Zhao Xianhua Dragon market stalls | Production | 20150914 |
| SA529 | Raw meat | Chicken | unpacked | farmer’s market/street vendors | >500g | 2015-09-14 | Sichuan | Ziyang city | Zhao Xianhua Dragon market stalls | Production | 20150914 |
| SA530 | Raw meat | Duck | unpacked | farmer’s market/street vendors | >500g | 2015-09-14 | Sichuan | Ziyang city | Zhao Xianhua Dragon market stalls | Production | 20150914 |
| SA531 | Raw meat | Pork | unpacked | farmer’s market/street vendors | >500g | 2015-09-07 | Sichuan | Panzhihua city | Panzhihua nine six markets in the Eastern Avenue 812 | / | 2015-07-27 |
| SA532 | Raw meat | Pork | unpacked | farmer’s market/street vendors | >500g | 2015-10-27 | Sichuan | Panzhihua city | Benevolence and old farmers ' market 2-056 | / | 2015-07-27 |
| SA533 | Raw meat | Duck | unpacked | Supermarket/department stores | >500g | 2015-11-09 | Sichuan | In Nanchong city | Yingbin Avenue, billion hundred shopping malls | No | 2015-07-27 |
| SA534 | Raw meat | Chicken | unpacked | Supermarket/department stores | >500g | 2015-11-09 | Sichuan | In Nanchong city | RT-GUI open road supermarket | No | 2015-07-27 |
| SA535 | Raw meat | Chicken | unpacked | Supermarket/department stores | >500g | 2015-11-10 | Sichuan | In Nanchong city | Jincheng road Mart supermarket | No | 2015-07-27 |
| SA536 | Raw meat | Chicken | unpacked | farmer’s market/street vendors | >500g | 2015-11-10 | Sichuan | In Nanchong city | Hong Fu to little street market shop | No | 2015-07-27 |
| SA537 | Raw meat | Chicken | unpacked | farmer’s market/street vendors | >500g | 2015-11-12 | Sichuan | Guang ' an city | Dingpingzhen the ancient dragon and Jin Cheng Xinyuan o Avenue, linshui 1 1 Lou 42 、 43 | Dingpingzhen the ancient dragon and Jin Cheng Xinyuan o Avenue, linshui 1 1 Lou 42 、 43 | 20151112 |
| SA538 | Raw meat | Chicken | unpacked | farmer’s market/street vendors | >500g | 2015-05-28 | Heilongjiang | Hegang city | Coslight zero-km road farmers ' market | Unknown | 2015.05.28 |
| SA539 | Raw meat | Mutton | unpacked | Supermarket/department stores | >500g | 2015-10-16 | Heilongjiang | Daqing City | New Dongfeng village by Liu Jie shopping mall new supermarket lamb 1 Stand | Unknown | 20151016 |
| SA540 | Raw meat | Duck | unpacked | Supermarket/department stores | >500g | 2015-06-12 | Heilongjiang | Daqing City | Jing Qi Jie Qu Qing Ke long split chicken | Dragon slaughter | 20150612 |
| SA541 | Raw meat | Chicken | unpacked | Supermarket/department stores | >500g | 2015-06-12 | Heilongjiang | Daqing City | Jing Qi Jie Qu Qing Ke long split chicken | Dragon slaughter | 20150612 |
| SA542 | Raw meat | Chicken | unpacked | Supermarket/department stores | >500g | 2015-09-16 | Heilongjiang | Daqing City | Moore Street, fashionable international shopping supermarket 6 Number beds | Unknown | 20150916 |
| SA543 | Raw meat | Chicken | unpacked | Supermarket/department stores | >500g | 2015-09-16 | Heilongjiang | Daqing City | Moore Street, fashionable international shopping supermarket 6 Number beds | Unknown | 20150916 |
| SA544 | Raw meat | Chicken | unpacked | farmer’s market/street vendors | >500g | 2015-10-16 | Heilongjiang | Daqing City | Village nine wholesale market chicken 06 | Unknown | 20151016 |
| SA545 | Raw meat | Beef | unpacked | farmer’s market/street vendors | >500g | 2015-10-19 | Heilongjiang | Da hinggan Ling | Liu Ying industrial farmers ' markets for beef and mutton Renmin road monopoly | Jiagedaqi district Henan slaughter | 2015.10.19 |
| SA546 | Raw meat | Chicken | unpacked | Supermarket/department stores | >500g | 2015-06-08 | Heilongjiang | Da hinggan Ling | Chaoyang road, shenglong supermarket 2 Butcher's shop | Jiagedaqi district old crossing farmers | 2015.6.08 |
| SA547 | Raw meat | Chicken | unpacked | farmer’s market/street vendors | >500g | 2015-10-19 | Heilongjiang | Da hinggan Ling | Chaoyang Lu Jiawang farmers market 4 Butcher Shoppe | Jiagedaqi district old crossing farmers | 2015.10.19 |
| SA548 | Raw meat | Chicken | unpacked | Supermarket/department stores | >500g | 2015-10-19 | Heilongjiang | Da hinggan Ling | Renmin road, full of good supermarkets 3 Butcher Shoppe | Jiagedaqi district 64 Km of farmers | 2015.10.19 |
| SA549 | Raw meat | Beef | unpacked | farmer’s market/street vendors | >500g | 2015-05-27 | Heilongjiang | Heihe city | XING ' an Street 41-13 Liu Hongjun, Shuang-Fu market beef stand | Liu Hongjun, heihe city beef processing plant | 20150527 |
| SA550 | Raw meat | Mutton | unpacked | farmer’s market/street vendors | >500g | 2015-10-28 | Heilongjiang | Heihe city | West Road 61 Saibei meat shop | Heihe city, Wang Xiaoyan saibei meat processing plant | 20151028 |
| SA551 | Raw meat | Chicken | unpacked | farmer’s market/street vendors | >500g | 2015-05-27 | Heilongjiang | Heihe city | XING ' an Street 41-13 Double-Guan's Fu market, wholesale and retail fresh chicken stand | Heihe city, Guan's live chicken wholesale retail | 20150527 |
| SA552 | Raw meat | Chicken | unpacked | farmer’s market/street vendors | >500g | 2015-07-30 | Heilongjiang | Heihe city | Railway Street tiexi district farmers ' market the fatty chicken stand | Xiao sanjiazi Wang Xiao Wu, heihe city meat processing plant | 20150730 |
| SA553 | Raw meat | Duck | unpacked | farmer’s market/street vendors | >500g | 2015-10-28 | Heilongjiang | Heihe city | Huan Cheng Nan lu 168 Old age of HIV/AIDS, hotel | Old age of HIV/AIDS, heihe city hotel | 20151028 |
| SA554 | Raw meat | Beef | unpacked | farmer’s market/street vendors | >500g | 2015-05-27 | Heilongjiang | Hegang city | Bus road agricultural greenhouses 3 2 Bed | Unknown | 2015.5.27 |
| SA555 | Raw meat | Mutton | unpacked | farmer’s market/street vendors | >500g | 2015-08-05 | Heilongjiang | Hegang city | Hongqi Road South longitude, city and Xin lamb stand | Unknown | 2015.8.5 |
| SA556 | Raw meat | Duck | unpacked | farmer’s market/street vendors | >500g | 2015-05-28 | Heilongjiang | Hegang city | Junde road farmers ' market 5 Stand | Unknown | 2015.05.28 |
| SA557 | Raw meat | Beef | unpacked | farmer’s market/street vendors | >500g | 2015-10-20 | Heilongjiang | Harbin | Happy farm fresh fruits and vegetables https://item.taobao.com/item.htm?spm=a1z09.2.0.0.PaKd6j&id=39415142833&_u=mrhfevue4d9 | Happy farm fresh fruits and vegetables dot homemade | 20151020 |
| SA558 | Raw meat | Chicken | unpacked | farmer’s market/street vendors | >500g | 2015-10-20 | Heilongjiang | Harbin | Producers market 28 Stand | Unknown | 20151020 |
| SA559 | Raw meat | Beef | unpacked | farmer’s market/street vendors | >500g | 2015-06-02 | Heilongjiang | In jiamusi city | Blum, Anqing road farmers ' market | Unknown | 20150602 |
| SA560 | Raw meat | Pork | unpacked | Supermarket/department stores | >500g | 2015-10-19 | Heilongjiang | In jiamusi city | Devine Lu Zhong Duan Yonghui supermarket | Unknown | 20151019 |
| SA561 | Raw meat | Pork | unpacked | farmer’s market/street vendors | >500g | 2015-06-01 | Heilongjiang | In jixi city | Yongchang road 10 Guangyi, pork 71 Stand | Unknown | 20150601 |
| SA562 | Raw meat | Mutton | unpacked | farmer’s market/street vendors | >500g | 2015-11-05 | Heilongjiang | In jixi city | Yongchang road 10 Guangyi, lamb 1 Stand | Unknown | 20151105 |
| SA563 | Raw meat | Mutton | unpacked | farmer’s market/street vendors | >500g | 2015-11-05 | Heilongjiang | In jixi city | Yongchang road 10 Guangyi, lamb 3 Stand | Unknown | 20151105 |
| SA564 | Raw meat | Mutton | unpacked | Supermarket/department stores | >500g | 2015-11-05 | Heilongjiang | In jixi city | Liverpool Road 71 New supermarket | Unknown | 20151105 |
| SA565 | Raw meat | Duck | unpacked | Supermarket/department stores | >500g | 2015-06-01 | Heilongjiang | In jixi city | Nan gang new supermarket | Unknown | 20150601 |
| SA566 | Raw meat | Chicken | unpacked | Supermarket/department stores | >500g | 2015-06-01 | Heilongjiang | In jixi city | Nan gang new supermarket | Unknown | 20150601 |
| SA567 | Raw meat | Chicken | unpacked | farmer’s market/street vendors | >500g | 2015-06-01 | Heilongjiang | In jixi city | Yongchang road 10 Guang Yi city chicken 6 Stand | Unknown | 20150601 |
| SA568 | Raw meat | Chicken | unpacked | farmer’s market/street vendors | >500g | 2015-08-26 | Heilongjiang | In jixi city | Yongchang road 10 Guangyi split chicken 1 Stand | Unknown | 20150826 |
| SA569 | Raw meat | Duck | unpacked | farmer’s market/street vendors | >500g | 2015-08-26 | Heilongjiang | In jixi city | Yongchang road 10 Guang Yi city chicken 6 Stand | Unknown | 20150826 |
| SA570 | Raw meat | Duck | unpacked | farmer’s market/street vendors | >500g | 2015-08-26 | Heilongjiang | In jixi city | Yongchang road 10 Guang Yi cheng Song Lixin stand stand | Linqu Kang XING food co | 20150322 |
| SA571 | Raw meat | Chicken | unpacked | Supermarket/department stores | >500g | 2015-11-05 | Heilongjiang | In jixi city | Nan gang new supermarket | Unknown | 20151105 |
| SA572 | Raw meat | Chicken | unpacked | farmer’s market/street vendors | >500g | 2015-11-05 | Heilongjiang | In jixi city | Yongchang road 10 Guang Yi city chicken 1 Stand | Unknown | 20151105 |
| SA573 | Raw meat | Chicken | unpacked | farmer’s market/street vendors | >500g | 2015-11-05 | Heilongjiang | In jixi city | Yongchang road 10 Guang Yi city chicken 3 Stand | Unknown | 20151105 |
| SA574 | Raw meat | Pork | unpacked | farmer’s market/street vendors | >500g | 2015-06-01 | Heilongjiang | Mudanjiang city | BA Mian Jincheng group of raw meat in the market 2 Stand | Unknown | 20150601 |
| SA575 | Raw meat | Pork | unpacked | farmer’s market/street vendors | >500g | 2015-08-24 | Heilongjiang | Qiqihar | Long Sha Lu 258 Numbers station market 17 | Unknown | 20150824 |
| SA576 | Raw meat | Pork | unpacked | farmer’s market/street vendors | >500g | 2015-08-24 | Heilongjiang | Qiqihar | Yun Jie 111 Number five market third door left hand | Unknown | 20150824 |
| SA577 | Raw meat | Beef | unpacked | farmer’s market/street vendors | >500g | 2015-08-24 | Heilongjiang | Qiqihar | In the loop 1 Northeast market fourth door right hand | Unknown | 20150824 |
| SA578 | Raw meat | Mutton | unpacked | farmer’s market/street vendors | >500g | 2015-08-24 | Heilongjiang | Qiqihar | Chaoyang road 10 Liberation market tasty fresh lamb monopoly | Unknown | 20150824 |
| SA579 | Raw meat | Pork | unpacked | farmer’s market/street vendors | >500g | 2015-11-02 | Heilongjiang | Qiqihar | Jingxin Street 16 Number, jingxin market door first | Unknown | 20151102 |
| SA580 | Raw meat | Beef | unpacked | Supermarket/department stores | >500g | 2015-11-02 | Heilongjiang | Qiqihar | Academy Street 300 , RT-Mart supermarket | Unknown | 20151102 |
| SA581 | Raw meat | Chicken | unpacked | farmer’s market/street vendors | >500g | 2015-06-23 | Heilongjiang | Qiqihar | Stand ahead of Wu Yuan eggs poultry wholesale | Wan Jia, Xin, Shandong co | 2015-01-28 |
| SA582 | Raw meat | Duck | unpacked | farmer’s market/street vendors | >500g | 2015-06-23 | Heilongjiang | Qiqihar | Stand ahead of Wu Yuan eggs poultry wholesale | Shandong poly food limited | 2015-03-12 |
| SA583 | Raw meat | Chicken | unpacked | Supermarket/department stores | >500g | 2015-08-24 | Heilongjiang | Qiqihar | Longhua road 132 , RT-Mart supermarket | Unknown | 20150824 |
| SA584 | Raw meat | Chicken | unpacked | Supermarket/department stores | >500g | 2015-08-24 | Heilongjiang | Qiqihar | Halal way 18 Wal-Mart supermarket | Unknown | 20150824 |
| SA585 | Raw meat | Chicken | unpacked | farmer’s market/street vendors | >500g | 2015-08-24 | Heilongjiang | Qiqihar | Long Sha Lu 528 Standing before the upper-left corner of the first | Unknown | 20150824 |
| SA586 | Raw meat | Duck | unpacked | farmer’s market/street vendors | >500g | 2015-08-24 | Heilongjiang | Qiqihar | Cultural Street 247 And Liu Yuan market poultry monopoly | Unknown | 20150824 |
| SA587 | Raw meat | Duck | unpacked | farmer’s market/street vendors | >500g | 2015-08-24 | Heilongjiang | Qiqihar | Jingxin Street 16 Number, jingxin poultry market area the fourth row the second | Unknown | 20150824 |
| SA588 | Raw meat | Chicken | unpacked | Supermarket/department stores | >500g | 2015-11-02 | Heilongjiang | Qiqihar | Longhua road 132 , RT-Mart supermarket | Unknown | 20151102 |
| SA589 | Raw meat | Chicken | unpacked | farmer’s market/street vendors | >500g | 2015-11-02 | Heilongjiang | Qiqihar | Long Sha Lu 528 Numbers Station South Gate first poultry market | Unknown | 20151102 |
| SA590 | Raw meat | Chicken | unpacked | farmer’s market/street vendors | >500g | 2015-11-02 | Heilongjiang | Qiqihar | Chaoyang road 10 Liberation market poultry monopoly II | Unknown | 20151102 |
| SA591 | Raw meat | Beef | unpacked | farmer’s market/street vendors | >500g | 2015-07-27 | Heilongjiang | Qitaihe city | Choi Street South regional farmer's market fresh peach 8 Booth | Qitaihe city, Heilongjiang province, Xinyuan Taoshan District along the street meat company | 20150727 |
| SA592 | Raw meat | Mutton | unpacked | farmer’s market/street vendors | >500g | 2015-07-27 | Heilongjiang | Qitaihe city | Datong Road East area farmer's market fresh peach 6 Booth | Qitaihe city, Heilongjiang province, Xinyuan Taoshan District along the street meat company | 20150727 |
| SA593 | Raw meat | Chicken | unpacked | Supermarket/department stores | >500g | 2015-06-01 | Heilongjiang | Qitaihe city | Shan Hu Lu Darun supermarket | Unknown | 2015-5-29 |
| SA594 | Raw meat | Duck | unpacked | farmer’s market/street vendors | >500g | 2015-06-01 | Heilongjiang | Qitaihe city | Da Tong Lu Tao Dong farmer's market fresh meat 5 Booth | Unknown | 2015-6-1 |
| SA595 | Raw meat | Chicken | unpacked | Supermarket/department stores | >500g | 2015-11-05 | Heilongjiang | Qitaihe city | Kang Hua Jie Qing Ke long supermarket | Unknown | 20151105 |
| SA596 | Raw meat | Chicken | unpacked | Supermarket/department stores | >500g | 2015-11-05 | Heilongjiang | Qitaihe city | Kang Hua Jie Qing Ke long supermarket | Unknown | 20151105 |
| SA597 | Raw meat | Chicken | unpacked | farmer’s market/street vendors | >500g | 2015-11-05 | Heilongjiang | Qitaihe city | Friendship Street area and poultry trade city farmers ' markets 4 Booth | Unknown | 20151105 |
| SA598 | Raw meat | Beef | unpacked | farmer’s market/street vendors | >500g | 2015-06-01 | Heilongjiang | Mudanjiang city | Triad forest road 84 Dragon spring farmers market 16 Stand | Dongning County, Heilongjiang province, Mudanjiang city, individual farmers | 20150601 |
| SA599 | Raw meat | Beef | unpacked | farmer’s market/street vendors | >500g | 2015-09-21 | Heilongjiang | Mudanjiang city | Galiya road 76 MOM in Sichuan and Chongqing hotpot | Da du town, dongning County, Heilongjiang province dairy farms | 2015-09-21 |
| SA600 | Raw meat | Mutton | unpacked | farmer’s market/street vendors | >500g | 2015-09-21 | Heilongjiang | Mudanjiang city | Funingyaju 3 Lou 2 Retail fish, poor lamb hotpot | Suifenhe, Heilongjiang province, Chaoyang village of individual farmers | 2015-09-21 |
| SA601 | Raw meat | Pork | unpacked | farmer’s market/street vendors | >500g | 2015-10-26 | Heilongjiang | Mudanjiang city | The pedestrian street 47 Dennis, fresh meat | Mulin, Heilongjiang province, MA Qiao he Zhen individual farmers | 20151026 |
| SA602 | Raw meat | Chicken | unpacked | Supermarket/department stores | >500g | 2015-06-01 | Heilongjiang | Mudanjiang city | River Road 8 Ao Jiao long supermarket | Chinese God of Liaoning shuguang group of agriculture and animal husbandry nongan herding industry limited company | 20150601 |
| SA603 | Raw meat | Mutton | unpacked | farmer’s market/street vendors | >500g | 2015-05-26 | Heilongjiang | Suihua city | Sunshine farmer's Hall 73 Stand | Unknown | 20150526 |
| SA604 | Raw meat | Mutton | unpacked | Supermarket/department stores | >500g | 2015-08-05 | Heilongjiang | Suihua city | People of Xinglong Street supermarket | Unknown | 20150805 |
| SA605 | Raw meat | Duck | unpacked | Supermarket/department stores | >500g | 2015-05-26 | Heilongjiang | Suihua city | Zhengyang Street 844 Carrefour supermarket | Unknown | 20150526 |
| SA606 | Raw meat | Chicken | unpacked | farmer’s market/street vendors | >500g | 2015-05-26 | Heilongjiang | Suihua city | Nan Qi Jie Sun farmers slaughter hall Fortune Ltd | Unknown | 20150526 |
| SA607 | Raw meat | Chicken | unpacked | farmer’s market/street vendors | >500g | 2015-05-26 | Heilongjiang | Suihua city | Sunshine farmer's Hall 56 Stand | Unknown | 20150526 |
| SA608 | Raw meat | Chicken | unpacked | farmer’s market/street vendors | >500g | 2015-08-10 | Heilongjiang | Suihua city | 93 market 61 Stand | Unknown | 20150810 |
| SA609 | Raw meat | Pork | unpacked | farmer’s market/street vendors | >500g | 2015-06-16 | Heilongjiang | Shuangyashan city | New Street, guomao commercial building negative layer group assured farmers market raw meat cold meat shops | Unknown | 2015-06-16 |
| SA610 | Raw meat | Chicken | unpacked | farmer’s market/street vendors | >500g | 2015-06-16 | Heilongjiang | Shuangyashan city | New Street, guomao commercial building a farmer's market chicken group | Unknown | 2015-06-16 |
| SA611 | Raw meat | Chicken | unpacked | farmer’s market/street vendors | >500g | 2015-06-16 | Heilongjiang | Shuangyashan city | New Street, guomao commercial building a farmer's market chicken group Liu Jia | Unknown | 2015-06-16 |
| SA612 | Raw meat | Chicken | unpacked | Supermarket/department stores | >500g | 2015-06-02 | Heilongjiang | Yichun city | Heping road 37 Good luck to the supermarket, | Unknown | 20150602 |
| SA613 | Raw meat | Chicken | unpacked | Supermarket/department stores | >500g | 2015-06-08 | Henan | In Zhengzhou city | Great wall road and the West third ring road intersection 400 Meters South shuanghui chilled meat | Unknown | 2015-06-16 |
| SA614 | Raw meat | Chicken | unpacked | farmer’s market/street vendors | >500g | 2015-10-27 | Henan | In Zhengzhou city | Weisi Road market start of living chicken | Unknown | 2015-06-16 |
| SA615 | Raw meat | Chicken | unpacked | farmer’s market/street vendors | >500g | 2015-10-27 | Henan | In Zhengzhou city | Opened by the intersection of first street and South Road liming Transported live fish market day | Unknown | 2015-06-16 |
| SA616 | Raw meat | Chicken | unpacked | farmer’s market/street vendors | >500g | 2015-08-18 | Henan | Puyang city | Kun Wu Zhong Road, Puyang 372 Wave front set splitting the profits of the market segmentation, chicken | Unknown | 2015-06-16 |
| SA617 | Raw meat | Pork | unpacked | farmer’s market/street vendors | >500g | 2015-08-18 | Henan | Puyang city | Kun Wu Zhong Road, Puyang 372 Wave front set, Zhang xuemin meat shop in the market | Unknown | 2015-06-16 |
| SA618 | Raw meat | Mutton | unpacked | farmer’s market/street vendors | >500g | 2015-08-18 | Henan | Puyang city | Kun Wu Zhong Road, Puyang 372 Head Assembly market old Lee lamb | Unknown | 2015-06-16 |
| SA619 | Raw meat | Beef | unpacked | farmer’s market/street vendors | >500g | 2015-08-18 | Henan | Puyang city | Kun Wu Zhong Road, Puyang 372 Wave front set market, great beef shop | Unknown | 2015-06-16 |
| SA620 | Raw meat | Pork | unpacked | Supermarket/department stores | >500g | 2015-08-18 | Henan | Puyang city | Kun Wu Zhong Road, Puyang 406 Greentown Mall | Unknown | 2015-06-16 |
| SA621 | Raw meat | Pork | unpacked | Supermarket/department stores | >500g | 2015-08-17 | Henan | In pingdingshan city | Fuxing Lu Wan Shopping Centre | Unknown | 2015-8-17 |
| SA622 | Raw meat | Duck | unpacked | Supermarket/department stores | >500g | 2015-10-19 | Henan | Luoyang City | Kai yuan Avenue intersection with thick load Street yonghui supermarket | Ominous | 20151019 |
| SA623 | Raw meat | Chicken | unpacked | farmer’s market/street vendors | >500g | 2015-08-03 | Henan | Anyang City | Beiguan district Lu Li Kee stall | Unknown | 15-8-3 |
| SA624 | Raw meat | Pork | unpacked | Supermarket/department stores | >500g | 2015-04-01 | Hubei | Wuhan City | Hanyang Avenue, 470 Hundred warehouses in caidian Shopping Plaza, | Wuhan food co | 20150401 |
| SA625 | Raw meat | Pork | unpacked | Supermarket/department stores | >500g | 2015-04-01 | Hubei | Wuhan City | Garden Avenue 106 Hundred warehouses in wujiashan Shopping Plaza, | COFCO Wuhan meat co | 20150401 |
| SA626 | Raw meat | Beef | unpacked | Supermarket/department stores | >500g | 2015-04-20 | Hubei | In Yichang city | Xiling Yi lu 19-1 North Hills supermarket East Gate store | Ominous | 2015-06-16 |
| SA627 | Raw meat | Mutton | unpacked | Supermarket/department stores | >500g | 2015-04-20 | Hubei | In Yichang city | Stadium North Road, Yichang city, Hubei Province 169 Metro supermarket | Qiandao Lake sea flute ecological foods limited | 20141213 |
| SA628 | Raw meat | Beef | unpacked | farmer’s market/street vendors | >500g | 2015-04-20 | Hubei | In Yichang city | Xiling district of East Lake Road 8 East Lake farmers market | Unknown | 20150401 |
| SA629 | Raw meat | Pork | unpacked | Supermarket/department stores | >500g | 2015-04-20 | Hubei | In Yichang city | Nagasaka road 115 North Hills supermarket | Unknown | 20150420 |
| SA630 | Raw meat | Chicken | unpacked | Supermarket/department stores | >500g | 2015-04-13 | Hubei | Jingzhou city | Jiangjin road 204 RT-Mart supermarket | Jingzhou city living foods, Ltd | 20150413 |
| SA631 | Raw meat | Duck | unpacked | Supermarket/department stores | >500g | 2015-04-13 | Hubei | Jingzhou city | Hu Lu 17 Discuss the discount supermarket | Wuhan department store agricultural products company | 20150412 |
| SA632 | Raw meat | Chicken | unpacked | farmer’s market/street vendors | >500g | 2015-04-13 | Hubei | Jingzhou city | Poultry slaughtering point South Beijing road Green village farmers ' markets 99 | Southern District of Jingzhou, Hubei and Hunan agricultural trade logistics center D3513 | 20150413 |
| SA633 | Raw meat | Duck | unpacked | farmer’s market/street vendors | >500g | 2015-04-13 | Hubei | Jingzhou city | Aviation road, new market poultry slaughtering point South 1181 2 Cage | Jingzhou, Hubei and Hunan agricultural trade logistics center North D2345 | 20150413 |
| SA634 | Raw meat | Chicken | unpacked | farmer’s market/street vendors | >500g | 2015-04-13 | Hubei | Jingzhou city | Aviation road, new market poultry slaughtering point South 1174 5 Cage | Jingzhou, Hubei and Hunan agricultural trade logistics center North D2017 | 20150413 |
| SA635 | Raw meat | Duck | unpacked | farmer’s market/street vendors | >500g | 2015-04-13 | Hubei | Jingzhou city | Beijing East Road, Prairie farmers ' market poultry slaughtering D034 3 Cage | Jingzhou, Hubei and Hunan agricultural trade logistics center North D2345 | 20150413 |
| SA636 | Raw meat | Chicken | unpacked | farmer’s market/street vendors | >500g | 2015-04-13 | Hubei | Jingzhou city | Beijing East Road, Prairie farmers ' market poultry slaughtering D035 11 Cage | Jingzhou, Hubei and Hunan agricultural trade logistics center North D2017 | 20150413 |
| SA637 | Raw meat | Chicken | unpacked | farmer’s market/street vendors | >500g | 2015-04-13 | Hubei | Jingzhou city | Beijing East Road, Prairie farmers ' market poultry slaughtering D035 7 Cage | Jingzhou, Hubei and Hunan agricultural trade logistics center North D2017 | 20150413 |
| SA638 | Raw meat | Chicken | unpacked | farmer’s market/street vendors | >500g | 2015-04-13 | Hubei | Jingzhou city | Beijing East Road, Prairie farmers ' market poultry slaughtering D036 2 Cage | Jingzhou, Hubei and Hunan agricultural trade logistics center North D2017 | 20150413 |
| SA639 | Raw meat | Duck | unpacked | Supermarket/department stores | >500g | 2015-06-03 | Hubei | Shiyan city | New supermarket three Weirs store | Unknown | 20150413 |
| SA640 | Raw meat | Chicken | unpacked | farmer’s market/street vendors | >500g | 2015-06-03 | Hubei | Shiyan city | Dong Yue live poultry market X-18 | Unknown | 20150413 |
| SA641 | Raw meat | Chicken | unpacked | Supermarket/department stores | >500g | 2015-06-03 | Hubei | Shiyan city | Zhang Wan Dongfeng wonderful supermarket shop | Unknown | 20150413 |
| SA642 | Raw meat | Chicken | unpacked | farmer’s market/street vendors | >500g | 2015-06-13 | Hubei | Shiyan city | Zhuxi County Center farmers ' market | Unknown | 20150413 |
| SA643 | Raw meat | Duck | unpacked | farmer’s market/street vendors | >500g | 2015-06-13 | Hubei | Shiyan city | Vertical and horizontal farmers ' market | Unknown | 20150413 |
| SA644 | Raw meat | Duck | unpacked | Supermarket/department stores | >500g | 2015-04-19 | Hubei | Shiyan city | West Road, Wu discussed selling yunxi Shopping Plaza | Unknown | 20150413 |
| SA645 | Raw meat | Duck | unpacked | Supermarket/department stores | >500g | 2015-04-20 | Hubei | Shiyan city | Liuliping town new supermarket | Unknown | 20150413 |
| SA646 | Raw meat | Duck | unpacked | Supermarket/department stores | >500g | 2015-09-07 | Hubei | Shiyan city | Three Weirs new supermarket | Unknown | 20150413 |
| SA647 | Raw meat | Chicken | unpacked | Supermarket/department stores | >500g | 2015-09-07 | Hubei | Shiyan city | Three Weirs new supermarket | Unknown | 20150413 |
| SA648 | Raw meat | Chicken | unpacked | farmer’s market/street vendors | >500g | 2015-09-15 | Hubei | Shiyan city | Tiger ditches farmers ' market on the second floor 2 | Unknown | 20150915 |
| SA649 | Raw meat | Chicken | unpacked | Supermarket/department stores | >500g | 2015-09-17 | Hubei | Shiyan city | Shou Kang Yong Le Kang Dian | Unknown | 20150915 |
| SA650 | Raw meat | Chicken | unpacked | farmer’s market/street vendors | >500g | 2015-08-04 | Hubei | Shiyan city | JIU Li gang, Zhu Xi Center farmers ' market poultry meat sales | Unknown | 20150915 |
| SA651 | Raw meat | Chicken | unpacked | Supermarket/department stores | >500g | 2015-09-17 | Hubei | Shiyan city | Renmin road, into the supermarket | Unknown | 20150915 |
| SA652 | Raw meat | Chicken | unpacked | farmer’s market/street vendors | >500g | 2015-10-20 | Hubei | Shiyan city | Three Weirs farmers market live poultry 2 | Unknown | 20150915 |
| SA653 | Raw meat | Chicken | unpacked | Supermarket/department stores | >500g | 2015-10-20 | Hubei | Shiyan city | New supermarket six Weirs in the capital Stadium branch | Unknown | 20150915 |
| SA654 | Raw meat | Pork | unpacked | Supermarket/department stores | >500g | 2015-08-18 | Jiangsu | Changzhou City | Hua Cheng Zhong Lu 1-3 China resources ' suguo shopping mall, | Unknown | 20150915 |
| SA655 | Raw meat | Beef | unpacked | Supermarket/department stores | >500g | 2015-05-18 | Jiangsu | Salt City | Renmin road 12 Wen Feng Jia Hui, supermarket | Unknown | 20150915 |
| SA656 | Raw meat | Pork | unpacked | Supermarket/department stores | >500g | 2015-04-07 | Jilin | Tonghua city | Tuanjie road, European and Asian supermarkets in China are cold meat | Jilin huazheng agricultural development company | 20150915 |
| SA657 | Raw meat | Mutton | unpacked | farmer’s market/street vendors | >500g | 2015-04-08 | Jilin | Tonghua city | Farmers market road across the Hall and fresh beef and mutton pot | Tian Jia Tun slaughter | 2015.4.13 |
| SA658 | Raw meat | Pork | unpacked | farmer’s market/street vendors | >500g | 2015-04-21 | Jilin | Baishan city | Fusong Street 265 Liu Jidong stroking the market stall | O meat processing factory, Ltd | 2015.4.13 |
| SA659 | Raw meat | Pork | unpacked | farmer’s market/street vendors | >500g | 2015-04-29 | Jilin | Yanbian Korean autonomous prefecture | Chaoyang Zhen Xiang Yang Jie, Chaoyang town, Yanji City farmers market 219 Booth | Longjing city new pig slaughtering processing co | 2015.4.13 |
| SA660 | Raw meat | Pork | unpacked | Supermarket/department stores | >500g | 2015-06-18 | Jilin | Jilin city | Zunyi road 55 CRV | Changchun zhongpin food company | 2015.4.13 |
| SA661 | Raw meat | Beef | unpacked | Supermarket/department stores | >500g | 2015-06-18 | Jilin | Jilin city | Zunyi road 55 CRV | Changchun haoyue Muslim meat industry company limited | 2015.4.13 |
| SA662 | Raw meat | Chicken | unpacked | farmer’s market/street vendors | >500g | 2015-06-02 | Jilin | Siping city | Live chicken slaughtered fuping Benning street stand | Unknown | 2015.4.13 |
| SA663 | Raw meat | Duck | unpacked | Supermarket/department stores | >500g | 2015-07-06 | Jilin | In Changchun City | Eurasian Eurasian supermarket Plaza City 4 | Jilin Deda, Ltd | 2015.4.13 |
| SA664 | Raw meat | Duck | unpacked | farmer’s market/street vendors | >500g | 2015-07-06 | Jilin | In Changchun City | Small English chicken giblets Yatai integrated market stand Jilin road 1888 | Jilin Deda, Ltd | 2015.4.13 |
| SA665 | Raw meat | Chicken | unpacked | Supermarket/department stores | >500g | 2015-09-21 | Jiangxi | Fuzhou City | To purchase Le ' an Mall (supermarket) | Shanxi Lu Bao Jin Hesheng Foods Ltd | 2015.4.13 |
| SA666 | Raw meat | Duck | unpacked | farmer’s market/street vendors | >500g | 2015-11-20 | Jiangxi | Fuzhou City | II, yihuang town farmers market | No | 2015.4.13 |
| SA667 | Raw meat | Pork | unpacked | Supermarket/department stores | >500g | 2015-08-25 | Jiangxi | In Ganzhou city | Garden Center Pacific supermarket | Xingguo County, Pacific supermarket | 20150825 |
| SA668 | Raw meat | Mutton | unpacked | Supermarket/department stores | >500g | 2015-08-25 | Jiangxi | In Ganzhou city | Five Plaza, Sunshine square supermarket shop | Qingdao family food limited company | 20141107 |
| SA669 | Raw meat | Duck | unpacked | farmer’s market/street vendors | >500g | 2015-11-10 | Jiangxi | In Ganzhou city | Xu Kui Xiang lianjiangzhen Wu Feng CAI Shi Chang | Lianjiangzhen, Xingguo Xu Kui Xiang Wu Feng CAI Shi Chang | 20151110 |
| SA670 | Raw meat | Duck | unpacked | farmer’s market/street vendors | >500g | 2015-11-10 | Jiangxi | In Ganzhou city | Lianjiangzhen Ng Fung food market Zhang Jimei | Wu Feng CAI Shi Chang Zhang Jimei, lianjiangzhen, Xingguo | 20151110 |
| SA671 | Raw meat | Pork | unpacked | Supermarket/department stores | >500g | 2015-08-25 | Jiangxi | In Ganzhou city | Five Plaza, Sunshine square supermarket shop | Xingguo County sunshine square supermarket shop | 20150825 |
| SA672 | Raw meat | Pork | unpacked | farmer’s market/street vendors | >500g | 2015-10-20 | Jiangxi | JI ' an city | Granite Street, market fresh 25 Booth | Unknown | 2015-10-20 |
| SA673 | Raw meat | Duck | unpacked | farmer’s market/street vendors | >500g | 2015-10-27 | Jiangxi | JI ' an city | Shaoshan road farmers market poultry meat 12 Booth | Unknown | 2015-10-27 |
| SA674 | Raw meat | Chicken | unpacked | farmer’s market/street vendors | >500g | 2015-10-27 | Jiangxi | JI ' an city | cs | Unknown | 2015-10-27 |
| SA675 | Raw meat | Duck | unpacked | farmer’s market/street vendors | >500g | 2015-10-27 | Jiangxi | JI ' an city | Book Street East of market poultry stalls | Unknown | 2015-10-27 |
| SA676 | Raw meat | Duck | unpacked | farmer’s market/street vendors | >500g | 2015-10-27 | Jiangxi | JI ' an city | Four seasons spring market 6 Booth | Unknown | 2015-10-27 |
| SA677 | Raw meat | Chicken | unpacked | farmer’s market/street vendors | >500g | 2015-10-27 | Jiangxi | JI ' an city | Stone street market area and poultry 7 Booth | Unknown | 2015-10-27 |
| SA678 | Raw meat | Chicken | unpacked | farmer’s market/street vendors | >500g | 2015-08-24 | Jiangxi | Jingdezhen city | Home market (third live poultry stalls) | Home market (third live poultry stalls) | 20150824 |
| SA679 | Raw meat | Chicken | unpacked | farmer’s market/street vendors | >500g | 2015-08-24 | Jiangxi | Jingdezhen city | Yu Lu, farmer's market | Yu Lu, farmer's market | 20150824 |
| SA680 | Raw meat | Chicken | unpacked | farmer’s market/street vendors | >500g | 2015-08-24 | Jiangxi | Jingdezhen city | West Village Road market | New West market | 20150824 |
| SA681 | Raw meat | Pork | unpacked | farmer’s market/street vendors | >500g | 2015-08-24 | Jiangxi | Jingdezhen city | New factory farms | New factory farms | 20150824 |
| SA682 | Raw meat | Beef | unpacked | farmer’s market/street vendors | >500g | 2015-07-20 | Jiangxi | Jingdezhen city | West Road vegetable market in Vang Vieng booth | Unknown | 20150720 |
| SA683 | Raw meat | Pork | unpacked | farmer’s market/street vendors | >500g | 2015-07-20 | Jiangxi | Jingdezhen city | Taibai Park farmers ' market Zhang Maozi booth | Unknown | 20150720 |
| SA684 | Raw meat | Pork | unpacked | farmer’s market/street vendors | >500g | 2015-09-14 | Jiangxi | Jingdezhen city | XING he Wan the farmers ' market | Unknown | 20150914 |
| SA685 | Raw meat | Pork | unpacked | farmer’s market/street vendors | >500g | 2015-08-24 | Jiangxi | Jingdezhen city | School of electrical engineering in Jingdezhen city, turning meat | School of electrical engineering in Jingdezhen city, turning meat | 20150824 |
| SA686 | Raw meat | Duck | unpacked | farmer’s market/street vendors | >500g | 2015-07-20 | Jiangxi | Jingdezhen city | North China Road farmers ' market | Unknown | 20150720 |
| SA687 | Raw meat | Duck | unpacked | Supermarket/department stores | >500g | 2015-08-24 | Jiangxi | Jingdezhen city | New day new supermarket | New day new supermarket | 20150824 |
| SA688 | Raw meat | Pork | unpacked | farmer’s market/street vendors | >500g | 2015-08-24 | Jiangxi | Jingdezhen city | Chateau farmers market | Chateau farmers market | 20150824 |
| SA689 | Raw meat | Pork | unpacked | farmer’s market/street vendors | >500g | 2015-07-20 | Jiangxi | Jingdezhen city | Electrical and mechanical engineering school in Jingdezhen city, junction market Wang Lei stand | Unknown | 20150720 |
| SA690 | Raw meat | Duck | unpacked | farmer’s market/street vendors | >500g | 2015-07-20 | Jiangxi | Jingdezhen city | Electrical and mechanical engineering school in Jingdezhen city, junction market | Unknown | 20150720 |
| SA691 | Raw meat | Chicken | unpacked | farmer’s market/street vendors | >500g | 2015-07-20 | Jiangxi | Jingdezhen city | Farmers ' market Spray booths | Unknown | 20150720 |
| SA692 | Raw meat | Pork | unpacked | farmer’s market/street vendors | >500g | 2015-08-24 | Jiangxi | Jingdezhen city | New factory farms | New factory farms | 20150824 |
| SA693 | Raw meat | Pork | unpacked | Supermarket/department stores | >500g | 2015-10-08 | Jiangxi | Jiujiang city | De ' an, Chaoyang lushanbinlong supermarket | De ' an food company | 20151008 |
| SA694 | Raw meat | Pork | unpacked | farmer’s market/street vendors | >500g | 2015-07-21 | Jiangxi | Jiujiang city | De ' an Ho Dong Township township near Shi Hansong | Unknown | 20150721 |
| SA695 | Raw meat | Pork | unpacked | farmer’s market/street vendors | >500g | 2015-07-21 | Jiangxi | Jiujiang city | De ' an, Chaoyang Road market paint booth | Unknown | 20150721 |
| SA696 | Raw meat | Pork | unpacked | Supermarket/department stores | >500g | 2015-07-21 | Jiangxi | Jiujiang city | De ' an era of Dongfeng road, next to the old square Emporium supermarket | Unknown | 20150721 |
| SA697 | Raw meat | Pork | unpacked | farmer’s market/street vendors | >500g | 2015-07-21 | Jiangxi | Jiujiang city | De ' an Ho Dong Township Township next to Xiong Zu | Unknown | 20150721 |
| SA698 | Raw meat | Mutton | unpacked | farmer’s market/street vendors | >500g | 2015-10-08 | Jiangxi | Jiujiang city | De ' an, Chaoyang Lu CAI Shi Chang Li SI Horse stalls | Unknown | 20151008 |
| SA699 | Raw meat | Chicken | unpacked | farmer’s market/street vendors | >500g | 2015-04-20 | Jiangxi | Jiujiang city | Komsomolsk-na-Amure road Xin Gong Aiying within market stalls | Unknown | 20150721 |
| SA700 | Raw meat | Chicken | unpacked | farmer’s market/street vendors | >500g | 2015-04-20 | Jiangxi | Jiujiang city | Komsomolsk-na-Amure road Cha Bin in the village market stalls | Unknown | 20150721 |
| SA701 | Raw meat | Pork | unpacked | farmer’s market/street vendors | >500g | 2015-09-18 | Jiangxi | Jiujiang city | Dragon city vegetable market Yuan Jinmin individual meat | Yellow food station | 20150918 |
| SA702 | Raw meat | Mutton | unpacked | farmer’s market/street vendors | >500g | 2015-10-17 | Jiangxi | Jiujiang city | Along the road 277 (Farmers ' market 9 Shop) | Unknown | 20151015 |
| SA703 | Raw meat | Pork | unpacked | farmer’s market/street vendors | >500g | 2015-11-23 | Jiangxi | In Nanchang city | Xinjian Hong Ling Ling Road farmers market gold standard stall | No | 20151123 |
| SA704 | Raw meat | Duck | unpacked | Supermarket/department stores | >500g | 2015-11-23 | Jiangxi | In Nanchang city | Feng and South Avenue 2988 CRV Mediterranean Sun shop | No | 20151123 |
| SA705 | Raw meat | Chicken | unpacked | Supermarket/department stores | >500g | 2015-11-23 | Jiangxi | In Nanchang city | Feng and South Avenue 2988 CRV Mediterranean Sun shop | No | 20151123 |
| SA706 | Raw meat | Pork | unpacked | Supermarket/department stores | >500g | 2015-11-23 | Jiangxi | In Nanchang city | Feng and South Avenue 2988 CRV Mediterranean Sun shop | No | 20151123 |
| SA707 | Raw meat | Pork | unpacked | farmer’s market/street vendors | >500g | 2015-11-23 | Jiangxi | In Nanchang city | Hong Gu Tan Yu Lu Hong Gu Yu fang, eight farms | No | 20151123 |
| SA708 | Raw meat | Chicken | unpacked | farmer’s market/street vendors | >500g | 2015-08-03 | Jiangxi | Pingxiang city | Small Liu village chicken duck shop | Unknown | 20151123 |
| SA709 | Raw meat | Duck | unpacked | farmer’s market/street vendors | >500g | 2015-08-03 | Jiangxi | Pingxiang city | Small Liu village chicken duck shop | Unknown | 20151123 |
| SA710 | Raw meat | Chicken | unpacked | farmer’s market/street vendors | >500g | 2015-08-03 | Jiangxi | Pingxiang city | Huangtuling authentic chicken duck poultry shops | Unknown | 20151123 |
| SA711 | Raw meat | Pork | unpacked | farmer’s market/street vendors | >500g | 2015-09-15 | Jiangxi | Shangrao city | Duke Wen, Wuyuan Mall farmers ' market stalls | Hubei star farm | 20150915 |
| SA712 | Raw meat | Duck | unpacked | Supermarket/department stores | >500g | 2015-10-27 | Jiangxi | Shangrao city | New supermarket in TriStar hotel | Shandong zoucheng | 20151027 |
| SA713 | Raw meat | Pork | unpacked | Supermarket/department stores | >500g | 2015-09-15 | Jiangxi | Shangrao city | Guang Yuan, Wuyuan tea road | Wuyuan local | 20150915 |
| SA714 | Raw meat | Duck | unpacked | Supermarket/department stores | >500g | 2015-09-15 | Jiangxi | Shangrao city | Paramount supermarket era, Wuyuan Dian Wen Gong bei Lu | Unknown | 20150915 |
| SA715 | Raw meat | Pork | unpacked | Supermarket/department stores | >500g | 2015-08-10 | Jiangxi | Xinyu city | Wal-Mart (Jiangxi) commercial retail limited stores | Green Park, Xinyu city meat co | 20150810 |
| SA716 | Raw meat | Duck | unpacked | Supermarket/department stores | >500g | 2015-08-10 | Jiangxi | Xinyu city | Wal-Mart (Jiangxi) commercial retail limited Xinyu bouldering Park branch | Merchants cold beauty (Wuhan) | 20150805 |
| SA717 | Raw meat | Duck | unpacked | farmer’s market/street vendors | >500g | 2015-08-10 | Jiangxi | Xinyu city | Yushui district, forest guards smiling market poultry stalls | Yushui district of Xinyu city farmers ' market poultry wholesale | 20150810 |
| SA718 | Raw meat | Beef | unpacked | farmer’s market/street vendors | >500g | 2015-10-12 | Jiangxi | Yichun city | New Zou Jia camphor trees farmers market right at the door of the first House | No | 20150810 |
| SA719 | Raw meat | Pork | unpacked | Supermarket/department stores | >500g | 2015-10-12 | Jiangxi | Yichun city | Fengcheng city Wal-Mart supermarket | No | 20150810 |
| SA720 | Raw meat | Chicken | unpacked | farmer’s market/street vendors | >500g | 2015-10-12 | Jiangxi | Yichun city | Fengcheng City Centre market T2-28 | No | 20150810 |
| SA721 | Raw meat | Mutton | unpacked | Supermarket/department stores | >500g | 2015-10-12 | Jiangxi | Yichun city | Fengcheng city Wal-Mart supermarket | Shanghai herding Khan foods limited | 2015.9.26 |
| SA722 | Raw meat | Duck | unpacked | Supermarket/department stores | >500g | 2015-10-12 | Jiangxi | Yichun city | Fengcheng city Yi Wanjia supermarket | No | 2015.9.26 |
| SA723 | Raw meat | Duck | unpacked | farmer’s market/street vendors | >500g | 2015-10-12 | Jiangxi | Yichun city | East Gate market of cinnamomum camphora C21 Booth | No | 2015.9.26 |
| SA724 | Raw meat | Beef | unpacked | farmer’s market/street vendors | >500g | 2015-10-12 | Jiangxi | Yichun city | East Gate market of cinnamomum camphora lanlong booth | No | 2015.9.26 |
| SA725 | Raw meat | Chicken | unpacked | farmer’s market/street vendors | >500g | 2015-10-12 | Jiangxi | Yichun city | Cinnamomum camphora Zou Jia, farmers ' markets and new birds first | No | 2015.9.26 |
| SA726 | Raw meat | Duck | unpacked | Supermarket/department stores | >500g | 2015-10-11 | Jiangxi | Yichun city | Fengcheng town Yue Ke long supermarket | No | 2015.9.26 |
| SA727 | Raw meat | Duck | unpacked | farmer’s market/street vendors | >500g | 2015-07-06 | Jiangxi | Yichun city | Luo town, fengxin County Nong Mao Shi Chang's chicken and duck | Luo town, fengxin County Nong Mao Shi Chang's chicken and duck | 20150706 |
| SA728 | Raw meat | Duck | unpacked | farmer’s market/street vendors | >500g | 2015-06-29 | Jiangxi | Yichun city | Fengchuanzhen East Gate market poultry stalls | Within the in fengxin County fengchuanzhen East Gate market poultry stalls | 20150629 |
| SA729 | Raw meat | Chicken | unpacked | farmer’s market/street vendors | >500g | 2015-11-09 | Jiangxi | Yichun city | Fengxin Simon market fengchuanzhen chickens, ducks and birds | Fengxin Simon market fengchuanzhen chickens, ducks and birds | 2015.11.09 |
| SA730 | Raw meat | Pork | unpacked | farmer’s market/street vendors | >500g | 2015-07-06 | Jiangxi | Yichun city | Fengxin County town farmers market's meat | Fengxin County town farmers market's meat | 20150706 |
| SA731 | Raw meat | Duck | unpacked | farmer’s market/street vendors | >500g | 2015-08-10 | Zhejiang | Huzhou city | Rose Hill town, wuxing district, huzhou city Sanhe home farmers ' market C3 | Unknown | 20150706 |
| SA732 | Raw meat | Pork | unpacked | farmer’s market/street vendors | >500g | 2015-10-26 | Zhejiang | In Lishui city | Tian Shi long farms 1 Booth | Unknown | 20150706 |
| SA733 | Raw meat | Pork | unpacked | farmer’s market/street vendors | >500g | 2015-10-26 | Zhejiang | In Lishui city | Farmers ' market 21 | Unknown | 20150706 |
| SA734 | Raw meat | Chicken | unpacked | farmer’s market/street vendors | >500g | 2015-10-21 | Zhejiang | Shaoxing city | To make the market ( Lake Road 258 ) 63 | Unknown | 20150706 |
| SA735 | Raw meat | Duck | unpacked | farmer’s market/street vendors | >500g | 2015-10-21 | Zhejiang | Shaoxing city | To make the market ( Lake Road 258 ) 63 | Unknown | 20150706 |
| SA736 | Raw meat | Pork | unpacked | farmer’s market/street vendors | >500g | 2015-10-26 | Zhejiang | Taizhou city | West Street, jiaojiang, Taizhou 289-3 Jia interlingual transfer market 194 Booth | Unknown | 20150706 |
| SA737 | Raw meat | Pork | unpacked | farmer’s market/street vendors | >500g | 2015-04-14 | Zhejiang | Wenzhou City | Marina Park farmers market Luo Weili Huimin pork stalls | Unknown | 20150706 |
| SA738 | Raw meat | Beef | unpacked | farmer’s market/street vendors | >500g | 2015-10-13 | Zhejiang | Zhoushan City | Changguo Lu, Dinghai district, Zhoushan City 308 Chang Dong market 48 Booth | Changguo Lu, Dinghai district, Zhoushan City 308 Chang Dong market 48 Booth | 2015-10-13 |
| SA739 | Raw meat | Pork | unpacked | farmer’s market/street vendors | >500g | 2015-10-13 | Zhejiang | Zhoushan City | Penglai road, Dinghai district, Zhoushan City 103 East Gate market 15 Booth | Penglai road, Dinghai district, Zhoushan City 103 East Gate market 15 Booth | 2015-11-9 |
| SA740 | Raw meat | Duck | unpacked | Supermarket/department stores | >500g | 2015-07-13 | Xinjiang | Urumqi city | Sai Wai Huan bei Lu 989 West outer ring, friendly supermarket shop | Ominous | 20150713 |
| SA741 | Raw meat | Chicken | unpacked | Supermarket/department stores | >500g | 2015-07-13 | Xinjiang | Urumqi city | Sai Wai Huan bei Lu 989 West outer ring, friendly supermarket shop | Ominous | 20150713 |
| SA742 | Raw meat | Chicken | unpacked | Supermarket/department stores | >500g | 2015-07-13 | Xinjiang | Urumqi city | Xinshi district, Hebei CRV, East Hebei road | Ominous | 20150713 |
| SA743 | Raw meat | Chicken | unpacked | Supermarket/department stores | >500g | 2015-07-13 | Xinjiang | Urumqi city | Xinshi district, Hebei CRV, East Hebei road | Ominous | 20150713 |
| SA744 | Raw meat | Chicken | unpacked | farmer’s market/street vendors | >500g | 2015-10-13 | Xinjiang | Urumqi city | Nine meter restaurant South of Central Asia 330 | / | 2015-10-13 |
| SA745 | Raw meat | Mutton | unpacked | Supermarket/department stores | >500g | 2015-06-01 | Xinjiang | Mongolia autonomons State | Hejing County Lu Man Fu supermarkets | Unknown | 20150601 |
| SA746 | Raw meat | Chicken | unpacked | farmer’s market/street vendors | >500g | 2015-06-01 | Xinjiang | Mongolia autonomons State | Jesus hejing County zedongluxing open market of halal slaughter a chicken shop | Unknown | 20150601 |
| SA747 | Raw meat | Chicken | unpacked | Supermarket/department stores | >500g | 2015-11-10 | Xinjiang | Karamay city | Karamay Hui Jia HUI Ka times department store supermarket | Haicheng city, Liaoning province, Geng Chong Zhen | 20150910 |
| SA748 | Raw meat | Beef | unpacked | farmer’s market/street vendors | >500g | 2015-07-09 | Xinjiang | Karamay city | North Park Spring seafood restaurant | Unknown | 20150709 |
| SA749 | Raw meat | Chicken | unpacked | farmer’s market/street vendors | >500g | 2015-07-14 | Xinjiang | Karamay city | Urho district, the golden triangle of market 2 Booth | Urho district, 137 Group nine | 20150714 |
| SA750 | Raw meat | Chicken | unpacked | farmer’s market/street vendors | >500g | 2015-07-14 | Xinjiang | Karamay city | Urho district, Wu Yang Bo shop | Urho district, green meat co | 20150713 |
| SA751 | Raw meat | Mutton | unpacked | farmer’s market/street vendors | >500g | 2015-07-14 | Xinjiang | Karamay city | Urho district, Wu Yang Bo shop | Urho district, the golden triangle of market | 20150713 |
| SA752 | Raw meat | Chicken | unpacked | farmer’s market/street vendors | >500g | 2015-11-11 | Xinjiang | Karamay city | Abundant green salt in Karamay limited vegetable wholesale vegetable market temporary stalls | Karamay poultry slaughterhouses | 20151111 |
| SA753 | Raw meat | Pork | unpacked | Supermarket/department stores | >500g | 2015-08-18 | Beijing | Dongcheng District | Beijing new world supermarket new world department store (Chong Wai da Jie 3 Number) | China Foods Limited (North exit of Fengtai West race course 1 Number) | 20150818 |
| SA754 | Raw meat | Chicken | unpacked | Supermarket/department stores | >500g | 2015-08-18 | Beijing | Dongcheng District | Beijing new world supermarket new world department store (Chong Wai da Jie 3 Number) | Tyson Donghua Foods Ltd (chongchuan district, Nantong, Jiangsu Province, chongchuan road 1 Number) | 20150818 |
| SA755 | Raw meat | Pork | unpacked | Supermarket/department stores | >500g | 2015-09-16 | Beijing | Dongcheng District | Beijing new world supermarket new world department store (Chong Wai da Jie 3 Number) | Ominous | 20150916 |
| SA756 | Raw meat | Mutton | unpacked | Supermarket/department stores | >500g | 2015-09-16 | Beijing | Dongcheng District | Beijing new world supermarket new world department store (Chong Wai da Jie 3 Number) | Ominous | 20150916 |
| SA757 | Raw meat | Chicken | unpacked | Supermarket/department stores | >500g | 2015-07-21 | Beijing | Fengtai District | Huatang shopping mall Qi Li Zhuang, store | Unknown | 20150720 |
| SA758 | Raw meat | Chicken | unpacked | farmer’s market/street vendors | >500g | 2015-05-18 | Beijing | Haidian District | Qing he Road West second Street North 12 West two flags of convenience, fresh meat market area 1 2 | Unknown | 20150518 |
| SA759 | Raw meat | Beef | unpacked | Supermarket/department stores | >500g | 2015-06-11 | Beijing | Haidian District | Wal-Mart Supercenter ZHICHUN road | Constant of Chongqing agricultural development co | 20150611 |
| SA760 | Raw meat | Duck | unpacked | Supermarket/department stores | >500g | 2015-08-17 | Beijing | Haidian District | Xisanqi jingkelong supermarket shop | Xisanqi jingkelong supermarket shop | 20150817 |
| SA761 | Raw meat | Chicken | unpacked | Supermarket/department stores | >500g | 2015-04-20 | Beijing | Miyun district | Beijing Miyun city supermarket (cloud to new road 21 Number) | Beijing Dafa Zhengda co | 20150420 |
| SA762 | Raw meat | Beef | unpacked | Supermarket/department stores | >500g | 2015-04-20 | Beijing | Miyun district | Beijing Miyun city supermarket (cloud to new road 21 Number) | Xianghe County, Hebei province rongxin meat co | 20150420 |
| SA763 | Raw meat | Pork | unpacked | farmer’s market/street vendors | >500g | 2015-04-20 | Beijing | Miyun district | New road, Miyun 68 Miyun County, Beijing huayuan fresh meat market hall 61 Booth | Beijing pengcheng foods limited | 20150420 |
| SA764 | Raw meat | Pork | unpacked | Supermarket/department stores | >500g | 2015-07-27 | Beijing | Xicheng District | Wumart, Beijing xinjiekou store | Beijing shunxin agriculture food branch | 20150727 |
| SA765 | Raw meat | Pork | unpacked | Supermarket/department stores | >500g | 2015-07-27 | Beijing | Xicheng District | Wumart, Beijing xinjiekou store | Beijing shunxin agriculture food branch | 20150727 |
| SA766 | Raw meat | Chicken | unpacked | Supermarket/department stores | >500g | 2015-07-27 | Beijing | Xicheng District | Wumart, Beijing xinjiekou store | Beijing huadu broiler Corporation | 20150727 |
| SA767 | Raw meat | Chicken | unpacked | Supermarket/department stores | >500g | 2015-10-19 | Beijing | Xicheng District | Beijing deshengmen merrymart supermarket shop | Shandong great foodstuff processing co | 20150901 |
| SA768 | Raw meat | Chicken | unpacked | Supermarket/department stores | >500g | 2015-10-19 | Beijing | Xicheng District | Beijing deshengmen merrymart supermarket shop | Shandong great foodstuff processing co | 20150710 |
| SA769 | Raw meat | Chicken | unpacked | Supermarket/department stores | >500g | 2015-10-19 | Beijing | Xicheng District | Beijing deshengmen merrymart supermarket shop | Beijing huadu broiler Corporation | 20151019 |
| SA770 | Raw meat | Chicken | unpacked | Supermarket/department stores | >500g | 2015-10-19 | Beijing | Xicheng District | Beijing deshengmen merrymart supermarket shop | Beijing huadu broiler Corporation | 20151019 |
| SA771 | Raw meat | Pork | unpacked | Supermarket/department stores | >500g | 2015-10-19 | Beijing | Xicheng District | Beijing deshengmen merrymart supermarket shop | Beijing v meatworks | 20151019 |
| SA772 | Raw meat | Pork | unpacked | Supermarket/department stores | >500g | 2015-10-19 | Beijing | Xicheng District | Beijing deshengmen merrymart supermarket shop | Beijing v meatworks | 20151019 |
| SA773 | Raw meat | Beef | unpacked | Supermarket/department stores | >500g | 2015-10-19 | Beijing | Xicheng District | Beijing deshengmen merrymart supermarket shop | Changchun, Jilin province, haoyue Muslim meat industry company limited | 20151017 |
| SA774 | Raw meat | Chicken | unpacked | farmer’s market/street vendors | >500g | 2015-05-06 | Sichuan | In Nanchong city | Que Wang Xiaoli town farmers ' markets stalls | No | 20150713 |
| SA775 | Raw meat | Duck | unpacked | farmer’s market/street vendors | >500g | 2015-05-07 | Sichuan | Guang ' an city | Xia Jie gmelinii 62 Guizhou black chicken with bamboo shoots | Guizhou black bamboo chicken | 20150507 |
| SA776 | Raw meat | Duck | unpacked | farmer’s market/street vendors | >500g | 2015-08-06 | Sichuan | In Nanchong city | Liu Ziyun Township farmers ' markets store | No | 20150713 |
| SA777 | Raw meat | Chicken | unpacked | farmer’s market/street vendors | >500g | 2015-09-14 | Sichuan | Ziyang city | Red West market poultry, seafood, frozen food business Department | Production | 20150914 |
| SA778 | Raw meat | Pork | unpacked | farmer’s market/street vendors | >500g | 2015-09-07 | Sichuan | Panzhihua city | Nong Nong ping panzhihua city, Middle East market | / | 20150914 |
| SA779 | Raw meat | Pork | unpacked | farmer’s market/street vendors | >500g | 2015-09-07 | Sichuan | Panzhihua city | MIDI bridge farmers market 1 Booth | / | 20150914 |
| SA780 | Raw meat | Pork | unpacked | farmer’s market/street vendors | >500g | 2015-10-27 | Sichuan | Panzhihua city | Benevolence and old farmers ' market 2-080 | / | 20150914 |
| SA781 | Raw meat | Chicken | unpacked | farmer’s market/street vendors | >500g | 2015-11-10 | Sichuan | In Nanchong city | Little street market chicken wholesale Department | No | 20150914 |
| SA782 | Raw meat | Mutton | unpacked | Supermarket/department stores | >500g | 2015-05-18 | Jiangsu | Xuzhou city | Peace Avenue 58 Lotte supermarket, Wanda Plaza, | Unknown | 20150914 |
| SA783 | Raw meat | Pork | unpacked | farmer’s market/street vendors | >500g | 2015-05-18 | Jiangsu | Xuzhou city | Min Xiang Xiang Yuan Road Park farmers ' market 11 Booth | Unknown | 20150914 |
| SA784 | Raw meat | Pork | unpacked | Supermarket/department stores | >500g | 2015-08-05 | Jiangsu | Xuzhou city | The Beltway 129 Suguo supermarket | Unknown | 20150914 |
| SA785 | Raw meat | Beef | unpacked | farmer’s market/street vendors | >500g | 2015-05-12 | Jiangsu | Changzhou City | Ring Rd 56 Ninth row Fung Lok market, 10 Booth | Unknown | 20150914 |
| SA786 | Raw meat | Pork | unpacked | Supermarket/department stores | >500g | 2015-05-12 | Jiangsu | Changzhou City | Lu Cheng Street Park 1 Garden 12 Chuang Yi-Hua supermarkets | Yi-Hua supermarkets | 20150914 |
| SA787 | Raw meat | Pork | unpacked | farmer’s market/street vendors | >500g | 2015-08-17 | Jiangsu | Changzhou City | Yan lu 6 Yanshan, agricultural and sideline products, Ltd | Unknown | 20150914 |
| SA788 | Raw meat | Duck | unpacked | farmer’s market/street vendors | >500g | 2015-07-13 | Jiangsu | In Huaian city | Qingjiang Street road 2 , Lotus pond farmers ' market fourth towards the East of the South Gate | Lily pond farmers market to the East of the South Gate's fourth | 20150914 |
| SA789 | Raw meat | Duck | unpacked | Supermarket/department stores | >500g | 2015-09-06 | Jiangsu | In Huaian city | South Gate Street 2 Lotte supermarket freezers for meat products, | South Gate Street 2 Lotte supermarket freezers for meat products, | 20150906 |
| SA790 | Raw meat | Pork | unpacked | Supermarket/department stores | >500g | 2015-05-18 | Jiangsu | Salt City | Jianjun Zhong Lu 59 Lotte | Unknown | 20150517 |
| SA791 | Raw meat | Beef | unpacked | Supermarket/department stores | >500g | 2015-06-28 | Jiangsu | Salt City | Huiwen road, 2 Carrefour supermarket | Unknown | 20150628 |
| SA792 | Raw meat | Chicken | unpacked | farmer’s market/street vendors | >500g | 2015-05-26 | Gansu | In dingxi city | Traffic road Dong Guan Yang incoming live chickens in the market store | Settled in dingxi region, Gansu province, Lu Dong Guan Shi Yang incoming live chicken shops | 20150526 |
| SA793 | Raw meat | Chicken | unpacked | farmer’s market/street vendors | >500g | 2015-08-24 | Gansu | Gannan Tibetan Autonomous Prefecture | West all the way to the market near the mahasangan draw fire chicken | Linxia (unknown) | 20150824 |
| SA794 | Raw meat | Chicken | unpacked | farmer’s market/street vendors | >500g | 2015-08-24 | Gansu | Gannan Tibetan Autonomous Prefecture | West all the way to the market near the mahasangan draw fire chicken | Linxia (unknown) | 20150824 |
| SA795 | Raw meat | Chicken | unpacked | farmer’s market/street vendors | >500g | 2015-08-17 | Gansu | In Jiuquan city | Suzhou meat market area | Unknown | 20150824 |
| SA796 | Raw meat | Chicken | unpacked | Supermarket/department stores | >500g | 2015-10-12 | Gansu | In Jiuquan city | Drum tower north Oriental Plaza, China resources Vanguard supermarket | Unknown | 20150824 |
| SA797 | Raw meat | Pork | unpacked | farmer’s market/street vendors | >500g | 2015-04-27 | Gansu | In Lanzhou city | East Jiefang road supermarket | Unknown | 20150824 |
| SA798 | Raw meat | Duck | unpacked | Supermarket/department stores | >500g | 2015-09-06 | Gansu | In Pingliang city | Xin min road, new century supermarket new store | Shaanxi Shi Yang foods limited | 20150824 |
| SA799 | Raw meat | Duck | unpacked | farmer’s market/street vendors | >500g | 2015-05-13 | Gansu | In qingyang city | Ring road farmers market Liu aquatic shop | Unknown | 20150824 |
| SA800 | Raw meat | Pork | unpacked | farmer’s market/street vendors | >500g | 2015-08-17 | Gansu | Zhangye city | Nan Guan Zhang Zhenmin butcher shop | No | 20150824 |
| SA801 | Raw meat | Chicken | unpacked | farmer’s market/street vendors | >500g | 2015-08-17 | Gansu | Zhangye city | Chicken tax Ting yan Jie fu-Peng market shop | No | 20150824 |
| SA802 | Raw meat | Chicken | unpacked | farmer’s market/street vendors | >500g | 2015-08-17 | Gansu | Zhangye city | New music community near the community hot chicken restaurant | No | 2015-08-17 |
| SA803 | Raw meat | Mutton | unpacked | farmer’s market/street vendors | >500g | 2015-11-03 | Gansu | Zhangye city | Market he Jian Jun, North Road shops | Unknown | 20151103 |
| SA804 | Rice- and flour-products | Rice- and flour-products | unpacked | farmer’s market/street vendors | >500g | 2015-05-05 | Anhui | , Fuyang city | Jade Jie North West happy breakfast shop | Jade Jie North West happy breakfast shop | 20150505 |
| SA805 | Rice- and flour-products | Rice- and flour-products | unpacked | farmer’s market/street vendors | >500g | 2015-08-03 | Shanxi | Changzhi city | Show in tunliu County breakfast | Show in tunliu County breakfast | 2015.8.3 |
| SA806 | Rice- and flour-products | Rice- and flour-products | unpacked | farmer’s market/street vendors | >500g | 2015-08-03 | Shanxi | Changzhi city | Ancient Han Zhenxi closed markets in xiangyuan County the snack bar | Ancient Han Zhenxi closed markets in xiangyuan County the snack bar | 2015.8.3 |
| SA807 | Rice- and flour-products | Rice- and flour-products | unpacked | farmer’s market/street vendors | >500g | 2015-08-12 | Shanxi | In Jincheng city | Zezhou County Government assured BA Gong Zhen breakfast 44 Shop | Ekin Cheng of Jincheng city foods limited | 20150812 |
| SA808 | Rice- and flour-products | Rice- and flour-products | unpacked | farmer’s market/street vendors | >500g | 2015-04-27 | Shanxi | Taiyuan | Yijingnan market | Golden soy foods, Ltd | 20150427 |
| SA809 | Rice- and flour-products | Rice- and flour-products | unpacked | farmer’s market/street vendors | >500g | 2015-08-04 | Shanxi | Yangquan city | Bei da Jie Tan Tian Li mining supermarket | Tan Tian Li mining area in yangquan city supermarket | 20150804 |
| SA810 | Rice- and flour-products | Rice- and flour-products | unpacked | farmer’s market/street vendors | >500g | 2015-08-03 | Hebei | Langfang city | Yu Feng Jie, Dongguan Royal lagoon community West of the old snacks | Yu Feng Jie, Dongguan Royal lagoon community West of the old snacks | 2015.8.3 |
| SA811 | Rice- and flour-products | Rice- and flour-products | unpacked | farmer’s market/street vendors | >500g | 2015-08-03 | Hebei | Langfang city | Yi Chang road, East of the East Gate of the industrial and commercial bank recorded breakfast stall | Yi Chang road, East of the East Gate of the industrial and commercial bank recorded breakfast stall | 2015.8.3 |
| SA812 | Rice- and flour-products | Rice- and flour-products | unpacked | farmer’s market/street vendors | >500g | 2015-04-13 | Hebei | Tangshan City | Road star market (Wang) | Star market (Wang) | 2015.4.13 |
| SA813 | Rice- and flour-products | Rice- and flour-products | unpacked | farmer’s market/street vendors | >500g | 2015-07-14 | Hebei | Tangshan City | Guang Ming Nan lu | Guang Ming Nan Lu Yang ladies | 2015.7.14 |
| SA814 | Rice- and flour-products | Rice- and flour-products | unpacked | farmer’s market/street vendors | >500g | 2015-05-25 | Hebei | Tangshan City | XI Hui min Park North exit | North exit of Park of Huimin (Elaine) | 2015.5.25 |
| SA815 | Rice- and flour-products | Rice- and flour-products | unpacked | farmer’s market/street vendors | >500g | 2015-07-14 | Hebei | Tangshan City | Nan Guang Ming Nan lu | Wang Nan Guang Ming Nan lu | 2015.7.14 |
| SA816 | Rice- and flour-products | Rice- and flour-products | unpacked | farmer’s market/street vendors | >500g | 2015-04-13 | Hebei | Zhangjiakou City | Chai Gou Bao Zhen Lu Xinrong pancakes | Huai ' an Chai Gou Bao Zhen Lu Xinrong pancakes | 2015-4-13 |
| SA817 | Rice- and flour-products | Rice- and flour-products | unpacked | farmer’s market/street vendors | >500g | 2015-04-14 | Yunnan | Dali Bai autonomous prefecture | ER he XI Lu, Xia Guan town mark for city farmers ' market 22 Booth | Doll sold | 2015-04-14 |
| SA818 | Rice- and flour-products | Rice- and flour-products | unpacked | farmer’s market/street vendors | >500g | 2015-04-14 | Yunnan | Dali Bai autonomous prefecture | Shimonoseki taixing gate of the farmers ' market stalls | Li Chen produced from | 2015-04-14 |
| SA819 | Rice- and flour-products | Rice- and flour-products | unpacked | farmer’s market/street vendors | >500g | 2015-09-09 | Yunnan | Honghe Hani and Yi Autonomous | Jin hua Lu XING long farmers market district 1 Booth | Mengzi, grain rice noodle factory | 2015.6.23 |
| SA820 | Rice- and flour-products | Rice- and flour-products | unpacked | farmer’s market/street vendors | >500g | 2015-04-27 | Yunnan | Pu-Erh tea | Border town road 10 Number 51 farmers market F Shop early at the gate | Yang Enxiu laboratories | 20150727 |
| SA821 | Rice- and flour-products | Rice- and flour-products | unpacked | farmer’s market/street vendors | >500g | 2015-05-26 | Yunnan | In zhaotong city | Xinglong Jie inflammation mountain grass jelly | Zhaoyang district Zhang Jiqiang inflammation mountain jelly shop | 2015.9.21 |
| SA822 | Rice- and flour-products | Rice- and flour-products | unpacked | farmer’s market/street vendors | >500g | 2015-06-02 | Liaoning | Anshan city | The Ngoi Man Street 7 Snack fat ya | The Ngoi Man Street 7 Snack fat ya | 20150602 |
| SA823 | Rice- and flour-products | Rice- and flour-products | unpacked | farmer’s market/street vendors | >500g | 2015-08-11 | Liaoning | Yingkou city | Huaihe River road sea days before the East Gate market | Unknown | 20151103 |
| SA824 | Rice- and flour-products | Rice- and flour-products | unpacked | farmer’s market/street vendors | >500g | 2015-05-25 | Inner Mongoria | Baotou city | Lin Qi 1 Leisure district, Fu Ping Yuan baked dry goods shop | Fu Ping Yuan dry goods store | 20150525 |
| SA825 | Rice- and flour-products | Rice- and flour-products | unpacked | farmer’s market/street vendors | >500g | 2015-09-28 | Shaanxi | In Ankang city | Bashan mountain road, stone recorded steam heavy hotels | Our shop | 20150928 |
| SA826 | Rice- and flour-products | Rice- and flour-products | unpacked | farmer’s market/street vendors | >500g | 2015-09-28 | Shaanxi | In Ankang city | Dang Xiao Lu Li snack | Our shop | 20150928 |
| SA827 | Rice- and flour-products | Rice- and flour-products | unpacked | farmer’s market/street vendors | >500g | 2015-06-23 | Shaanxi | Baoji city | Shuangshipu farmers market outside the | Unknown | 20150623 |
| SA828 | Rice- and flour-products | Rice- and flour-products | unpacked | farmer’s market/street vendors | >500g | 2015-04-13 | Shaanxi | Hanzhong city | Lu Liu Jia Cheng Gu dough | In-store produced | 2015.4.13 |
| SA829 | Rice- and flour-products | Rice- and flour-products | unpacked | farmer’s market/street vendors | >500g | 2015-04-13 | Shaanxi | Hanzhong city | Public roadway Santan | Santan | 2015.4.13 |
| SA830 | Rice- and flour-products | Rice- and flour-products | unpacked | farmer’s market/street vendors | >500g | 2015-05-18 | Shaanxi | Hanzhong city | Yau Oi Road next to the four street vendors | In-store produced | 2015.5.18 |
| SA831 | Rice- and flour-products | Rice- and flour-products | unpacked | farmer’s market/street vendors | >500g | 2015-06-15 | Shaanxi | Hanzhong city | River Street side of the nameless shop | In-store produced | 2015.6.15 |
| SA832 | Rice- and flour-products | Rice- and flour-products | unpacked | farmer’s market/street vendors | >500g | 2015-06-15 | Shaanxi | Hanzhong city | Lu, a bowl of sweet snacks | In-store produced | 2015.6.15 |
| SA833 | Rice- and flour-products | Rice- and flour-products | unpacked | farmer’s market/street vendors | >500g | 2015-06-15 | Shaanxi | Hanzhong city | Ming Zhong Xiang, a roadside stall | Santan | 2015.6.15 |
| SA834 | Rice- and flour-products | Rice- and flour-products | unpacked | farmer’s market/street vendors | >500g | 2015-06-15 | Shaanxi | Hanzhong city | School lane street | Santan | 2015.6.15 |
| SA835 | Rice- and flour-products | Rice- and flour-products | unpacked | farmer’s market/street vendors | >500g | 2015-06-29 | Shaanxi | Tongchuan city | Red Bridge Zhengda Street mobile stalls 2 | Production | 2015.6.29 |
| SA836 | Rice- and flour-products | Rice- and flour-products | unpacked | farmer’s market/street vendors | >500g | 2015-05-05 | Shaanxi | In weinan city | South pond road stalls ( East) | Owner made | 20150505 |
| SA837 | Rice- and flour-products | Rice- and flour-products | unpacked | farmer’s market/street vendors | >500g | 2015-09-14 | Shaanxi | In weinan city | Town of two-lane alley Santan | Owner production | 20150914 |
| SA838 | Rice- and flour-products | Rice- and flour-products | unpacked | farmer’s market/street vendors | >500g | 2015-05-18 | Shaanxi | XI ' an city | Cun Xi Lu 60 Mobile meals, East House | Cun Xi Lu 60 Mobile meals, East third-home | 2015-5-18 |
| SA839 | Rice- and flour-products | Rice- and flour-products | unpacked | farmer’s market/street vendors | >500g | 2015-08-03 | Shaanxi | XI ' an city | Kitchen in Red Lane Rd group Kim Yun Xiaozhai East Road | Public kitchens | 20150803 |
| SA840 | Rice- and flour-products | Rice- and flour-products | unpacked | farmer’s market/street vendors | >500g | 2015-09-07 | Shaanxi | XI ' an city | Wen Wei Lu 101 Blind farmer's market, East of third | Wen Wei Lu 101 East third self-produced Qiming, farmers ' markets | 20150907 |
| SA841 | Rice- and flour-products | Rice- and flour-products | unpacked | farmer’s market/street vendors | >500g | 2015-05-19 | Shaanxi | Xianyang city | Seven farmers market Fu bing shop | Qindu district, xianyang city, seven factory farmers market Fu bing shop | 20150519 |
| SA842 | Rice- and flour-products | Rice- and flour-products | unpacked | farmer’s market/street vendors | >500g | 2015-09-15 | Shaanxi | Yanan city | Hei Gou Gou Kou Wang | The Royal Army | 20150915 |
| SA843 | Rice- and flour-products | Rice- and flour-products | unpacked | farmer’s market/street vendors | >500g | 2015-09-28 | Shaanxi | Yanan city | Yanan University entrance | Homemade | 2015.9.28 |
| SA844 | Rice- and flour-products | Rice- and flour-products | unpacked | farmer’s market/street vendors | >500g | 2015-09-28 | Shaanxi | Yanan city | Yanan University entrance | Homemade | 2015.9.28 |
| SA845 | Rice- and flour-products | Rice- and flour-products | unpacked | farmer’s market/street vendors | >500g | 2015-08-10 | Fujian | Quanzhou city | Luo Cheng Zhen Jian she computer city, Zhongshan South Road next to the breakfast car (car number XZC-H ) | Xiamen, Huang and food company limited | 20150810 |
| SA846 | Rice- and flour-products | Rice- and flour-products | unpacked | farmer’s market/street vendors | >500g | 2015-06-30 | Fujian | Nanping city | 513 287 Outside the hospital, mobile breakfast cart | Unknown | 2015-06-30 |
| SA847 | Rice- and flour-products | Rice- and flour-products | unpacked | farmer’s market/street vendors | >500g | 2015-07-07 | Fujian | Nanping city | Xu Tong Youzhen Xinhua market breakfast stand | Xu earlier | 2015-07-07 |
| SA848 | Rice- and flour-products | Rice- and flour-products | unpacked | farmer’s market/street vendors | >500g | 2015-08-04 | Fujian | Nanping city | Ying Pan road 1-5 Its first high school entrance | Unknown | 15-8-4 |
| SA849 | Rice- and flour-products | Rice- and flour-products | unpacked | farmer’s market/street vendors | >500g | 2015-06-09 | Hunan | Yiyang city | Autumn fruit Lu Yi Shi Wu across the school owner | Unknown | 20150609 |
| SA850 | Rice- and flour-products | Rice- and flour-products | unpacked | farmer’s market/street vendors | >500g | 2015-06-15 | Shandong | Liaocheng city | New West chicken GAMO booth | Homemade | 15-6-15 |
| SA851 | Rice- and flour-products | Rice- and flour-products | unpacked | farmer’s market/street vendors | >500g | 2015-07-01 | Ningxia | Zhongwei city | Tian ren San Qu 1-1 Business room, Mao Yan Liang PI 麻辣烫 shop | Zhao Liang PI processing shop | 20150701 |
| SA852 | Rice- and flour-products | Rice- and flour-products | unpacked | farmer’s market/street vendors | >500g | 2015-07-14 | Ningxia | In guyuan city | Cultural BREW Street, King of Kings | King of Kings, stuffed skins | 20150714 |
| SA853 | Rice- and flour-products | Rice- and flour-products | unpacked | farmer’s market/street vendors | >500g | 2015-07-07 | Ningxia | In guyuan city | Park West Xin Mei 麻辣烫 shop | The pungent taste spicy soup shop | 20140707 |
| SA854 | Rice- and flour-products | Rice- and flour-products | unpacked | farmer’s market/street vendors | >500g | 2015-07-08 | Ningxia | Yinchuan city | North South Street 61 Hui, cold noodles shop | Hui liang PI shop | 20150708 |
| SA855 | Rice- and flour-products | Rice- and flour-products | unpacked | farmer’s market/street vendors | >500g | 2015-05-20 | Guangdong | Foshan City | Source after the Temple Street intersection in Tianjin baozi shop | Tianjin baozi shop | 20150520 |
| SA856 | Rice- and flour-products | Rice- and flour-products | unpacked | farmer’s market/street vendors | >500g | 2015-05-20 | Guangdong | Foshan City | Dong er road, guicheng Street village near the market entrance mobile stalls | Unknown | 20150520 |
| SA857 | Rice- and flour-products | Rice- and flour-products | unpacked | farmer’s market/street vendors | >500g | 2015-05-20 | Guangdong | Foshan City | Gui Lan Lu Xi guicheng street restaurant | Hi restaurant | 20150520 |
| SA858 | Rice- and flour-products | Rice- and flour-products | unpacked | farmer’s market/street vendors | >500g | 2015-05-28 | Guangdong | In Zhanjiang city | Gou Wei village snack stall | Potou district, Zhanjiang city, GOU Wei village snack stall | 20150528 |
| SA859 | Rice- and flour-products | Rice- and flour-products | unpacked | farmer’s market/street vendors | >500g | 2015-05-18 | Guangdong | Shenzhen | Jing Tian Shui Xin Yuan road 36 Lu Kee noodle house | Lu Kee noodle shop | 20150518 |
| SA860 | Rice- and flour-products | Rice- and flour-products | unpacked | farmer’s market/street vendors | >500g | 2015-08-10 | Guangdong | Zhaoqing city | Da Jing Qiao TOU breakfast street vendors | Unknown | 20150810 |
| SA861 | Rice- and flour-products | Rice- and flour-products | unpacked | farmer’s market/street vendors | >500g | 2015-09-14 | Guangdong | In Zhanjiang city | Haidong market stalls | / | 20150914 |
| SA862 | Rice- and flour-products | Rice- and flour-products | unpacked | farmer’s market/street vendors | >500g | 2015-05-25 | Sichuan | In Mianyang City, Sichuan | Mianzhou road north of Mianyang City 98 Lake station, mobile | Homemade | 20150525 |
| SA863 | Rice- and flour-products | Rice- and flour-products | unpacked | farmer’s market/street vendors | >500g | 2015-05-23 | Sichuan | Panzhihua city | Century city Gimhae airport road mobile stalls (small steamed dish) | Unknown | 2015-05-23 |
| SA864 | Rice- and flour-products | Rice- and flour-products | unpacked | farmer’s market/street vendors | >500g | 2015-05-04 | Sichuan | Yibin city | Junlian Town June North State Road breakfast booth | Sellers made | 2015-05-04 |
| SA865 | Rice- and flour-products | Rice- and flour-products | unpacked | farmer’s market/street vendors | >500g | 2015-05-25 | Sichuan | Zigong city | Lei yan Guo Jia AO farmers ' markets | Lei yan Guo Jia AO, ziliujing district farmers ' markets homemade | 20150525 |
| SA866 | Rice- and flour-products | Rice- and flour-products | unpacked | farmer’s market/street vendors | >500g | 2015-05-25 | Sichuan | Zigong city | Guo Jia Ping ao farmers ' markets | Wang Ping Guo Jia AO, ziliujing district farmers ' markets homemade | 20150525 |
| SA867 | Rice- and flour-products | Rice- and flour-products | unpacked | farmer’s market/street vendors | >500g | 2015-05-25 | Sichuan | Zigong city | Individual five-star shop farmers ' markets stalls | Unknown | 20150525 |
| SA868 | Rice- and flour-products | Rice- and flour-products | unpacked | farmer’s market/street vendors | >500g | 2015-05-25 | Sichuan | Zigong city | Individual five-star shop farmers ' markets stalls | Unknown | 20150525 |
| SA869 | Rice- and flour-products | Rice- and flour-products | unpacked | farmer’s market/street vendors | >500g | 2015-08-13 | Sichuan | Chengdu city | Wang Jia Miss b shop https://item.taobao.com/item.htm?spm=a1z09.2.0.0.tqq7Xe&id=41068486286&_u=8101tner7be0& qq-pf-to=pcqq.c2c | Wang Jia Miss b shop | 20150812 |
| SA870 | Rice- and flour-products | Rice- and flour-products | unpacked | farmer’s market/street vendors | >500g | 2015-09-21 | Heilongjiang | Da hinggan Ling | Chaoyang Lu Jiawang farmers market market crossing 3 Breakfast booth | Chaoyang Lu Jiawang farmers market market crossing 3 Breakfast booth | 2015.09.21 |
| SA871 | Rice- and flour-products | Rice- and flour-products | unpacked | farmer’s market/street vendors | >500g | 2015-06-09 | Heilongjiang | Mudanjiang city | The pedestrian street 85 Clean, cold noodles shop in Shaanxi Province | Street clean cold noodles of Shaanxi branch | 20150609 |
| SA872 | Rice- and flour-products | Rice- and flour-products | unpacked | farmer’s market/street vendors | >500g | 2015-06-09 | Heilongjiang | Suihua city | Huang bei road 366 Jane Zhou road, near seventh | Seventh, Jane Zhou road | 20150609 |
| SA873 | Rice- and flour-products | Rice- and flour-products | unpacked | farmer’s market/street vendors | >500g | 2015-05-06 | Hubei | Jingmen city | Gou Xiang Qi Mengshan in Shandong and Xiangshan interchange grain pancake stalls | Gou Xiang Qi Mengshan in Shandong and Xiangshan interchange grain pancake stalls | 15-5-6 |
| SA874 | Rice- and flour-products | Rice- and flour-products | unpacked | farmer’s market/street vendors | >500g | 2015-04-01 | Hubei | Wuhan City | Green mania is now grinding shop baby rice cereal http://item.taobao.com/item.htm?spm=a1z09.2.9.66.iZuImV&id=38778170701&_u=62dmgcda34b | Our shop | 15-4-1 |
| SA875 | Rice- and flour-products | Rice- and flour-products | unpacked | farmer’s market/street vendors | >500g | 2015-07-15 | Hubei | Wuhan City | Daughter-in-law farm hand-native in Hunan province http://item.taobao.com/item.htm?spm=a1z09.2.9.101.uaIvZN&id=44531380797&_u=d2dmgcd2061 | Our shop | 15-7-4 |
| SA876 | Rice- and flour-products | Rice- and flour-products | unpacked | farmer’s market/street vendors | >500g | 2015-07-15 | Hubei | Wuhan City | Daughter-in-law farm hand-native in Hunan province http://item.taobao.com/item.htm?spm=a1z09.2.9.117.uaIvZN&id=44469430539&_u=d2dmgcdd685 | Our shop | 15-6-27 |
| SA877 | Rice- and flour-products | Rice- and flour-products | unpacked | farmer’s market/street vendors | >500g | 2015-06-08 | Jilin | Tonghua city | Lucky supermarket Guangming road Li Ruixia stand | Good luck supermarket Li Ruixia stand | 15-6-8 |
| SA878 | Rice- and flour-products | Rice- and flour-products | unpacked | farmer’s market/street vendors | >500g | 2015-06-25 | Jilin | White City | Zhenlai County of zhengyang Street South across the Pok Oi Hospital the morning porridge bread fast food restaurants | Morning porridge bread fast food homemade | 2015-6-25 |
| SA879 | Rice- and flour-products | Rice- and flour-products | unpacked | farmer’s market/street vendors | >500g | 2015-09-30 | Jiangxi | Jingdezhen city | 523 factory dormitory community Breakfast restaurants | 523 factory dormitory community Breakfast restaurants | 20150930 |
| SA880 | Rice- and flour-products | Rice- and flour-products | unpacked | farmer’s market/street vendors | >500g | 2015-05-11 | Jiangxi | Jiujiang city | Pu Tang Lu de ' an old dumplings shop | Old dumplings shop | 20150511 |
| SA881 | Rice- and flour-products | Rice- and flour-products | unpacked | farmer’s market/street vendors | >500g | 2015-08-10 | Zhejiang | Huzhou city | Ren Huang Mountain in huzhou city 1143 Heart of the city, the convenience store door Zi rice mobile stalls | Unknown | 20140720 |
| SA882 | Rice- and flour-products | Rice- and flour-products | unpacked | farmer’s market/street vendors | >500g | 2015-09-07 | Zhejiang | Hangzhou City | Taobao: private tailor-made http://item.taobao.com/item.htm?ut_sk=1.VWchdfTp9C0DAGfKQ0ynfgsI_21380790_1444464836.Copy. | Taobao: private tailor-made | 15-9-14 |
| SA883 | Rice- and flour-products | Rice- and flour-products | unpacked | farmer’s market/street vendors | >500g | 2015-09-14 | Zhejiang | In Lishui city | Ancient town of loose State Road, songyang 296 Happy breakfast | Happy breakfast | 15-9-28 |
| SA884 | Rice- and flour-products | Rice- and flour-products | unpacked | farmer’s market/street vendors | >500g | 2015-09-07 | Beijing | Dongcheng District | Goldfish Street North exit and get in the pool (gold fish pond Street, Dongcheng District, North exit) | Goldfish Street North exit and get in the pool (gold fish pond Street, Dongcheng District, North exit) | 20150907 |
| SA885 | Rice- and flour-products | Rice- and flour-products | unpacked | farmer’s market/street vendors | >500g | 2015-06-01 | Beijing | Fangshan district | Taobao Wenzhou seafood http://item.taobao.com/item.htm?spm=a1z09.2.9.48.wfkq2j&id=44258565387&_u=1pjobpf051e | Taobao Wenzhou seafood | 20150531 |
| SA886 | Rice- and flour-products | Rice- and flour-products | unpacked | farmer’s market/street vendors | >500g | 2015-08-18 | Beijing | Xicheng District | Gokokuji snacks ( Northern grass factory shop) | Gokokuji snacks ( Northern grass factory shop) | 20150818 |
| SA887 | Rice- and flour-products | Rice- and flour-products | unpacked | farmer’s market/street vendors | >500g | 2015-08-18 | Beijing | Xicheng District | Gokokuji snacks ( Northern grass factory shop) | Gokokuji snacks ( Northern grass factory shop) | 20150818 |
| SA888 | Rice- and flour-products | Rice- and flour-products | unpacked | farmer’s market/street vendors | >500g | 2015-08-18 | Beijing | Xicheng District | Gokokuji snacks ( Northern grass factory shop) | Gokokuji snacks ( Northern grass factory shop) | 20150818 |
| SA889 | Rice- and flour-products | Rice- and flour-products | unpacked | farmer’s market/street vendors | >500g | 2015-09-21 | Beijing | Xicheng District | Gokokuji snacks ( Huguosi Street) | Gokokuji snacks ( Huguosi Street) | 20150921 |
| SA890 | Rice- and flour-products | Rice- and flour-products | unpacked | farmer’s market/street vendors | >500g | 2015-09-21 | Beijing | Xicheng District | Gokokuji snacks ( Huguosi Street) | Gokokuji snacks ( Huguosi Street) | 20150921 |
| SA891 | Rice- and flour-products | Rice- and flour-products | unpacked | farmer’s market/street vendors | >500g | 2015-08-11 | Beijing | Xicheng District | De Jiao Chang Kou Street, Xicheng District, South | De Jiao Chang Kou Street, Xicheng District South of stalls | 20150811 |
| SA892 | Rice- and flour-products | Rice- and flour-products | unpacked | farmer’s market/street vendors | >500g | 2015-06-01 | Gansu | In Jiuquan city | Deep in the South exit | Unknown | 2015.6.1 |
| SA893 | Rice- and flour-products | Rice- and flour-products | unpacked | farmer’s market/street vendors | >500g | 2015-08-17 | Gansu | Zhangye city | Zhou Zhi LAN stalls (street primary school entrance) | No | 20150817 |
| SA894 | Vegetable salads | Vegetable salads | unpacked | farmer’s market/street vendors | >500g | 2015-07-13 | Anhui | Hefei city | Clarks kitchen meal tangerines micro-signal QLKITCHEN | Clarks kitchen meal tangerines micro-signal QLKITCHEN | 2015-07-13 |
| SA895 | Vegetable salads | Vegetable salads | unpacked | farmer’s market/street vendors | >500g | 2015-07-13 | Anhui | Hefei city | Clarks kitchen meal tangerines micro-signal QLKITCHEN | Clarks kitchen meal tangerines micro-signal QLKITCHEN | 2015-07-13 |
| SA896 | Vegetable salads | Vegetable salads | unpacked | farmer’s market/street vendors | >500g | 2015-08-11 | Shanxi | In Jincheng city | Friendship Street, Gaoping o hotel | O Hotel homemade | 20150811 |
| SA897 | Vegetable salads | Vegetable salads | unpacked | farmer’s market/street vendors | >500g | 2015-08-03 | Shanxi | Luliang city | Gulou street of Xinhua Bookstore 4 Restaurant in the book language workshop | Fenyang calligraphy corner restaurant | 2015-08-03 |
| SA898 | Vegetable salads | Vegetable salads | unpacked | farmer’s market/street vendors | >500g | 2015-08-17 | Shanxi | In yuncheng city | Leisure restaurant | Leisure restaurant | 2015-8-17 |
| SA899 | Vegetable salads | Vegetable salads | unpacked | farmer’s market/street vendors | >500g | 2015-10-13 | Hebei | Langfang city | Xin Hua road 50 Wanda Plaza, 1 Pizza Hut | Xin Hua road 50 Wanda Plaza, 1 Pizza Hut | 20151013 |
| SA900 | Vegetable salads | Vegetable salads | unpacked | farmer’s market/street vendors | >500g | 2015-08-18 | Hebei | In Qinhuangdao city | Cat tea restaurant (new world, second floor) | Cat restaurant | 2015.8.18 |
| SA901 | Vegetable salads | Vegetable salads | unpacked | farmer’s market/street vendors | >500g | 2015-10-12 | Hebei | Tangshan City | Lubei district Tangshan City, Hebei province, Dali road love coffee | Lubei district Tangshan City, Hebei province, Dali road love coffee | 2015.10.12 |
| SA902 | Vegetable salads | Vegetable salads | unpacked | Supermarket/department stores | >500g | 2015-08-10 | Hebei | Xingtai city | People Street 560 Home, paradise supermarket | Unknown | 15-8-10 |
| SA903 | Vegetable salads | Vegetable salads | unpacked | farmer’s market/street vendors | >500g | 2015-04-21 | Hebei | Zhangjiakou City | Taobao Beijing South Cook http://item.taobao.com/item.htm?spm=a1z09.2.9.115.EgbaMk&id=41312597815&_u=t4tlh9k25d3 | Beijing Cargo homes head office | 2015-4-21 |
| SA904 | Vegetable salads | Vegetable salads | unpacked | farmer’s market/street vendors | >500g | 2015-07-02 | Yunnan | Chuxiong Yi Autonomous Prefecture | Yi Town IV D150-2 South Korean Han Bei street restaurant | Yi Town IV D150-2 South Korean Han Bei street restaurant | 2015-7-2 |
| SA905 | Vegetable salads | Vegetable salads | unpacked | farmer’s market/street vendors | >500g | 2015-10-08 | Yunnan | Chuxiong Yi Autonomous Prefecture | Federation Street 48 Greek restaurant | Federation Street 48 Greek restaurant | 2015-10-8 |
| SA906 | Vegetable salads | Vegetable salads | unpacked | farmer’s market/street vendors | >500g | 2015-10-26 | Yunnan | Lincang city | Hundreds of tree place 5 幢一号牛排西餐厅 | Linxiang district brilliance tree Plaza 5 幢一号牛排西餐厅 | 2015.10.26 |
| SA907 | Vegetable salads | Vegetable salads | unpacked | farmer’s market/street vendors | >500g | 2015-10-26 | Yunnan | Lincang city | Simon lane 63 Old houses, tea bar | Linxiang district, Simon lane 63 Old houses, tea bar | 2015.3.31 |
| SA908 | Vegetable salads | Vegetable salads | unpacked | Supermarket/department stores | >500g | 2015-08-17 | Liaoning | Anshan city | Victory road 42 Head Office of the new Mart supermarket | Victory road 42 Head Office of the new Mart supermarket | 20150817 |
| SA909 | Vegetable salads | Vegetable salads | unpacked | Supermarket/department stores | >500g | 2015-08-10 | Liaoning | Yingkou city | Parkway Middle Xinglong store supermarket | Unknown | 20151103 |
| SA910 | Vegetable salads | Vegetable salads | unpacked | farmer’s market/street vendors | >500g | 2015-05-04 | Liaoning | Dalian City | Changchun Road West 79 Trade building, | No | 20150504 |
| SA911 | Vegetable salads | Vegetable salads | unpacked | farmer’s market/street vendors | >500g | 2015-08-24 | Shaanxi | In Ankang city | Wenchang road Island coffee shop | Our shop | 2015.8.24 |
| SA912 | Vegetable salads | Vegetable salads | unpacked | farmer’s market/street vendors | >500g | 2015-08-24 | Shaanxi | In Ankang city | Wenchang road Island coffee shop | Our shop | 2015.8.24 |
| SA913 | Vegetable salads | Vegetable salads | unpacked | farmer’s market/street vendors | >500g | 2015-10-19 | Shaanxi | In Ankang city | Xingan road on both sides of the coffee shop | Our shop | 20151019 |
| SA914 | Vegetable salads | Vegetable salads | unpacked | farmer’s market/street vendors | >500g | 2015-10-19 | Shaanxi | In Ankang city | Xingan road on both sides of the coffee shop | Our shop | 20151019 |
| SA915 | Vegetable salads | Vegetable salads | unpacked | farmer’s market/street vendors | >500g | 2015-10-19 | Shaanxi | In Ankang city | Xingan road on both sides of the coffee shop | Our shop | 20151019 |
| SA916 | Vegetable salads | Vegetable salads | unpacked | farmer’s market/street vendors | >500g | 2015-11-09 | Shaanxi | In Ankang city | Liberation road Island coffee shop | Our shop | 2015.11.9 |
| SA917 | Vegetable salads | Vegetable salads | unpacked | farmer’s market/street vendors | >500g | 2015-07-20 | Shaanxi | Baoji city | Hawker Beechcraft Qing Jiang steak shop | Hawker Beechcraft Qing Jiang steak shop | 20150720 |
| SA918 | Vegetable salads | Vegetable salads | unpacked | farmer’s market/street vendors | >500g | 2015-09-08 | Shaanxi | XI ' an city | Accordion-style restaurant, Xingqing Road branch | Production | 2015.09.08 |
| SA919 | Vegetable salads | Vegetable salads | unpacked | farmer’s market/street vendors | >500g | 2015-09-08 | Shaanxi | XI ' an city | Fly like a pizza, stadium shop | Production | 2015.09.08 |
| SA920 | Vegetable salads | Vegetable salads | unpacked | farmer’s market/street vendors | >500g | 2015-09-08 | Shaanxi | XI ' an city | Li Jia Cun, Wanda Starlight restaurant | Production | 2015.09.08 |
| SA921 | Vegetable salads | Vegetable salads | unpacked | farmer’s market/street vendors | >500g | 2015-09-08 | Shaanxi | XI ' an city | Wanda Peterborough folk music Park Western restaurant | Production | 2015.09.08 |
| SA922 | Vegetable salads | Vegetable salads | unpacked | farmer’s market/street vendors | >500g | 2015-09-08 | Shaanxi | XI ' an city | Wanda Peterborough folk music Park Western restaurant | Production | 2015.09.08 |
| SA923 | Vegetable salads | Vegetable salads | unpacked | farmer’s market/street vendors | >500g | 2015-10-12 | Shaanxi | XI ' an city | Huangpu magnet coffee bar | Production | 2015.10.12 |
| SA924 | Vegetable salads | Vegetable salads | unpacked | farmer’s market/street vendors | >500g | 2015-10-12 | Shaanxi | XI ' an city | Name code coffee high-tech shop | Production | 2015.10.12 |
| SA925 | Vegetable salads | Vegetable salads | prepacked | farmer’s market/street vendors | >500g | 2015-05-31 | Shaanxi | XI ' an city | Taobao Flower brothers gourmet shop http://item. Taobao. com/item. htm? SPM=a1z09.2.9.26.80Ae8Z& ID=45131827765 &_u=22bcqmbab0eb | Taobao is the flower brothers gourmet shop-made | 2015-5-29 |
| SA926 | Vegetable salads | Vegetable salads | prepacked | farmer’s market/street vendors | >500g | 2015-07-27 | Shaanxi | XI ' an city | Taobao to hang around the kitchen https://item.taobao.com/item.htm?spm=a1z09.2.9.32.xIgae4&id=43722939227&_u=s2bcqmba131e | Taobao: hang around the kitchen homemade | 20150727 |
| SA927 | Vegetable salads | Vegetable salads | unpacked | farmer’s market/street vendors | >500g | 2015-08-17 | Shaanxi | XI ' an city | Shaanxi Golden Bridge international hotel Kunming road sunshine restaurant | Shaanxi Golden Bridge international hotel Kunming road sunshine restaurant | 20150817 |
| SA928 | Vegetable salads | Vegetable salads | unpacked | farmer’s market/street vendors | >500g | 2015-09-07 | Shaanxi | XI ' an city | Xian big wild goose pagoda Plaza (Southeast corner) Tang dynasty never sleeps A9 Yu hua restaurant | Xian big wild goose pagoda Plaza (Southeast corner) Tang dynasty never sleeps A9 Yu hua restaurant | 20150907 |
| SA929 | Vegetable salads | Vegetable salads | unpacked | farmer’s market/street vendors | >500g | 2015-10-19 | Shaanxi | XI ' an city | Qujiang mercy West Road, Qin, Han and Tang Plaza C 2 Loudeshou Palace | Deoksugung Palace | 20151019 |
| SA930 | Vegetable salads | Vegetable salads | unpacked | farmer’s market/street vendors | >500g | 2015-11-02 | Shaanxi | XI ' an city | Wild goose South 2 Silver road north of Cathay Pacific City Pizza Hut | Pizza Hut | 20151102 |
| SA931 | Vegetable salads | Vegetable salads | unpacked | farmer’s market/street vendors | >500g | 2015-10-26 | Shaanxi | Xianyang city | Manny Middle Renmin road coffee shop | Manny Middle Renmin road, Weicheng district coffee shop | 20151026 |
| SA932 | Vegetable salads | Vegetable salads | unpacked | farmer’s market/street vendors | >500g | 2015-10-26 | Shaanxi | Xianyang city | Manny Middle Renmin road coffee shop | Manny Middle Renmin road, Weicheng district coffee shop | 20151026 |
| SA933 | Vegetable salads | Vegetable salads | unpacked | farmer’s market/street vendors | >500g | 2015-10-26 | Shaanxi | Xianyang city | Wei yang Zhong Road 215 Sciano Restaurant opposite the hospital shop | Wei yang Zhong Road 215 Sciano Restaurant opposite the hospital shop | 20151026 |
| SA934 | Vegetable salads | Vegetable salads | unpacked | farmer’s market/street vendors | >500g | 2015-08-06 | Hunan | Xiangtan City | Station camp step by step building of road infrastructure the people of Guilin restaurant | Guilin people dining homemade | 20150806 |
| SA935 | Vegetable salads | Vegetable salads | unpacked | farmer’s market/street vendors | >500g | 2015-05-25 | Hunan | Yiyang city | Find Golden Hill Road | Unknown | 20150525 |
| SA936 | Vegetable salads | Vegetable salads | unpacked | farmer’s market/street vendors | >500g | 2015-09-06 | Hunan | Yiyang city | Peach blossom Island Road West 695 Yiyang, a little sweet fruit store | Yiyang, a little sweet fruit store | 20150906 |
| SA937 | Vegetable salads | Vegetable salads | unpacked | farmer’s market/street vendors | >500g | 2015-05-04 | Hunan | Loudi city | Hunan food http://item.taobao.com/item.htm?cpp=1&id=44215639375&sourceType=item&_navigation_params=%7B" needdismiss"%3A1%7D | Unknown | 20150525 |
| SA938 | Vegetable salads | Vegetable salads | unpacked | farmer’s market/street vendors | >500g | 2015-07-29 | Hunan | In Yongzhou city | DAO Xiao Shui Zhong Lu 161 Xiaoxiang commercial pedestrian street, third floor, James m coffee | Production | 2015.07.29 |
| SA939 | Vegetable salads | Vegetable salads | unpacked | farmer’s market/street vendors | >500g | 2015-09-21 | Hunan | Zhuzhou city | Royal Road 79 Xiang Fu building | Homemade | 20150921 |
| SA940 | Vegetable salads | Vegetable salads | unpacked | farmer’s market/street vendors | >500g | 2015-08-31 | Shandong | Binzhou city | Bohai 11 all the way south of the Yangtze River Dr, bincheng district farmers ' market | Bohai 11 all the way south of the Yangtze River Dr, bincheng district farmers ' market | 2015.08.31 |
| SA941 | Vegetable salads | Vegetable salads | unpacked | farmer’s market/street vendors | >500g | 2015-10-19 | Shandong | Zibo city | Willow Springs Road 152 Maoye Jasmine restaurant | Unknown | 2015.07.29 |
| SA942 | Vegetable salads | Vegetable salads | unpacked | farmer’s market/street vendors | >500g | 2015-07-01 | Ningxia | Zhongwei city | Drum tower Street West of the CRV love to win stylish restaurant | Love to win stylish restaurant | 20150701 |
| SA943 | Vegetable salads | Vegetable salads | unpacked | farmer’s market/street vendors | >500g | 2015-07-06 | Shanghai | Xuhui district, | Salia tianlin road (tianlin road 124 2 Building) | Salia tianlin | 15-7-6 |
| SA944 | Vegetable salads | Vegetable salads | unpacked | farmer’s market/street vendors | >500g | 2015-07-06 | Shanghai | Xuhui district, | COSTA Shanghai tianlin (Guilin road 402 Number) | Agricultural development zone of Jiangsu Chang Le Zhen | 15-7-6 |
| SA945 | Vegetable salads | Vegetable salads | unpacked | farmer’s market/street vendors | >500g | 2015-04-20 | Guangdong | Shanwei city | Zhen a Sunshine market | Unknown | 2015.07.29 |
| SA946 | Vegetable salads | Vegetable salads | unpacked | farmer’s market/street vendors | >500g | 2015-08-09 | Sichuan | Chengdu city | Yuan Wei Bo Bo chickens https://item.taobao.com/item.htm?spm=a1z09.2.0.0.tqq7Xe&id=39471256031&_u=8101tner9f13& qq-pf-to=pcqq.c2c | Yuan Mei Bo Bo chickens | 20150808 |
| SA947 | Vegetable salads | Vegetable salads | unpacked | farmer’s market/street vendors | >500g | 2015-10-28 | Heilongjiang | Heihe city | Dong Xing Road 22 Maria, cold Office | Maria drink, heihe City Hall | 20151028 |
| SA948 | Vegetable salads | Vegetable salads | unpacked | farmer’s market/street vendors | >500g | 2015-08-04 | Heilongjiang | Hegang city | Roman imperial fashion, coal City Road bar | Roman Emperor stylish café-bar | 2015.8.4 |
| SA949 | Vegetable salads | Vegetable salads | unpacked | farmer’s market/street vendors | >500g | 2015-06-02 | Heilongjiang | In jiamusi city | NET world http://item.taobao.com/item.htm?spm=a1z09.2.9.31.NoLZwX&id=37083821448&_u=k1qb46f06cd | Unknown | 20150602 |
| SA950 | Vegetable salads | Vegetable salads | unpacked | farmer’s market/street vendors | >500g | 2015-11-05 | Heilongjiang | In jixi city | Charlie Brown coffee shop | Charlie Brown coffee shop made | 20151105 |
| SA951 | Vegetable salads | Vegetable salads | unpacked | farmer’s market/street vendors | >500g | 2015-11-05 | Heilongjiang | In jixi city | Charlie Brown coffee shop | Charlie Brown coffee shop made | 20151105 |
| SA952 | Vegetable salads | Vegetable salads | unpacked | farmer’s market/street vendors | >500g | 2015-11-05 | Heilongjiang | In jixi city | Floor new building: Barbara pisaniu exhaust shop | Barbara pisaniu rows of shops on the six floor of new building homemade | 20151105 |
| SA953 | Vegetable salads | Vegetable salads | unpacked | farmer’s market/street vendors | >500g | 2015-11-16 | Heilongjiang | Qiqihar | Yongqing market back South 200 Rice, red windmill restaurant | Red windmill restaurant | 20151116 |
| SA954 | Vegetable salads | Vegetable salads | unpacked | farmer’s market/street vendors | >500g | 2015-11-16 | Heilongjiang | Qiqihar | Xin Jiang Lu 9 Road, Wanda Plaza, 3 Floor, town | Italy town | 20151116 |
| SA955 | Vegetable salads | Vegetable salads | unpacked | farmer’s market/street vendors | >500g | 2015-07-20 | Heilongjiang | Qitaihe city | Xuefu road yushun Han jade village a fast food restaurant | Qitaihe city, Heilongjiang province, yushun Han jade village a fast food restaurant | 20150720 |
| SA956 | Vegetable salads | Vegetable salads | unpacked | farmer’s market/street vendors | >500g | 2015-08-11 | Henan | Puyang city | Changqing road 81 Hao, Chinese and Western restaurants | Chang Qing road, Puyang 81 Hao, Chinese and Western restaurants | 2015-08-11 |
| SA957 | Vegetable salads | Vegetable salads | unpacked | farmer’s market/street vendors | >500g | 2015-08-10 | Henan | Puyang city | Construction of road 272 Good thinking good restaurants | Area road, Puyang 272 Good thinking good restaurants | 2015-08-10 |
| SA958 | Vegetable salads | Vegetable salads | unpacked | farmer’s market/street vendors | >500g | 2015-08-10 | Henan | Puyang city | Zhou Lu, central square of Kentucky Center | Zhou Lu district, Puyang City Center Plaza KFC Center shop | 2015-08-10 |
| SA959 | Vegetable salads | Vegetable salads | unpacked | farmer’s market/street vendors | >500g | 2015-07-06 | Hubei | Wuhan City | Peace Avenue 809 Okuyama, Century Plaza 3 Okuyama louxiuyu square shop | Our shop | 20150706 |
| SA960 | Vegetable salads | Vegetable salads | unpacked | farmer’s market/street vendors | >500g | 2015-07-08 | Jilin | Tonghua city | Europe and Asia Shuang he Sheng Guangming road cafeteria | Europe and Asia Shuang he Sheng cafeteria | 15-7-8 |
| SA961 | Vegetable salads | Vegetable salads | unpacked | farmer’s market/street vendors | >500g | 2015-08-18 | Jilin | Jilin city | Benning alley Grill sausage morning Jane and the city | Benning alley Grill sausage morning Jane and the city | 15-8-18 |
| SA962 | Vegetable salads | Vegetable salads | prepacked | Supermarket/department stores | >500g | 2015-06-29 | Jiangxi | Fuzhou City | To buy (lean shop) | Jiangxi yinqiao vegetables industry limited | 20150424 |
| SA963 | Vegetable salads | Vegetable salads | unpacked | farmer’s market/street vendors | >500g | 2015-06-29 | Jiangxi | Fuzhou City | City Farmer's market (Shunda seasoning) | Unknown | 2015.07.29 |
| SA964 | Vegetable salads | Vegetable salads | unpacked | farmer’s market/street vendors | >500g | 2015-06-29 | Jiangxi | Fuzhou City | City Farmer's market (Shunda seasoning) | Jiangxi nanfeng Orange peak vegetable processing plant | 20150214 |
| SA965 | Vegetable salads | Vegetable salads | unpacked | farmer’s market/street vendors | >500g | 2015-08-24 | Jiangxi | Jingdezhen city | On both sides of the coffee | On both sides of the coffee | 20150824 |
| SA966 | Vegetable salads | Vegetable salads | unpacked | farmer’s market/street vendors | >500g | 2015-08-18 | Jiangxi | Shangrao city | Poly long bridge futai Plaza, | Statue of steak | 20150817 |
| SA967 | Vegetable salads | Vegetable salads | unpacked | farmer’s market/street vendors | >500g | 2015-09-14 | Jiangxi | Xinyu city | Fenyi Shuang Lin hotel | Fenyi shuanglin hotel (Station Road) | 20150910 |
| SA968 | Vegetable salads | Vegetable salads | unpacked | farmer’s market/street vendors | >500g | 2015-11-16 | Jiangxi | Yichun city | Fengxin Ziyun fengchuanzhen Longshan Avenue Court 103 Doraemon A Dream music restaurant | Fengxin Ziyun fengchuanzhen Longshan Avenue Court 103 Doraemon A Dream music restaurant | 2015.11.16 |
| SA969 | Vegetable salads | Vegetable salads | unpacked | farmer’s market/street vendors | >500g | 2015-09-06 | Zhejiang | Hangzhou City | Micro store: a box of salad; micro-signals: yihesl | Micro-credit services number: a box of salad; micro-signals: yihesl | 15-9-6 |
| SA970 | Vegetable salads | Vegetable salads | unpacked | farmer’s market/street vendors | >500g | 2015-09-14 | Zhejiang | Hangzhou City | Micro store: a grain of millet creative sushi-baked rice http://weidian.com/item.html?itemID=1459680128&appid=com.koudai.weidian.buyer&wfr=vdbuyer!h5!item !c!share | Micro store: a grain of millet creative sushi fried rice | 20150824 |
| SA971 | Vegetable salads | Vegetable salads | unpacked | farmer’s market/street vendors | >500g | 2015-09-14 | Zhejiang | Zhoushan City | Zhoushan City Island Road 156 Blue Demon Fang, xincheng Hotel | Zhoushan City Island Road 156 Blue Demon Fang, xincheng Hotel | 2015-09-14 |
| SA972 | Vegetable salads | Vegetable salads | unpacked | farmer’s market/street vendors | >500g | 2015-09-06 | Xinjiang | Urumqi city | Beijing Road, 895 Second upstairs building railways, island coffee | UCC | 20150906 |
| SA973 | Vegetable salads | Vegetable salads | unpacked | farmer’s market/street vendors | >500g | 2015-10-20 | Xinjiang | Urumqi city | Lu Chengji Urumqi construction building 1,2 Building the Hermitage Pavilion coffee shop | Lu Chengji Urumqi construction building 1,2 Building the Hermitage Pavilion coffee shop | 2015-10-20 |
| SA974 | Vegetable salads | Vegetable salads | unpacked | farmer’s market/street vendors | >500g | 2015-07-09 | Xinjiang | Karamay city | Karamay Island coffee | District, island coffee | 20150709 |
| SA975 | Vegetable salads | Vegetable salads | unpacked | farmer’s market/street vendors | >500g | 2015-10-30 | Beijing | Changping District | Beijing longmai hot spring resorts rose restaurant ( Willow village of xiaotangshan town, Changping District, Beijing) | Beijing longmai hot spring resort | 20151030 |
| SA976 | Vegetable salads | Vegetable salads | unpacked | farmer’s market/street vendors | >500g | 2015-09-07 | Beijing | Dongcheng District | Nanwei road 2 12 ( Bridge Mall to the West 200 meters | Sushi restaurant foods limited, Beijing Tianqiao (bridge Mall to the West 200 M) | 20150907 |
| SA977 | Vegetable salads | Vegetable salads | unpacked | farmer’s market/street vendors | >500g | 2015-09-06 | Gansu | Tianshui city | North Central Square public road 1 2 Loushengan coffee shop II branch | Production | 20150906 |
| SA978 | Vegetable salads | Vegetable salads | unpacked | farmer’s market/street vendors | >500g | 2015-09-06 | Gansu | Tianshui city | North Central Square public road 1 2 Loushengan coffee shop II branch | Production | 20150906 |
| SA979 | Sandwich | Sandwich | prepacked | farmer’s market/street vendors | >500g | 2015-06-29 | Anhui | Hefei city | Western Red tomatoes http://hfhongfanqie.taobao.com/ | Western Red tomatoes http://hfhongfanqie.taobao.com/ | 20150629 |
| SA980 | Sandwich | Sandwich | unpacked | farmer’s market/street vendors | >500g | 2015-06-29 | Anhui | Hefei city | Lixin road, Fengtai road intersection thousand jida hair shop | Anhui auspicious foods, Ltd | 20150628 |
| SA981 | Sandwich | Sandwich | unpacked | farmer’s market/street vendors | >500g | 2015-06-29 | Anhui | Hefei city | Tai Hu Lu Wanzhen carefree garden next to the South Gate of Qian Ji's bakery | Anhui auspicious foods, Ltd | 20150628 |
| SA982 | Sandwich | Sandwich | unpacked | farmer’s market/street vendors | >500g | 2015-07-20 | Anhui | Hefei city | Perfect square next to the experimental kindergarten West Park village West Park branch | Perfect square West Park branch | 2015-07-20 |
| SA983 | Sandwich | Sandwich | unpacked | farmer’s market/street vendors | >500g | 2015-07-20 | Anhui | Hefei city | Road and Park Lane cooperative road junction in Guichi remember Guichi road | Anhui Pak Siu Kee food company limited | 2015-07-20 |
| SA984 | Sandwich | Sandwich | prepacked | farmer’s market/street vendors | >500g | 2015-07-20 | Anhui | Hefei city | Lakefront scholar shops C 106 Maiqika bakery | Maiqika bakery | 2015-07-19 |
| SA985 | Sandwich | Sandwich | unpacked | farmer’s market/street vendors | >500g | 2015-08-24 | Anhui | Hefei city | Suzhou road junction over the pinch of orogenic lane Hong Kong bakery | Super foods limited liability company | 20150824 |
| SA986 | Sandwich | Sandwich | unpacked | farmer’s market/street vendors | >500g | 2015-07-15 | Shanxi | In Jincheng city | Meite thinking cake, Jincheng city Shanxi steel shop | Special thought cake shops | 2015-07-15 |
| SA987 | Sandwich | Sandwich | unpacked | farmer’s market/street vendors | >500g | 2015-08-12 | Shanxi | In Jincheng city | Huang Hua Street, Jincheng city ports cake shop | Ports to cake-homemade | 20150812 |
| SA988 | Sandwich | Sandwich | unpacked | farmer’s market/street vendors | >500g | 2015-06-07 | Hebei | Cangzhou city | Yuhua road on both sides of the coffee shop | On both sides of the coffee shop | 20150607 |
| SA989 | Sandwich | Sandwich | unpacked | farmer’s market/street vendors | >500g | 2015-08-04 | Hebei | Cangzhou city | KFC takuhaibin ( https://www.4008823823.com.cn/kfcios/Html/index.html ) | Cangzhou city Kentucky | 2015.8.1 |
| SA990 | Sandwich | Sandwich | unpacked | farmer’s market/street vendors | >500g | 2015-08-04 | Hebei | Cangzhou city | Mall goldlion bakery | Goldlion bakery | 2015.8.4 |
| SA991 | Sandwich | Sandwich | unpacked | farmer’s market/street vendors | >500g | 2015-08-10 | Hebei | Tangshan City | Mr John Cha Yuan Jie, Yu Tian Qiao North Road, Tangshan City | Unknown | 2015.8.10 |
| SA992 | Sandwich | Sandwich | unpacked | farmer’s market/street vendors | >500g | 2015-08-10 | Hebei | Tangshan City | Mr John Cha Yuan Jie, Yu Tian Qiao North Road, Tangshan City | Unknown | 2015.8.10 |
| SA993 | Sandwich | Sandwich | prepacked | Supermarket/department stores | >500g | 2015-07-13 | Hebei | Tangshan City | Octagon shopping mall department store in Tangshan group limited liability company | Beijing peach food co | 2015.7.12 |
| SA994 | Sandwich | Sandwich | prepacked | farmer’s market/street vendors | >500g | 2015-07-06 | Hebei | Tangshan City | Taobao Ding food https://item.taobao.com/item.htm?spm=a1z10.3-c.w4002-81306 | Morita food Jiangsu co | 2015.03.11 |
| SA995 | Sandwich | Sandwich | prepacked | farmer’s market/street vendors | >500g | 2015-07-13 | Hebei | Tangshan City | Taobao fool supermarket https://item.taobao.com/item.htm?spm=a1z10.3-c.w4002-4294565758.14.q65WEO | Hebei-want foods limited | 2015.04.9 |
| SA996 | Sandwich | Sandwich | unpacked | farmer’s market/street vendors | >500g | 2015-07-20 | Yunnan | Lijiang city | Xin Yi Jie Yu Ho square Pizza Hut the Jade River, Dayan Office Plaza | Yu he Plaza old town Pizza Hut restaurant | 20150605 |
| SA997 | Sandwich | Sandwich | unpacked | farmer’s market/street vendors | >500g | 2015-07-20 | Yunnan | Qujing city | Rui and Xin Yi fang XI Yuan, South Branch | Zhanyi County longhuaxinyi square food factory | 20150630 |
| SA998 | Sandwich | Sandwich | unpacked | farmer’s market/street vendors | >500g | 2015-07-01 | Liaoning | In Fushun city | Xinhua Street 5 Gelanxi (new store) | Gelanxi (new store) | 20150706 |
| SA999 | Sandwich | Sandwich | prepacked | Supermarket/department stores | >500g | 2015-08-17 | Inner Mongoria | Hohhot | Ordos main street 26 Beijing Hualian supermarket (Inner Mongolia jinyu store) | Beijing wheat delicious food Corporation Hohhot branch | 20150815 |
| SA1000 | Sandwich | Sandwich | prepacked | Supermarket/department stores | >500g | 2015-07-04 | Inner Mongoria | Baotou city | Tai Nan Street 8 Insein, supermarket | Inner Mongolia peach food co | 20150703 |
| SA1001 | Sandwich | Sandwich | unpacked | farmer’s market/street vendors | >500g | 2015-06-11 | Inner Mongoria | ALXA League | Italian jazz Bayanhaote Town jilantai road coffee shop | Italian jazz Bayanhaote Town jilantai road coffee shop | 20150611 |
| SA1002 | Sandwich | Sandwich | unpacked | farmer’s market/street vendors | >500g | 2015-06-11 | Inner Mongoria | Bayan Nur city | Victory road on the Island Cafe | Island coffee | 2015.06.11 |
| SA1003 | Sandwich | Sandwich | unpacked | farmer’s market/street vendors | >500g | 2015-06-15 | Shaanxi | In Ankang city | Yucai road of Maggie West bakery | Our shop | 2015.6.15 |
| SA1004 | Sandwich | Sandwich | prepacked | farmer’s market/street vendors | >500g | 2015-08-04 | Shaanxi | Baoji city | Yu Xuan fruit birthday cake | Yu Xuan fruit birthday cake | 20150720 |
| SA1005 | Sandwich | Sandwich | unpacked | farmer’s market/street vendors | >500g | 2015-06-16 | Shaanxi | Xianyang city | BA Fulin Taiwan factory gate PA, Renmin road, bei classic cake shop | BA Fulin Taiwan factory gate PA, Renmin road, bei classic cake shop | 20150616 |
| SA1006 | Sandwich | Sandwich | unpacked | farmer’s market/street vendors | >500g | 2015-08-25 | Shaanxi | Xianyang city | ANDing road European AI cake shop | ANDing road European AI cake shop | 20150825 |
| SA1007 | Sandwich | Sandwich | unpacked | farmer’s market/street vendors | >500g | 2015-06-24 | Shaanxi | Yanan city | Erdao Jie hollyland | Erdao Jie hollyland | 20150624 |
| SA1008 | Sandwich | Sandwich | unpacked | farmer’s market/street vendors | >500g | 2015-06-24 | Shaanxi | Yanan city | Centre Street rule to be fair to coast | Ping building ports | 20150624 |
| SA1009 | Sandwich | Sandwich | unpacked | farmer’s market/street vendors | >500g | 2015-06-24 | Shaanxi | Yanan city | Centre Street rule to be fair to coast | Ping building ports | 20150624 |
| SA1010 | Sandwich | Sandwich | prepacked | farmer’s market/street vendors | >500g | 2015-08-03 | Shaanxi | Yanan city | Center Street in McCormick | McCormick West bakery | 20150803 |
| SA1011 | Sandwich | Sandwich | unpacked | farmer’s market/street vendors | >500g | 2015-08-03 | Shaanxi | Yanan city | Hollyland Luo ping's shop | Olivetti | 20150803 |
| SA1012 | Sandwich | Sandwich | unpacked | farmer’s market/street vendors | >500g | 2015-08-03 | Shaanxi | Yanan city | Luo Jia ping Xinzhou garden ports | Ports | 20150803 |
| SA1013 | Sandwich | Sandwich | unpacked | farmer’s market/street vendors | >500g | 2015-08-03 | Shaanxi | Yanan city | Todai still tastes good | Todai still tastes good | 20150803 |
| SA1014 | Sandwich | Sandwich | unpacked | farmer’s market/street vendors | >500g | 2015-06-29 | Fujian | Nanping city | Seven degrees bake shunchang branch (County 12-13 Number) | Seven degrees bake shunchang branch | 2015-06-29 |
| SA1015 | Sandwich | Sandwich | unpacked | farmer’s market/street vendors | >500g | 2015-07-07 | Fujian | Nanping city | Tong Youzhen song CI road 23 Bread cake, beautiful city | Jianyang city of beauty and good bread cake boy swim shop | 2015-07-06 |
| SA1016 | Sandwich | Sandwich | unpacked | farmer’s market/street vendors | >500g | 2015-08-04 | Fujian | Nanping city | Tong Youzhen song CI road 51 Cocoa cake shop | Cocoa cake shop | 15-8-3 |
| SA1017 | Sandwich | Sandwich | unpacked | farmer’s market/street vendors | >500g | 2015-09-06 | Hunan | Yiyang city | North Road rehabilitation 265 Wheat source recovery shop | North Road rehabilitation 265 Wheat source recovery shop | 20150906 |
| SA1018 | Sandwich | Sandwich | unpacked | farmer’s market/street vendors | >500g | 2015-09-06 | Hunan | Yiyang city | North Road rehabilitation 2 Wheat source recovery shop | North Road rehabilitation 2 Hua Tian, business building, first floor | 20150906 |
| SA1019 | Sandwich | Sandwich | unpacked | farmer’s market/street vendors | >500g | 2015-06-03 | Hunan | Xiangxi Tujia and Miao autonomous | Mengdonghe mengdonghe Rd West bakery | Mengdonghe West bakery | 20150603 |
| SA1020 | Sandwich | Sandwich | unpacked | farmer’s market/street vendors | >500g | 2015-06-15 | Shandong | Rizhao city | Junction city, Zibo road and Northwest 36 9 Bakery | 36 9 Baking | 15-6-15 |
| SA1021 | Sandwich | Sandwich | unpacked | farmer’s market/street vendors | >500g | 2015-06-15 | Shandong | Rizhao city | Yellow Sea all the way across the city bakery | Excellent bakery | 15-6-15 |
| SA1022 | Sandwich | Sandwich | prepacked | Supermarket/department stores | >500g | 2015-06-08 | Shandong | In linyi city | Zhongshan North Road, linshu Zhengda suguo supermarkets | Linshu County D-5 Elements restaurant | 15-6-8 |
| SA1023 | Sandwich | Sandwich | prepacked | Supermarket/department stores | >500g | 2015-06-08 | Shandong | In linyi city | Zhongshan North Road, linshu Zhengda suguo supermarkets | Linshu County D-5 Elements restaurant | 15-6-8 |
| SA1024 | Sandwich | Sandwich | unpacked | farmer’s market/street vendors | >500g | 2015-08-10 | Shandong | Rizhao city | Paris bakery RT shop | Paris bakery | 15-8-10 |
| SA1025 | Sandwich | Sandwich | unpacked | farmer’s market/street vendors | >500g | 2015-08-10 | Shandong | Rizhao city | Best cake shop on the University City School Road | Best cake shop | 15-8-10 |
| SA1026 | Sandwich | Sandwich | unpacked | farmer’s market/street vendors | >500g | 2015-06-12 | Shanghai | Xuhui district, | Chung fang damuqiao road (damuqiao road, xuhui district, Shanghai 509 Number) | Shanghai Chung fang food industry limited company | 15-6-12 |
| SA1027 | Sandwich | Sandwich | unpacked | farmer’s market/street vendors | >500g | 2015-06-30 | Guangdong | Shenzhen | Golden Bamboo Road Park 26 Bread to accompany your bamboo shop, shop | Bread to accompany your bamboo shop | 20150630 |
| SA1028 | Sandwich | Sandwich | unpacked | farmer’s market/street vendors | >500g | 2015-07-13 | Guangdong | Foshan City | Penglai road, Daliang 2 Mary a Crown bakery | Penglai road, Daliang 2 Mary a Crown bakery | 15-7-13 |
| SA1029 | Sandwich | Sandwich | unpacked | farmer’s market/street vendors | >500g | 2015-06-05 | Heilongjiang | In jiamusi city | Tongjiang Street Department store South of Simon 20 Rice thumb cake world | Thumb cake world | 20150605 |
| SA1030 | Sandwich | Sandwich | unpacked | farmer’s market/street vendors | >500g | 2015-06-09 | Heilongjiang | In jixi city | AI Chi, guangyi Street Burger | Ai Keqi cake-homemade | 20150609 |
| SA1031 | Sandwich | Sandwich | prepacked | Supermarket/department stores | >500g | 2015-06-09 | Heilongjiang | In jixi city | Liverpool Road 71 New Mart supermarket | New Mart supermarket self- | 20150609 |
| SA1032 | Sandwich | Sandwich | unpacked | farmer’s market/street vendors | >500g | 2015-06-01 | Heilongjiang | Qitaihe city | Xuefu road in Saint Petersburg restaurants | Qitaihe city, Heilongjiang province, Saint Petersburg restaurants | 2015-6-1 |
| SA1033 | Sandwich | Sandwich | prepacked | Supermarket/department stores | >500g | 2015-08-19 | Heilongjiang | Suihua city | Zhongxing East Road, 228 Hua Chen, supermarket | Huachen supermarket | 20150818 |
| SA1034 | Sandwich | Sandwich | prepacked | Supermarket/department stores | >500g | 2015-08-11 | Henan | Puyang city | Intersection of Jiefang road and red Road South 100 M Pu Ke long supermarket | Hongqi road intersection South Jiefang road, Puyang in Puyang city 100 M Pu Ke long supermarket | 2015-08-11 |
| SA1035 | Sandwich | Sandwich | unpacked | farmer’s market/street vendors | >500g | 2015-06-15 | Hubei | In Yichang city | Wu Lin Lu Ai Menger hotel | Ai Meng's cake shop | 20150615 |
| SA1036 | Sandwich | Sandwich | unpacked | farmer’s market/street vendors | >500g | 2015-06-15 | Hubei | In Yichang city | Longzhoupingzhen Dragon Boulevard 37 TI-SI bakery baking | TI-baked think Pancake House | 20150615 |
| SA1037 | Sandwich | Sandwich | unpacked | farmer’s market/street vendors | >500g | 2015-09-07 | Jiangxi | JI ' an city | Crown Yangming Head Office | Crown Yangming Head Office | 2015-09-07 |
| SA1038 | Sandwich | Sandwich | unpacked | farmer’s market/street vendors | >500g | 2015-09-07 | Jiangxi | JI ' an city | Kaladuojian new world square | Kaladuojian new world square | 2015-09-07 |
| SA1039 | Sandwich | Sandwich | unpacked | farmer’s market/street vendors | >500g | 2015-07-21 | Jiangxi | Jiujiang city | De ' an Yong Xin road 138 Hongda cake shop | Hongda cake shop | 20150721 |
| SA1040 | Sandwich | Sandwich | unpacked | farmer’s market/street vendors | >500g | 2015-07-21 | Jiangxi | Jiujiang city | Zone de ' an pagoda one HIV/AIDS cake shop | More than one HIV-cake shop | 20150721 |
| SA1041 | Sandwich | Sandwich | unpacked | farmer’s market/street vendors | >500g | 2015-06-15 | Jiangxi | Yichun city | Construction of fengchuanzhen road 233 Its Endowment Brook bakery | Fengxin County Endowment Brook bakery | 20150615 |
| SA1042 | Sandwich | Sandwich | prepacked | Supermarket/department stores | >500g | 2015-09-28 | Zhejiang | In Lishui city | Yulei Rd RT supermarket | RT-supermarket | 20150824 |
| SA1043 | Sandwich | Sandwich | unpacked | farmer’s market/street vendors | >500g | 2015-09-07 | Zhejiang | Taizhou city | East Central Avenue 150 City lights shop 176 Silk micro-vascular pedicle | Unknown | 20150824 |
| SA1044 | Sandwich | Sandwich | unpacked | farmer’s market/street vendors | >500g | 2015-09-07 | Zhejiang | Taizhou city | Kaiyuan road 149 Thaci, Iraq | Unknown | 20150824 |
| SA1045 | Sandwich | Sandwich | unpacked | farmer’s market/street vendors | >500g | 2015-09-07 | Zhejiang | Taizhou city | East Central Avenue 120 Pacific building, 101 Yiming, really fresh milk | Unknown | 20150824 |
| SA1046 | Sandwich | Sandwich | unpacked | farmer’s market/street vendors | >500g | 2015-07-27 | Beijing | Huairou district | Fu Qian Street Huai ROU district 14 Wumart, Beijing-Peking University world a filet in the supermarket bakery | Fu Qian Street Huai ROU district 14 Wumart, Beijing-Peking University world a filet in the supermarket bakery | 15-7-27 |
| SA1047 | Sandwich | Sandwich | unpacked | farmer’s market/street vendors | >500g | 2015-08-10 | Sichuan | Chengdu city | Chongzhou on South Street 272 Anderson, Sichuan food co | Anderson, Sichuan food co | 20150810 |
| SA1048 | Meat and meat products | Meat and meat products | unpacked | farmer’s market/street vendors | >500g | 2015-06-16 | Anhui | Hefei city | Taobao Chaohu Lake specialty https://item. Taobao. com/item. htm? SPM=a1z10.5-c. w4002-2797617600.38. qlYHyq& id=40432383769 | Specialties in the chaohu Lake https://zhuo806986537.taobao.com/ | 20150613 |
| SA1049 | Meat and meat products | Meat and meat products | unpacked | farmer’s market/street vendors | >500g | 2015-08-03 | Anhui | Hefei city | Taobao gourmet American kitchen next door https://1014986132.taobao.com/ | Wisteria Taobao shop gourmet American kitchen next door https://1014986132.taobao.com/ | 20150802 |
| SA1050 | Meat and meat products | Meat and meat products | unpacked | farmer’s market/street vendors | >500g | 2015-05-04 | Hebei | Langfang city | 366 Online Mall the Langfang http://www.0316366.com/SuperMarket/product/Product.aspx?productID=P150417105180 | 366 Online Mall the Langfang | 20150504 |
| SA1051 | Meat and meat products | Meat and meat products | unpacked | farmer’s market/street vendors | >500g | 2015-05-19 | Hebei | Shijiazhuang | Taobao regional cuisine of Handan city http://item.taobao.com/item.htm?spm=a1z09.2.9.218.D2n5Sd&id=44055122218&_u=k1qj0lc52b17 | Homemade | 20150518 |
| SA1052 | Meat and meat products | Meat and meat products | unpacked | farmer’s market/street vendors | >500g | 2015-08-16 | Hebei | Shijiazhuang | Taobao Sanyi traditional gourmet https://item.taobao.com/item.htm?spm=a1z09.5.0.0.Ibg2vm&id=520603818871&_u=h1qj0lc5daf8 | Homemade | 2015-8-16 |
| SA1053 | Meat and meat products | Meat and meat products | unpacked | farmer’s market/street vendors | >500g | 2015-08-16 | Hebei | Shijiazhuang | Taobao lazy cow cake shop in Sichuan https://item.taobao.com/auction/noitem.htm?itemid=44720447039&catid=50008617&spm= a1z09.2.0.0.xCIQ59&_u=s1qj0lc5cfd5&mt=&itemid=44720447039&catid=50008617&spm= a1z09.2.0.0.xCIQ59&_u=s1qj0lc5cfd5 | Homemade | 2015-8-16 |
| SA1054 | Meat and meat products | Meat and meat products | unpacked | farmer’s market/street vendors | >500g | 2015-07-27 | Shaanxi | XI ' an city | Taobao to hang around the kitchen https://item.taobao.com/item.htm?spm=a1z09.2.9.38.xIgae4&id=43775383438&_u=s2bcqmba56b9 | Taobao: hang around the kitchen homemade | 20150727 |
| SA1055 | Meat and meat products | Meat and meat products | unpacked | farmer’s market/street vendors | >500g | 2015-07-27 | Shaanxi | XI ' an city | Taobao to hang around the kitchen https://item.taobao.com/item.htm?spm=a1z09.2.9.20.xIgae4&id=43849629546&_u=s2bcqmba4a45 | Taobao: hang around the kitchen homemade | 20150727 |
| SA1056 | Meat and meat products | Meat and meat products | unpacked | Supermarket/department stores | >500g | 2015-08-11 | Shaanxi | XI ' an city | Center Street 3 Long hair supermarket | Xian-Cheng Fung foods limited | 20150811 |
| SA1057 | Meat and meat products | Meat and meat products | unpacked | farmer’s market/street vendors | >500g | 2015-09-07 | Shaanxi | XI ' an city | Gaoling deer songs pot pot of sweet little hot pot | Unknown | 20150907 |
| SA1058 | Meat and meat products | Meat and meat products | unpacked | farmer’s market/street vendors | >500g | 2015-04-14 | Shaanxi | Xianyang city | Cross market Ma Jianping mutton and beef wholesale Department | No | 20150414 |
| SA1059 | Meat and meat products | Meat and meat products | unpacked | farmer’s market/street vendors | >500g | 2015-04-14 | Shaanxi | Xianyang city | Cross market John mutton and beef wholesale Department | No | 20150414 |
| SA1060 | Meat and meat products | Meat and meat products | unpacked | Supermarket/department stores | >500g | 2015-08-04 | Hunan | Yiyang city | Zijiang River Road and peach blossom road well run supermarket | Well run supermarket | 2015/8/4 |
| SA1061 | Meat and meat products | Meat and meat products | unpacked | farmer’s market/street vendors | >500g | 2015-05-18 | Hunan | In Yueyang city | Housing products http://gwyttc.taobao.com/ | Unknown | 20150824 |
| SA1062 | Meat and meat products | Meat and meat products | unpacked | farmer’s market/street vendors | >500g | 2015-05-19 | Hunan | In Yueyang city | Mediocre food shop http://shop111367659.taobao.com/ | Unknown | 15-5-19 |
| SA1063 | Meat and meat products | Meat and meat products | unpacked | farmer’s market/street vendors | >500g | 2015-07-08 | Shanghai | PuTuo district | Tai Huang taopu pot shop - Cedar road 338 | Unknown | 20150824 |
| SA1064 | Meat and meat products | Meat and meat products | unpacked | farmer’s market/street vendors | >500g | 2015-06-01 | Guangdong | Zhongshan city | Yu Fu Yuan on Taobao flagship store | Unknown | 20150527 |
| SA1065 | Meat and meat products | Meat and meat products | unpacked | farmer’s market/street vendors | >500g | 2015-06-02 | Heilongjiang | In jiamusi city | Taobao River http://item.taobao.com/item.htm?spm=a1z09.2.9.85.NoLZwX&id=26089176773&_u=k1qb46ff970 | Unknown | 20150602 |
| SA1066 | Meat and meat products | Meat and meat products | prepacked | farmer’s market/street vendors | >500g | 2015-09-06 | Heilongjiang | Mudanjiang city | Derivative: Hi Spicy Chinese kitchen Micro signal:hila597 | Derivative: Hi Spicy Chinese kitchen Micro signal:hila597 | 20150906 |
| SA1067 | Meat and meat products | Meat and meat products | unpacked | Supermarket/department stores | >500g | 2015-06-15 | Heilongjiang | Qiqihar | RT-supermarket | Unknown | 2015-06-15 |
| SA1068 | Meat and meat products | Meat and meat products | unpacked | farmer’s market/street vendors | >500g | 2015-04-06 | Hubei | Xiaogan city | Peng Jia Wan market Li Ya | Peng Jia Wan market Li Ya | 20140406 |
| SA1069 | Meat and meat products | Meat and meat products | unpacked | Supermarket/department stores | >500g | 2015-04-01 | Hubei | Wuhan City | Zhongbai storage luoshi Road branch | Our shop | 20150401 |
| SA1070 | Meat and meat products | Meat and meat products | unpacked | farmer’s market/street vendors | >500g | 2015-04-01 | Hubei | Wuhan City | Liu Chi food limited Wuhan luoshi road | Our shop | 20150401 |
| SA1071 | Meat and meat products | Meat and meat products | unpacked | Supermarket/department stores | >500g | 2015-06-08 | Hubei | Wuhan City | Rouge Lu Shuang Bai community 6 Discuss vendors | Wuhan jinxin source trading limited liability company | 15-6-8 |
| SA1072 | Meat and meat products | Meat and meat products | unpacked | farmer’s market/street vendors | >500g | 2015-07-13 | Hubei | Suizhou city | Peaceful and stable road farmers ' market 43 Halogen square in Wuhan | Unknown | 20150713 |
| SA1073 | Meat and meat products | Meat and meat products | unpacked | farmer’s market/street vendors | >500g | 2015-07-06 | Hubei | Wuhan City | Eisai Royal sweet stewed dish of fresh market store | Our shop | 20150706 |
| SA1074 | Meat and meat products | Meat and meat products | unpacked | farmer’s market/street vendors | >500g | 2015-04-07 | Jilin | Tonghua city | Zhao Guangming road cultural market flower stand | Dongchang district, tonghua dingxin slaughter limited | 15-4-7 |
| SA1075 | Meat and meat products | Meat and meat products | unpacked | farmer’s market/street vendors | >500g | 2015-04-07 | Jilin | Tonghua city | Palit road markets Ding beef stand | Tonghua food company | 15-4-7 |
| SA1076 | Meat and meat products | Meat and meat products | unpacked | farmer’s market/street vendors | >500g | 2015-04-07 | Jilin | Tonghua city | Unity Road East market | Jian Cheng source of livestock and poultry slaughtering, Ltd | 15-4-7 |
| SA1077 | Meat and meat products | Meat and meat products | unpacked | farmer’s market/street vendors | >500g | 2015-10-27 | Jiangxi | Fuzhou City | Huang Huang on lean store / Changzheng road | Bright bright group of Jiangxi food company limited | 2015.10.26 |
| SA1078 | Meat and meat products | Meat and meat products | unpacked | farmer’s market/street vendors | >500g | 2015-10-15 | Jiangxi | In Nanchang city | Tao Tao food https://item.taobao.com/item.htm?spm=a1z10.1-c.w6920587-1506482234.1.bF1plM&id=23351728796 | On Nanchang of Jiangxi province Huang Huang | 20151014 |
| SA1079 | Meat and meat products | Meat and meat products | unpacked | Supermarket/department stores | >500g | 2015-08-25 | Jiangxi | In Ganzhou city | Lianjiang Avenue in kerqin | Inner Mongolia keerqin cattle industry company limited | 20141017 |
| SA1080 | Meat and meat products | Meat and meat products | unpacked | farmer’s market/street vendors | >500g | 2015-10-26 | Jiangxi | In Nanchang city | AI Lake farmer's market, high-tech district, Nanchang city, Jiangxi province, Anhui guy halogen Chen family of butcher's shop | AI Lake farmer's market, high-tech district, Nanchang city, Jiangxi province, Anhui guy halogen Chen family of butcher's shop | 20151026 |
| SA1081 | Meat and meat products | Meat and meat products | unpacked | farmer’s market/street vendors | >500g | 2015-10-26 | Jiangxi | In Nanchang city | AI Lake farmer's market, high-tech district, Nanchang city, Jiangxi province, Anhui guy halogen Chen family of butcher's shop | AI Lake farmer's market, high-tech district, Nanchang city, Jiangxi province, Anhui guy halogen Chen family of butcher's shop | 20151026 |
| SA1082 | Meat and meat products | Meat and meat products | unpacked | farmer’s market/street vendors | >500g | 2015-10-26 | Jiangxi | Jingdezhen city | Lotus Villa market | Lotus Villa market | 20151026 |
| SA1083 | Meat and meat products | Meat and meat products | unpacked | farmer’s market/street vendors | >500g | 2015-10-26 | Jiangxi | Jingdezhen city | Lotus Villa market | Lotus Villa market | 20151026 |
| SA1084 | Meat and meat products | Meat and meat products | unpacked | farmer’s market/street vendors | >500g | 2015-09-11 | Jiangxi | Jingdezhen city | Shop name: Wen Wen mother handmade pasta shop https://shop112843078.taobao.com/?spm=a230r.7195193.1997079397.2.HCuEo3 Taobao number: I am a big beautiful girls 58 | Shop name: Wen Wen mother handmade pasta shop Taobao number: I am a big beautiful girl 58 | 20150908 |
| SA1085 | Meat and meat products | Meat and meat products | unpacked | farmer’s market/street vendors | >500g | 2015-10-26 | Jiangxi | Yichun city | Before the in fengxin County fengchuanzhen East Gate market channel 3 Shop | Before the in fengxin County fengchuanzhen East Gate market channel 3 Shop | 2015.10.26 |
| SA1086 | Meat and meat products | Meat and meat products | unpacked | farmer’s market/street vendors | >500g | 2015-09-06 | Zhejiang | Hangzhou City | Micro store: Chong dad hand food micro-signals: tmdx1009 | Micro store: Chong dad hand food micro-signals: tmdx1009 | 15-9-6 |
| SA1087 | Meat and meat products | Meat and meat products | unpacked | farmer’s market/street vendors | >500g | 2015-09-06 | Zhejiang | Hangzhou City | Online shopping | Micro store: Chong dad hand food micro-signals: tmdx1009 | 15-9-6 |
| SA1088 | Meat and meat products | Meat and meat products | unpacked | farmer’s market/street vendors | >500g | 2015-08-24 | Xinjiang | Urumqi city | Farmers in Sichuan province https://item.taobao.com/item.htm?spm=a1z09.2.0.0.tF3UhH&id=9399493590&_u=9f63c35503c | Farmers in Sichuan province | 20150817 |
| SA1089 | Meat and meat products | Meat and meat products | unpacked | Supermarket/department stores | >500g | 2015-07-27 | Beijing | Xicheng District | Wumart, Beijing xinjiekou store | Changchun, Jilin province, haoyue Muslim meat industry company limited | 20150727 |
| SA1090 | Meat and meat products | Meat and meat products | unpacked | farmer’s market/street vendors | >500g | 2015-05-13 | Jiangsu | In Nanjing city | Taobao Nanjing friends souvenir shop | Nanjing friends souvenir shop | 20150512 |
| SA1091 | Meat and meat products | Meat and meat products | unpacked | farmer’s market/street vendors | >500g | 2015-09-21 | Jiangsu | In Nanjing city | Tao baohuantian | Huan Tian XI | 20150922 |
| SA1092 | Meat and meat products | Meat and meat products | unpacked | farmer’s market/street vendors | >500g | 2015-09-07 | Jiangsu | In Nanjing city | Taobao - Bite of Nanjing Nanjing soil pig restaurant | Taobao - Bite of Nanjing Nanjing soil pig restaurant | 20150907 |
| SA1093 | Meat and meat products | Meat and meat products | unpacked | farmer’s market/street vendors | >500g | 2015-09-14 | Gansu | Gannan Tibetan Autonomous Prefecture | Taobao Mary villeggiatura (Northwest specialty store) ( https://item.taobao.com/item.htm?spm=a1z09.2.0.0.Ex3wNY&id=39055953070&_u=fntm61k5d1d ) | Taobao Mary villeggiatura (Northwest specialty store) | 20150909 |
| SA1094 | Eggs and egg-products | Eggs and egg-products | unpacked | farmer’s market/street vendors | >500g | 2015-06-29 | Anhui | Hefei city | Jinzhai road 136 Hollyland cake jinzhai road, joy City store | Hollyland cake jinzhai road, joy City store | 20150629 |
| SA1095 | Eggs and egg-products | Eggs and egg-products | unpacked | Supermarket/department stores | >500g | 2015-06-29 | Anhui | Hefei city | Wang Jiang Road and the road of cooperation resources colorful city, China resources ' suguo supermarket (River Road) | China resources ' suguo supermarket (River Road) | 20150628 |
| SA1096 | Eggs and egg-products | Eggs and egg-products | unpacked | farmer’s market/street vendors | >500g | 2015-06-29 | Anhui | Hefei city | GE shop the markets, baohe road and qimen road Kee cake shop | GE shop the markets keep a cake shop | 20150628 |
| SA1097 | Eggs and egg-products | Eggs and egg-products | unpacked | farmer’s market/street vendors | >500g | 2015-07-20 | Anhui | Hefei city | Taobao Milan cake http://shop104623959.taobao.com/ | Taobao Milan cake http://shop104623959.taobao.com/ | 2015-07-20 |
| SA1098 | Eggs and egg-products | Eggs and egg-products | prepacked | farmer’s market/street vendors | >500g | 2015-08-24 | Anhui | Hefei city | PI River Road 9 XING building, first floor, sanli market East Gate South of Xu Palace which makes semi-finished shop | Kee Ting Xu, which makes semi-finished shop | 20150823 |
| SA1099 | Eggs and egg-products | Eggs and egg-products | unpacked | farmer’s market/street vendors | >500g | 2015-07-22 | Shanxi | In jinzhong city | Nan shan Nan lu Jiao Xiao Mei West bakery | Nan shan Nan lu Jiao Xiao Mei West bakery | 15-7-22 |
| SA1100 | Eggs and egg-products | Eggs and egg-products | prepacked | farmer’s market/street vendors | >500g | 2015-08-03 | Shanxi | Luliang city | Gate of the fen line between four high school snack | Fenyang IV high school door snack | 2015-08-03 |
| SA1101 | Eggs and egg-products | Eggs and egg-products | unpacked | farmer’s market/street vendors | >500g | 2015-08-03 | Shanxi | Yangquan city | Nanshan road hollyland shop | Hollyland shop | 20150803 |
| SA1102 | Eggs and egg-products | Eggs and egg-products | unpacked | farmer’s market/street vendors | >500g | 2015-08-24 | Hebei | In Chengde city | Chengde's second high school College Road junction | Unknown | 20150824 |
| SA1103 | Eggs and egg-products | Eggs and egg-products | unpacked | farmer’s market/street vendors | >500g | 2015-06-29 | Yunnan | Xishuangbanna Dai autonomous prefecture | Manting Lu 1 Crown cake jinglan shop | Crown cake, Jinghong city Jinglan shop | 20150629 |
| SA1104 | Eggs and egg-products | Eggs and egg-products | unpacked | Supermarket/department stores | >500g | 2015-06-08 | Liaoning | Benxi City | Yong Sheng Jie Bai Xiang Lin Yangtze River branch | Passion fruit forest cake shop (Yangtze River Branch) | 20150608 |
| SA1105 | Eggs and egg-products | Eggs and egg-products | unpacked | farmer’s market/street vendors | >500g | 2015-06-08 | Liaoning | Huludao city | Long Cheng Jie 28 Lou DE Grand Grill store | Long range Street, Longgang District, Huludao city 28 Lou DE Grand Grill store | 2015-06-08 |
| SA1106 | Eggs and egg-products | Eggs and egg-products | unpacked | Supermarket/department stores | >500g | 2015-06-23 | Inner Mongoria | Baotou city | Gawler road, Han Qing BA huide garden huide integrated louyongsheng into the supermarket shop | Yongsheng huide into supermarket shop | 20150623 |
| SA1107 | Eggs and egg-products | Eggs and egg-products | prepacked | farmer’s market/street vendors | >500g | 2015-07-04 | Inner Mongoria | Xilin GOL League | Xilin Chun chun Lin Yi fang Street cake shop | Xilin Chun chun Lin Yi fang Street cake shop | 2015-07-04 |
| SA1108 | Eggs and egg-products | Eggs and egg-products | prepacked | farmer’s market/street vendors | >500g | 2015-06-15 | Shaanxi | In Ankang city | Feng Yuanjing of Bashan East Europe shop | Our shop | 2015.6.15 |
| SA1109 | Eggs and egg-products | Eggs and egg-products | unpacked | farmer’s market/street vendors | >500g | 2015-07-14 | Shaanxi | In Ankang city | Ou Feng Park East Avenue store | Ankangoufeng garden food co | 2015.7.13 |
| SA1110 | Eggs and egg-products | Eggs and egg-products | unpacked | farmer’s market/street vendors | >500g | 2015-06-16 | Shaanxi | Baoji city | Qi shankai Commander bakery | Qi shankai Commander cake shop | 20150616 |
| SA1111 | Eggs and egg-products | Eggs and egg-products | unpacked | farmer’s market/street vendors | >500g | 2015-08-04 | Shaanxi | Baoji city | Pui new language (bakery) | Pui new language (bakery) | 20150804 |
| SA1112 | Eggs and egg-products | Eggs and egg-products | unpacked | farmer’s market/street vendors | >500g | 2015-06-01 | Shaanxi | Yanan city | Sha Liang Jie, thousands of fragrant | Thousands of fragrant cake shop | 20150601 |
| SA1113 | Eggs and egg-products | Eggs and egg-products | unpacked | farmer’s market/street vendors | >500g | 2015-06-01 | Shaanxi | Yanan city | Sha Liang Jie, thousands of fragrant | Thousands of fragrant cake shop | 20150601 |
| SA1114 | Eggs and egg-products | Eggs and egg-products | unpacked | farmer’s market/street vendors | >500g | 2015-08-04 | Shaanxi | Yanan city | Center Street in McCormick | McCormick | 20150804 |
| SA1115 | Eggs and egg-products | Eggs and egg-products | unpacked | farmer’s market/street vendors | >500g | 2015-08-03 | Shaanxi | Yanan city | Street Bank | Ports | 20150803 |
| SA1116 | Eggs and egg-products | Eggs and egg-products | unpacked | farmer’s market/street vendors | >500g | 2015-06-09 | Fujian | In sanming city | Sweet Sally Pancake House | Sweet Sally Pancake House | 20150609 |
| SA1117 | Eggs and egg-products | Eggs and egg-products | prepacked | farmer’s market/street vendors | >500g | 2015-06-09 | Fujian | In sanming city | Eight junctions 1 | Yan Rong confectionery factory | 20150609 |
| SA1118 | Eggs and egg-products | Eggs and egg-products | unpacked | farmer’s market/street vendors | >500g | 2015-06-09 | Fujian | In sanming city | Sachet Wang shop | Sachet Wang shop | 20150609 |
| SA1119 | Eggs and egg-products | Eggs and egg-products | unpacked | Supermarket/department stores | >500g | 2015-06-08 | Shandong | In linyi city | Lan Ling Jin Ling Zhen Jia Zhuang Street | Jia Le Fu cake | 15-6-8 |
| SA1120 | Eggs and egg-products | Eggs and egg-products | unpacked | Supermarket/department stores | >500g | 2015-08-10 | Shandong | Rizhao city | Zhengyang road and the sea song/f, road department store supermarket | Department store | 15-8-10 |
| SA1121 | Eggs and egg-products | Eggs and egg-products | unpacked | farmer’s market/street vendors | >500g | 2015-06-23 | Shandong | Dezhou city | North West section of South Street cake shop | Arc de Triomphe bakery | 15-6-23 |
| SA1122 | Eggs and egg-products | Eggs and egg-products | unpacked | farmer’s market/street vendors | >500g | 2015-07-07 | Shandong | Dezhou city | ABC South West South Street 50 Mai Mai Xiang Yuan cake shop | Wheat cake shop | 15-7-7 |
| SA1123 | Eggs and egg-products | Eggs and egg-products | unpacked | farmer’s market/street vendors | >500g | 2015-06-12 | Shanghai | Xuhui district, | Christine Ling Ling Road | Shanghai Liz Ting foods limited | 15-6-11 |
| SA1124 | Eggs and egg-products | Eggs and egg-products | unpacked | farmer’s market/street vendors | >500g | 2015-06-12 | Shanghai | Xuhui district, | Christine Ling Ling Road | Shanghai Liz Ting foods limited | 15-6-9 |
| SA1125 | Eggs and egg-products | Eggs and egg-products | prepacked | farmer’s market/street vendors | >500g | 2015-07-07 | Guangdong | Foshan City | Wei road, Temple Street building of Cantonese Dumbo | Unknown | 15-7-7 |
| SA1126 | Eggs and egg-products | Eggs and egg-products | unpacked | farmer’s market/street vendors | >500g | 2015-06-02 | Heilongjiang | Hegang city | XING Shan Lu Hong Yun cake workshop | Hongyun cake workshop | 2015.6.2 |
| SA1127 | Eggs and egg-products | Eggs and egg-products | unpacked | Supermarket/department stores | >500g | 2015-07-13 | Hubei | Suizhou city | Yongyang Avenue light supermarket | Light cake workshop | 20150713 |
| SA1128 | Eggs and egg-products | Eggs and egg-products | unpacked | farmer’s market/street vendors | >500g | 2015-07-13 | Hubei | Suizhou city | Main Street East 2 Ink pad, Gisli cake shop next to the mountain Plaza | Gisli cake workshop | 20150712 |
| SA1129 | Eggs and egg-products | Eggs and egg-products | unpacked | farmer’s market/street vendors | >500g | 2015-07-13 | Hubei | Suizhou city | Air South Road opposite the women's and children's Golden Apple Cake | Golden Apple Cake | 20150713 |
| SA1130 | Eggs and egg-products | Eggs and egg-products | unpacked | farmer’s market/street vendors | >500g | 2015-06-21 | Jilin | Siping city | Gifts to birthday cake shop | Unknown | 15-6-21 |
| SA1131 | Eggs and egg-products | Eggs and egg-products | unpacked | farmer’s market/street vendors | >500g | 2015-08-18 | Jiangxi | In Ganzhou city | Lianjiangzhen Phoenix Avenue, King's Crown bakery | Xingguo County, King's Crown bakery | 20150818 |
| SA1132 | Eggs and egg-products | Eggs and egg-products | unpacked | farmer’s market/street vendors | >500g | 2015-04-13 | Jiangxi | Jingdezhen city | Zhong Shan Nan lu 26 Taste bakery | Zhong Shan Nan lu 26 Taste bakery | 20150413 |
| SA1133 | Eggs and egg-products | Eggs and egg-products | unpacked | farmer’s market/street vendors | >500g | 2015-08-17 | Jiangxi | In Nanchang city | Shan da DAO XI road intersection, with three stores Calado Carrefour store | Nanchang new food limited | 20150817 |
| SA1134 | Eggs and egg-products | Eggs and egg-products | unpacked | farmer’s market/street vendors | >500g | 2015-08-17 | Jiangxi | In Nanchang city | New Creek West Road and new bridge road intersection fresh guangx I breakfast points | No | 20150817 |
| SA1135 | Eggs and egg-products | Eggs and egg-products | unpacked | farmer’s market/street vendors | >500g | 2015-08-18 | Beijing | Xicheng District | How beautiful (XI Zhi men NEI da Jie, Xicheng District Beijing 184 Number) | How beautiful Beijing taste food limited liability company (xizhimen) | 20150818 |
| SA1136 | Eggs and egg-products | Eggs and egg-products | unpacked | farmer’s market/street vendors | >500g | 2015-07-15 | Jiangsu | In Nanjing city | Jiangsu road 60-3 Beautiful cake shop | Beautiful cake shop | 20150715 |
| SA1137 | Milk-products | Milk-products | prepacked | farmer’s market/street vendors | >500g | 2015-05-14 | Hebei | Shijiazhuang | Zhongshan Road West second ring junction Army Day fresh milk | Homemade | 20150514 |
| SA1138 | Milk-products | Milk-products | prepacked | farmer’s market/street vendors | >500g | 2015-05-14 | Hebei | Shijiazhuang | Zhaiying Street, pasture hut | Homemade | 20150514 |
| SA1139 | Milk-products | Milk-products | prepacked | Supermarket/department stores | >500g | 2015-05-12 | Liaoning | In Shenyang city | The East shuncheng Street 100 East Deputy fresh food supermarket | Liaoning huishan dairy group (Shenyang) limited | 15-5-12 |
| SA1140 | Milk-products | Milk-products | unpacked | farmer’s market/street vendors | >500g | 2015-06-01 | Shaanxi | XI ' an city | Bridge gate of the beautiful garden district, new South 20 M happy pasture milk | Joy farm fresh milk production | 20150601 |
| SA1141 | Milk-products | Milk-products | prepacked | farmer’s market/street vendors | >500g | 2015-05-28 | Heilongjiang | Hegang city | Guangming road revitalization of the square grass source of fresh milk | D source of fresh milk | 2015.5.28 |
| SA1142 | Milk-products | Milk-products | prepacked | farmer’s market/street vendors | >500g | 2015-06-01 | Gansu | Gannan Tibetan Autonomous Prefecture | When Daming, Zhou Jie min trade building grocery stores | Lanzhou shoton dairy, Ltd | 20150521 |
| SA1143 | Condiments | Condiments | unpacked | farmer’s market/street vendors | >500g | 2015-05-07 | Sichuan | Panzhihua city | Gate of the second elementary school in panzhihua city street vendors | Unknown | 2015-05-07 |
| SA1144 | Condiments | Condiments | prepacked | farmer’s market/street vendors | >500g | 2015-04-15 | Jilin | Siping city | Tie bei supermarket North of lily 50 M PFAFF cake shop | Tie bei supermarket North of lily 50 M PFAFF homemade cake shop | 15-4-15 |
| SA1145 | Condiments | Condiments | prepacked | Supermarket/department stores | >500g | 2015-04-13 | Jilin | Tonghua city | Meihekou city, Jianguo Road Euro-Asian supermarket | Anhui yo Orchard Group limited | 2015/02/07 |
| SA1146 | Bean-products | Bean-products | prepacked | Supermarket/department stores | >500g | 2015-07-06 | Hebei | Langfang city | Channel and yongding road intersection at the southwest corner of Government glory supermarkets | Foshan Haitian (smart) seasoning food co | 20150101 |
| SA1147 | Bean-products | Bean-products | prepacked | farmer’s market/street vendors | >500g | 2015-06-01 | Shaanxi | XI ' an city | The third home of the east village integrated market in the east of the road, the east of the village | Jiangsu food limited company | 20150406.1 |
| SA1148 | Fruit desserts | Fruit desserts | prepacked | farmer’s market/street vendors | >500g | 2015-09-06 | Sichuan | Chengdu city | Xiao Dong Jie chongzhou xiaoge mala Tang | Unknown | 2015-09-06 |
| SA1149 | Fruit desserts | Fruit desserts | prepacked | farmer’s market/street vendors | >500g | 2015-07-15 | Hubei | Wuhan City | MOM and boy world of fondant http://item.taobao.com/item.htm?spm=a1z09.2.9.164.uaIvZN&id=39539108442&_u=d2dmgcdfe0c | Our shop | 15-6-21 |
| SA1150 | Fruit desserts | Fruit desserts | prepacked | farmer’s market/street vendors | >500g | 2015-07-15 | Hubei | Wuhan City | MOM and boy world of fondant http://item.taobao.com/item.htm?spm=a1z09.2.9.182.uaIvZN&id=40223628225&_u=d2dmgcd6a52 | Our shop | 15-7-2 |
